# Diverse and Selective Metal–Ligand Cooperative Routes for Activating Non-Functionalized Ketones

**DOI:** 10.1021/acs.inorgchem.4c03214

**Published:** 2025-01-31

**Authors:** Carlos Ferrer-Bru, Joaquina Ferrer, Vincenzo Passarelli, Fernando J. Lahoz, Pilar García-Orduña, Daniel Carmona

**Affiliations:** † Departamento de Catálisis y Procesos Catalíticos, Instituto de Síntesis Química y Catálisis Homogénea (ISQCH), CSIC - Universidad de Zaragoza, Pedro Cerbuna 12, 50009 Zaragoza, Spain

## Abstract

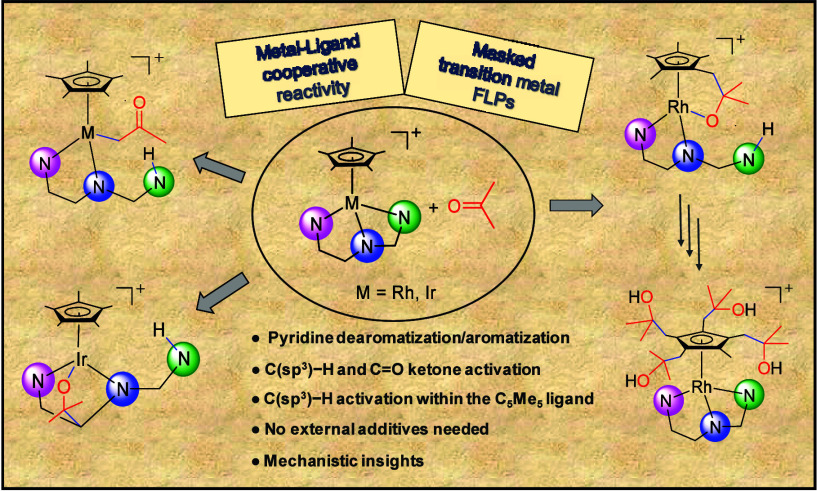

The rhodium and iridium complexes [Cp*M(κ^3^*N*,*N′*,*N″*-**L**)][SbF_6_] (Cp* = η^5^-C_5_Me_5_; M = Rh, **1**; Ir, **2**; **HL** = pyridinyl-amidine ligand) exhibit three different cooperative metal–ligand reactivity modes when interacting with nonfunctionalized ketones. With the methyl ketones CH_3_COR (R = CH_3_, Ph, CF_3_), activation of the ketone methyl C(sp^3^)–H bond yields ketonyl compounds of formula [Cp*M(CH_2_COR)(κ^2^*N*,*N′*-**HL**)][SbF_6_]. With the ketones (CF_3_)_2_CO and CF_3_COPh, the complexes add to the C=O double bond of the ketone. The addition of the iridium compound **2** occurs across the metal atom and the exocyclic carbon of the dearomatized pyridinyl moiety, and that of the rhodium analogue **1** takes place through the rhodium atom and the exocyclic methylene carbon of the Cp* ligand of the intermediate fulvene complex. In the rhodium case, the resulting metal-alkoxide derivative evolves to give rise to rhodium derivatives containing up to four added ketone molecules. In all of these processes, no additives are required, rendering them atom 100% efficient procedures for bond activation. From a mechanistic point of view, DFT calculation reveals that the diverse and selective behavior of **1** and **2** toward ketones can be explained by invoking three different intermediates, each driving the process through distinct reaction pathways.

## Abstract 2

The latent TMFLPs schematized in the graph activate the C−H bond in methyl ketones and add to the C=O bond of nonenolizable ketones with electron-withdrawing substituents. These reactions follow reactivity models in which metal−ligand cooperation occurs. Both experimental and theoretical studies support the implication of active FLP species, intermediates with the pyridine ring of one of the ligands in a dearomatized state, and fulvene complexes.

## Introduction

**Scheme 1:**
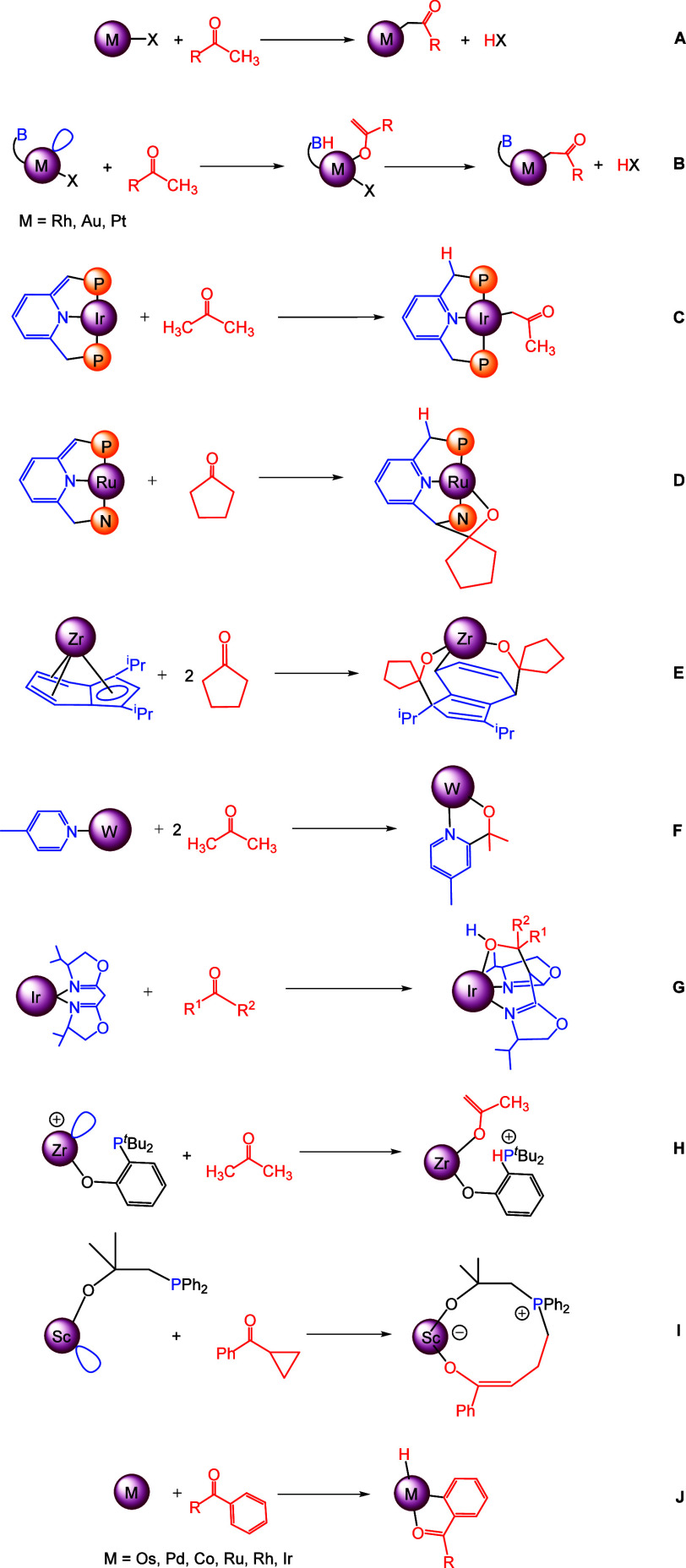
Selected Reactions of Ketones with Metallic Species (A) C(sp^3^)–H bond activation of methyl ketones. (B) C(sp^3^)–H bond activation of methyl ketones by metal–ligand cooperation reactivity. (C) C(sp^3^)–H bond activation of methyl ketones by reversible aromatization/dearomatization reactivity of a pyridine containing ligand. (D) C=O bond activation by reversible aromatization/dearomatization of a pyridine containing ligand. (E) C=O bond activation of pentanone by zirconium indenyl compounds. (F) C=O bond activation of acetone by tungsten picoline compounds. (G) C=O bond activation of ketones by iridium compounds. (H) C=O bond activation of acetone by zirconium/phosphorus TMFLPs. (I) C=O bond activation of cyclopropyl phenyl ketone by a Sc/P TMFLP compound. (J) *ortho*-C(sp^2^)–H bonds activation in aromatic ketones.

The interaction between ketones and metallic species leads to metalated derivatives that serve as active key reagents and/or intermediates in synthetic organic^1^ and biological^2^ transformations. One of the most explored approaches for preparing metalated ketones involves the C(sp^3^)–H bond activation of methyl ketones by transition metal complexes. As the most representative strategies, ketonyl complexes are formed by the direct reaction of simple methyl ketones with hydroxo complexes,^3^ with halogenated complexes, in the presence of K_2_CO_3_, Ag_2_O, KOH, or NaOH,^4^ or by a water-induced reaction with alkene-Ir(I) or alkyne-Pt(0) complexes^5,6^ (Scheme 1A).

An elegant alternative approach for abstracting a weakly acidic C(sp^3^)–H hydrogen atom adjacent to a carbonyl group involves the use of metallic compounds that feature an accessible vacant site on the metal and a basic site on one of their ligands, thereby possessing an internal base. The metal center, acting as a Lewis acid, coordinates the ketone oxygen and the basic site on the ligand, functioning as a Brønsted base, abstracts a proton from the methyl group of the ketone. Rapid equilibration between the O–metal and C–metal bound isomers,^7^ along with proton transfer to the X ligand and its consequent elimination as HX, completes the process (Scheme 1B). This methodology, which entails efficient metal–ligand cooperation, has been employed to synthesize acetonyl derivatives of rhodium,^8^ gold,^9^ and platinum.^10^

In the study of ketone activation by transition metal complexes, the use of reactivity strategies based on metal–ligand cooperation is much broader. For example, an iridium complex with a PNP pincer ligand, in which the metal is anchored to a pyridine-based moiety (Scheme 1C), activates one of the C(sp^3^)–H bonds of the acetone through cooperative reactivity modes based on reversible aromatization/dearomatization reactivity.^11^ The process involves cooperation between the metal and the pincer ligand without change in the formal metal oxidation state.^12^

By reversible aromatization/dearomatization of pyridine containing ligands, it is possible to carry out the addition of metallic fragments to the C=O bond of ketones as well. Thus, the ruthenium NNP pincer outlined in Scheme 1D adds to the cyclopentanone carbonyl forming a C–C bond between the carbonyl carbon and the nitrogen arm of the pincer ligand and a bond of the metal with the carbonyl oxygen.^13^

Through metal–ligand cooperation mechanisms, the addition of two molecules of cyclopentanone to zirconium indenyl compounds also occurs, resulting in the insertion of two molecules of ketone into positions 1 and 4 of the indenyl ligand (Scheme 1E).^14^ Similarly, the interaction of acetone with W(NPh)(*o*-(Me_3_SiN)_2_C_6_H_4_)(pic)_2_ provides the azaoximetalacycle W(NPh)(*o*-(Me_3_SiN)_2_C_6_H_4_)(OCH(Me)_2_(OC(Me)_2_-*o*-C_5_H_3_N-*p*-Me) as a result of its insertion into the *ortho* C–H bond of picoline (Scheme 1F),^15^ or the reversible insertion of ketones into the C(sp^3^)–H bond of a bis(oxazoline) ligand in iridium(III) complexes occurs (Scheme 1G).^16^

Activation of ketones has also been achieved through the use of transition metal frustrated Lewis pairs (TMFLPs). The zirconium/phosphorus FLP depicted in Scheme 1H reacts with acetone to yield a Zr-enolate bearing a phosphonium center,^17^ and neutral scandium/phosphorus FLPs react with cyclopropyl phenyl ketone, giving rise to a formal Sc/P 1,5-addition of the cyclopropyl ketone substrate (Scheme 1I).^18^

Finally, although somewhat distant from the objectives of this work, the activation of *ortho*-C(sp^2^)–H bonds in aromatic ketones rendering *O*-coordinated orthometalated ketones (Scheme 1J) deserves some commentary here. It constitutes a key stage in the functionalization of this class of ketones,^1a,1d^ making it a valuable tool in organic synthesis.

This type of activation has been reported for a variety of transition metal complexes bearing osmium,^19^ palladium,^20^ cobalt,^21^ ruthenium,^22^ rhodium,^23^ and iridium.^24^

**Chart 1:**
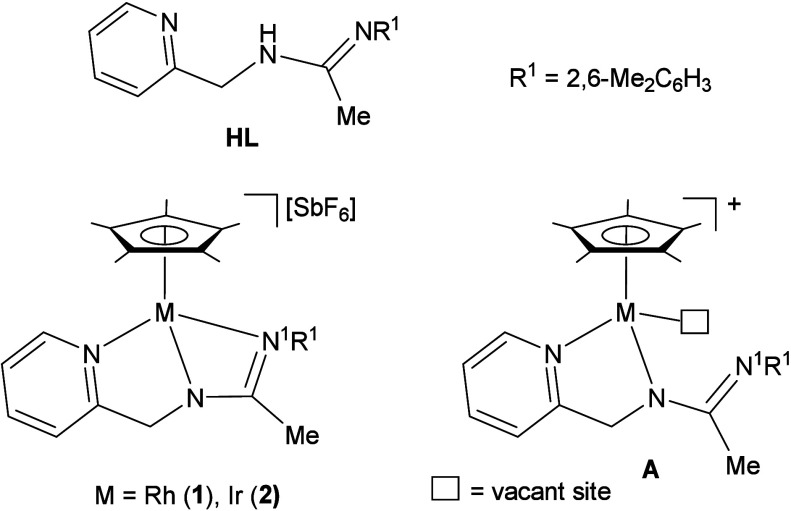
Pyridinyl-Amidine Ligand HL and Pyridinyl-Amidinato Complexes **1** and **2**

Recently, we reported the synthesis and characterization of the Cp*Rh(III) and Cp*Ir(III) (Cp* = η^5^-C_5_Me_5_) species **1** and **2**, based on the pyridinyl-amidine ligand **HL** depicted in Chart 1. The molecular structure of these compounds reveals the presence of a highly strained four-membered M–N–C–N^1^ metalacycle. Consequently, the heterolytic cleavage of the M–N^1^ bond is a low energy-demanding process that makes it easy to access reactive species of type **A** and related guanidine systems (Chart 1).^25^ The concurrent presence of both an acidic center (due the coordination vacant on the metal) and a basic center (the uncoordinated nitrogen atom) allows us to classify species **A** as TMFLP. Therefore, compounds **1** and **2** can be regarded as masked TMFLPs,^26^ and indeed, these complexes cleave the molecule of dihydrogen in a heterolytic manner, rendering metal hydrides containing an NH group.^25^

Given the accessibility of ketone activation through metal–ligand cooperation strategies, we contemplated the possibility that compounds **1** and **2** could activate ketones through mechanisms of this kind. From the initial experiments, we became aware of the rich reactivity that these compounds exhibited. We realized that by changing either the metal or the ketone the reactivity pattern changed and, consequently, the nature of the resulting products. Furthermore, the choice of solvent influenced the progression of certain reactions. Therein, we report on the diverse metal–ligand cooperative pathways for the activation of simple ketones shown by complexes **1** and **2**. Special attention is devoted to the ascertainment of the distinct intermediates involved, in order to explain the evolution of the observed reactions and the nature of the resulting products. Reaction mechanisms for the different processes are proposed based on experimental measurements, X-ray diffraction determinations, and DFT calculations.

## Results and Discussion

### Reaction with Methyl Ketones

^1^H NMR spectroscopy reveals the clean formation of two isomers upon the reaction of the complexes [Cp*M(κ^3^*N*,*N′*,*N″*-**L**)][SbF_6_] (Cp* = η^5^-C_5_Me_5_; M = Rh, **1**; Ir, **2**; **HL** = pyridinyl-amidine ligand) with methyl ketones CH_3_COR (R = CH_3_, Ph, CF_3_), as illustrated in eq 1. After several hours of reaction, either in the ketone itself or ketone/CD_2_Cl_2_ (50/50, v/v) mixtures as the solvent, strongly temperature dependent equilibria between the corresponding starting complex and each one of the two derived product were achieved (see Table S1). From the solution, mixtures containing starting compound and products were isolated (see Table S2) and analyzed by IR and NMR spectroscopies. As common spectroscopic features for the new compounds, the ^1^H NMR spectra show two signals coupled to each other in the region 2.2–3.6 ppm, attributed to two diastereotopic protons, while the ^13^C{^1^H} NMR spectra display a resonance in the range of 10.8 to 31.3 ppm. For the rhodium compounds **3**, **5**, and **7**, the latter resonance undergoes splitting due to coupling to the ^103^Rh nucleus with a constant of around 23 Hz. Furthermore, the ^13^C{^1^H} NMR spectra show a resonance in the 196–219 ppm region, and the IR spectra exhibit a strong band at about 1680 cm^–1^. All of these spectroscopic data together indicate the presence of a CH_2_C=O moiety coordinated to the metal in the new compounds.^3a,12^ A singlet around 7.8 ppm in the ^1^H NMR spectra and a broad band centered about 3300 cm^–1^ in the IR spectra are assigned to an NH functionality. The additionally, NMR signals attributed to the corresponding R substituent of the ketone are also observed. For example, a singlet around 1.1 ppm appears in the ^1^H NMR spectra of the products **3** and **4** derived from acetone (R = CH_3_), and the ^19^F NMR spectra of compounds **7** and **8** (R = CF_3_) show the presence of a singlet around −79.5 ppm. Based on these spectroscopic data (see Experimental Section for full details), we propose that the new compounds are two isomers of the product resulting from the activation of a methyl C(sp^3^)–H bond of the ketone with concomitant formation of M–C and N–H bonds (eq 1).

Regarding the origin of the isomerism of the products, NOE measurements allow proposing the structure of each of the two isomeric adducts. For one of them, irradiation of the amidine methyl or the NH protons enhances the CH_2_N or CH_2_CO methylene resonances, respectively. For the other, irradiation of the amidine methyl or the NH protons enhances the CH_2_CO or CH_2_N methylene resonances, respectively (see Figures S6 and S7, SI). Taking into account these data, we propose that the observed products are the *Z* and *E* isomers around the C=N double bond of the coordinated amidine.
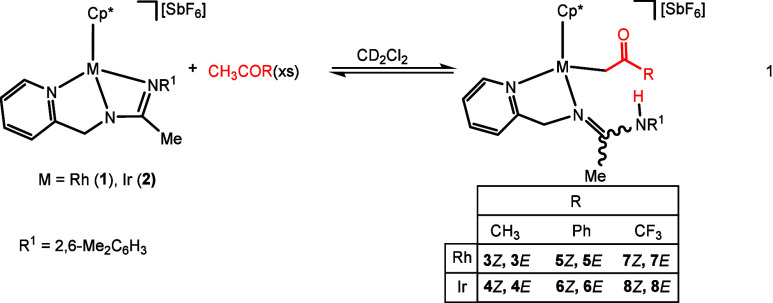
1

The equilibrium molar ratio for the reaction depicted in eq 1 was measured at different temperatures by ^1^H NMR spectroscopy. In particular, for the equilibria **1** ⇄ **7***Z* and **1** ⇄ **7***E*, the values of the *K*_obs_ were determined in the temperature interval 273–313 K. A van’t Hoff analysis gave values of Δ*G*° = −0.54 ± 0.83 kcal·mol^–1^, Δ*H*° = −10.06 ± 0.41 kcal·mol^–1^, and Δ*S*° = −31.94 ± 1.39 cal·mol^–1^·K^–1^ for the equilibrium **1** ⇄ **7***Z* and of Δ*G*° = −1.14 ± 0.34 kcal·mol^–1^, Δ*H*° = −10.88 ± 0.16 kcal·mol^–1^, and Δ*S*° = −32.70 ± 0.60 cal·mol^–1^·K^–1^ for the equilibrium **1** ⇄ **7***E* (see Table S3, Figures S8 and S9, SI). Thus, the acetonyl products are favored enthalpically but not entropically. The Δ*G*° values close to zero are consistent with the observation by NMR spectroscopy of both products and reactants. The negative Δ*S*° value causes the products to be favored when temperature is lowered as it was experimentally observed.

**Figure 1:**
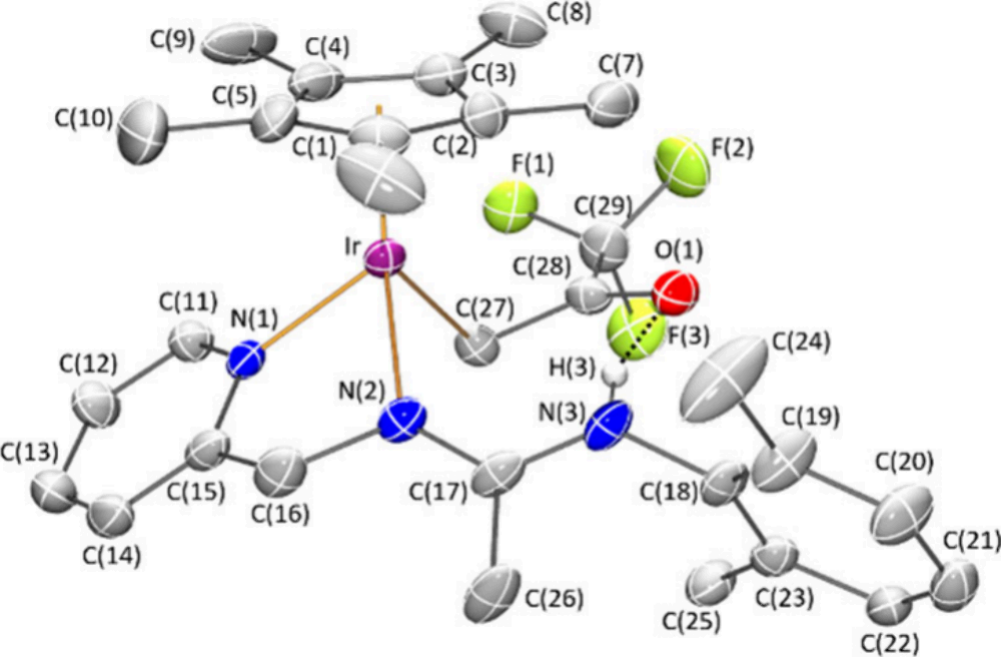
Molecular structure of the cation of complex **8***Z* (with 50% probability ellipsoids) showing the N(3)–H(3)···O(1) interaction. For clarity, hydrogen atoms (except the NH proton) have been omitted. Selected bond lengths (Å) and angles (deg): Ir–Ct 1.8097(1), Ir–N(1) 2.090(4), Ir–N(2) 2.110(4), Ir–C(27) 2.161(5), N(2)–C(17) 1.297(7), N(3)–C(17) 1.348(7), C(28)–O(1) 1.225(7); Ct–Ir–N(1) 127.64(14), Ct–Ir–N(2) 128.86(14), Ct–Ir–C(27) 134.55(17), N(1)–Ir–N(2) 77.04(17), N(1)–Ir–C(27) 82.24(17), N(2)–Ir–C(27) 86.75(17). Ct represents the centroid of the Cp* ring.

Crystals of **8***Z* suitable for an X-ray diffraction analysis were obtained by slow crystallization from CH_2_Cl_2_/diethyl ether solutions of **8***Z*/**8***E* mixtures of 10:90 molar ratio. An ORTEP view of the cation is depicted in Figure 1. Activation of one CH bond of the ketone CH_3_COCF_3_ is apparent: in the cation, an acetonyl CH_2_COCF_3_ moiety is coordinated to iridium through the methylene carbon, and the uncoordinated amidine nitrogen is protonated. The pyridinyl-amidine ligand adopts a chelate κ^2^*N*,*N′*coordination mode, and an η^5^-Cp* group completes the coordination sphere of the metal. A hydrogen bond between the N(3)–H(3) proton and the O(1) atom [N–H 0.86(2) Å, H···O 2.18(6) Å, N···O 3.001(7) Å, N–H···O 160(6)°] was observed.

**Scheme 2:**
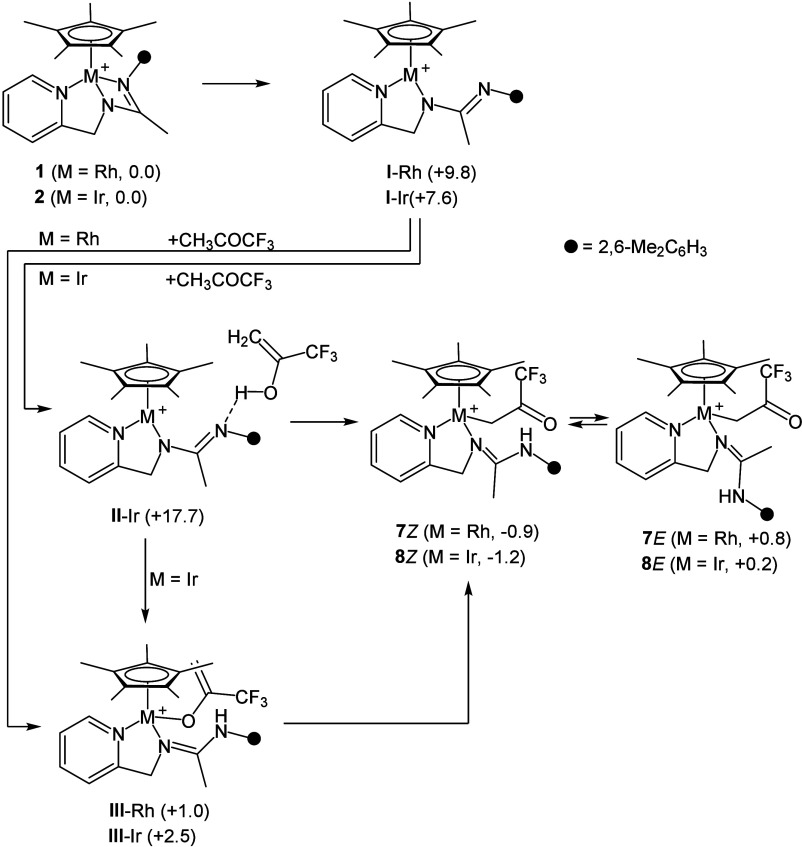
Reaction Sequence for **1** (or **2**) + CH_3_COCF_3_ → **7** (or **8**) Relative Gibbs free energy values are given in kcal·mol^–1^ (B97D3/def2svp).

DFT calculations were performed in order to gain insight into the course of the reactions leading to **3**–**8** as well as the equilibria observed in the resulting reaction mixture. According to the reaction sequence shown in Scheme 2, the first step is the endergonic formation of the FLP species **I**-Rh or **I**-Ir as a result of the metal–nitrogen bond dissociation from **1** or **2**, respectively.

As for the iridium derivative **I**-Ir, its reaction with the enolic form of the ketone CH_3_COCF_3_ leads to **II**-Ir that exhibits an intermolecular N···HO hydrogen bond between the basic site of the FLP **I**-Ir and the OH group of the incoming enol CH_2_=COH(CF_3_).

**Figure 2:**
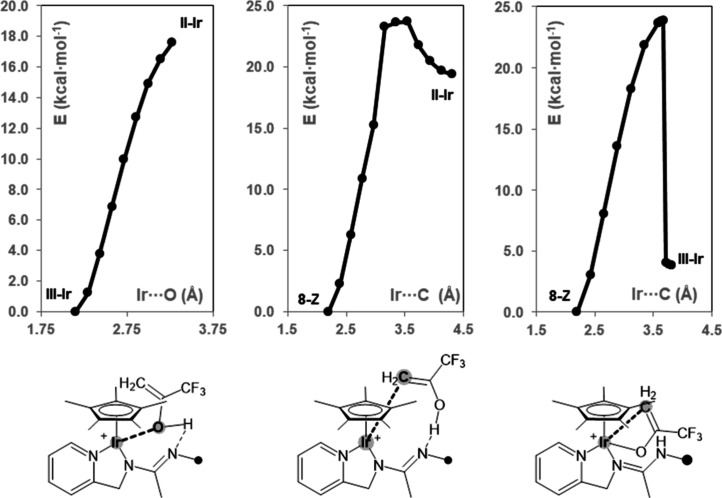
Energy profile for **II**-Ir → **III**-Ir (left), **II**-Ir → **8**Z (center), and **III**-Ir → **8***Z* (right) scanning the iridium···oxygen, iridium···carbon, and iridium···carbon coordinates, respectively (B97D3/def2svp; E, kcal·mol^–1^; Ir···O or Ir···C, Å).

Afterward, **II**-Ir should evolve to **III**-Ir as a result of the O–H activation rendering the metal–oxygen bond along with the N–H bond. This step was calculated to be barrierless (Figure 2, left). In its turn, **III**-Ir may finally isomerize to **8***Z* by means of a 1,3 migration of the metal center, reminiscent of a keto–enol tautomerism. In this regard, the transition state of the step **III**-Ir → **8***Z* could not be located on the potential energy surface. As a consequence, the activation barrier was estimated scanning the Ir···C coordinate, affording a putative transition state at +23.7 kcal·mol^–1^ with respect to **8***Z* (Figure 2, right). Alternatively, **8***Z* could be obtained straightforward from **II**-Ir as a result of the synchronous iridium–carbon and nitrogen–hydrogen bond formation and the oxygen–hydrogen bond splitting. Also in this case, the transition state could not be located on the potential energy surface, but a putative transition state at +23.7 kcal·mol^–1^ with respect to **8***Z* was encountered by scanning the Ir···C coordinate (Figure 2, center).

On the other hand, as far as the rhodium counterpart is concerned, the rhodium derivative **II**-Rh, structurally related to **II**-Ir, could not be located on the potential energy surface. Thus, in this case, **I**-Rh should directly transform into **III**-Rh upon reaction with the enol CH_2_=COH(CF_3_) and should eventually isomerize to **7***Z*. Similar to **III**-Ir, the formation of **III**-Rh from the putative **II**-Rh was calculated to be barrierless by scanning the rhodium···carbon coordinate, whereas a putative transition state at +21.3 kcal·mol^–1^ with respect to **7***Z* was calculated for **III**-Rh → **7***Z* by scanning the rhodium···carbon coordinate.

Once formed, both **7***Z* and **8***Z* should finally isomerize to **7***E* and **8***E*, respectively, by means of rotation of the amidine moiety around the C=N bond (Scheme 2). In this regard, as shown in related amidine or guanidine systems,^25^ the electronic delocalization along the nitrogen–carbon–nitrogen moiety might play a crucial role. For the sake of comparison, the relative stability of *Z* and *E* isomers for **3**–**6** was also calculated, and relative Gibbs free energy values (*G*_*E*_-*G*_*Z*_) in the range +0.8 – + 2.2 kcal·mol^–1^ [**3**, + 1.7; **4**, + 2.2; **5**, + 0.8; **6**, + 1.6 kcal·mol^–1^ (B97D3/def2tzvp//B97D3/def2svp)] were obtained in agreement with the observed presence of both *E* and *Z* isomers of **3**–**8** in the final reaction mixture.

### Reaction with Hexafluoroacetone and Trifluoromethyl Phenyl Ketone

Under conditions analogous to those outlined for the reaction with methyl ketones, complexes **1** and **2** do not exhibit reactivity toward ketones that lack α hydrogen, such as benzophenone, di-4-fluorophenyl ketone, di-4-chlorophenyl ketone, or 4-nitrophenyl phenyl ketone. Under harsher reaction conditions, complex mixtures of products form, which subsequently decompose. However, both complexes are capable of activating the C=O double bond in ketones lacking α hydrogen but bearing strong electron withdrawing substituents, such as hexafluoroacetone or trifluoromethyl phenyl ketone. Significant differences in reaction pathways become evident when comparing the behavior of the rhodium complex with that of its iridium counterpart. Hence, we will examine the reactivity of each of these two complexes with these two ketones separately, beginning with the iridium compound **2**.

#### Reactions with the Iridium Complex **2**

When a THF-*d*_8_ solution, containing complex **2** and 5 equiv of hexafluoroacetone trihydrate, is heated to 353 K overnight in the presence of 4 Å MS, adduct **9** readily forms.^27^ Trifluoromethyl phenyl ketone behaves in a similar manner. Specifically, when a solution, containing compound **2** in CF_3_COPh as the solvent, is heated at 423 K for 5 h, complex **10** is produced, as shown in eq 2. Formally, the process can be broken down into three main steps: (i) cleavage of the Ir–NR^1^ bond in complex **2**, (ii) insertion of the ketone C=O bond into one of the two methylene C–H bonds, and (iii) protonation of the newly unbound nitrogen atom and metal–oxygen bond formation.

The formation of iridium compounds **9** and **10** by the reaction of complex **2** with ketones bears some resemblance to the reaction of cyclopentanone with the ruthenium pincer [Ru(PNN)H(CO)] (PNN = 6-(di-*tert*-butylphosphinomethylene)-2-(*N*,*N*-diethyl aminomethyl)-1,6-dihydropyridine). Similar to complex **2**, the ruthenium pincer adds to the C=O bond of the cyclopentanone, resulting in the formation of C–C and Ru–O bonds.^13^
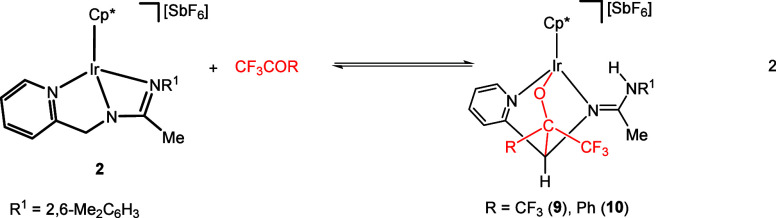
2

It should be noted that in complexes **9** and **10** the metal and the methinic carbon are stereogenic centers. Furthermore, in complex **10** (R = Ph) the carbonyl carbon also becomes stereogenic. Additionally, *Z*/*E* isomers around the C=N double bound can also be obtained. Therefore, several stereoisomers of these adducts could form. Remarkably, formation of compound **9** is highly diastereoselective: only two isomers in about a 99:1 molar ratio have been detected by NMR spectroscopy. Regarding compound **10**, it was isolated as a mixture of four isomers in a molar ratio of about 92:4:3:1.

NMR measurements in solution support the structure proposed in eq 2 and provide important additional structural information. Focusing the discussion on the major isomer of compound **10**, in the ^1^H NMR spectrum, a singlet at 5.94 ppm is observed that replaces the AB system ascribed to the methylene protons of the pyridinyl-amidinato ligand in the starting compound **2**.^25a^ The ^13^C{^1^H} NMR spectrum shows two quartets: one centered at 127.15 ppm (^1^*J*(FC) = 293.1 Hz) assigned to the CF_3_ carbon and another at 81.49 ppm (^2^*J*(FC) = 26.0 Hz) attributed to the C–O carbon nucleus. The ^19^F NMR spectrum consists of a singlet at −72.96 ppm. An IR band at 1157 cm^–1^ can be assigned to the stretching of a CO single bond. All these spectroscopic data together denote the presence of a CF_3_COCH moiety in the molecule. Additionally, a singlet at 6.95 ppm in the ^1^H NMR spectrum and a broad IR band centered at 2969 cm^–1^ indicate the presence of an NH functionality.

Similarly, the NMR and IR spectra of the major isomer of complex **9** evidence the presence of (CF_3_)_2_COCH and NH fragments arising from the addition of the complex **2** to the ketone (CF_3_)_2_CO (see Experimental Section).

On the other hand, in ^1^H NOE experiments, irradiation of the amidine methyl or the NH protons of the major isomer enhances the methinic or the Cp* resonances, respectively, in both cases (see Figures S24 and S25). Therefore, we propose that, in these isomers, the configuration around the C=N double bond is *Z*.

**Figure 3:**
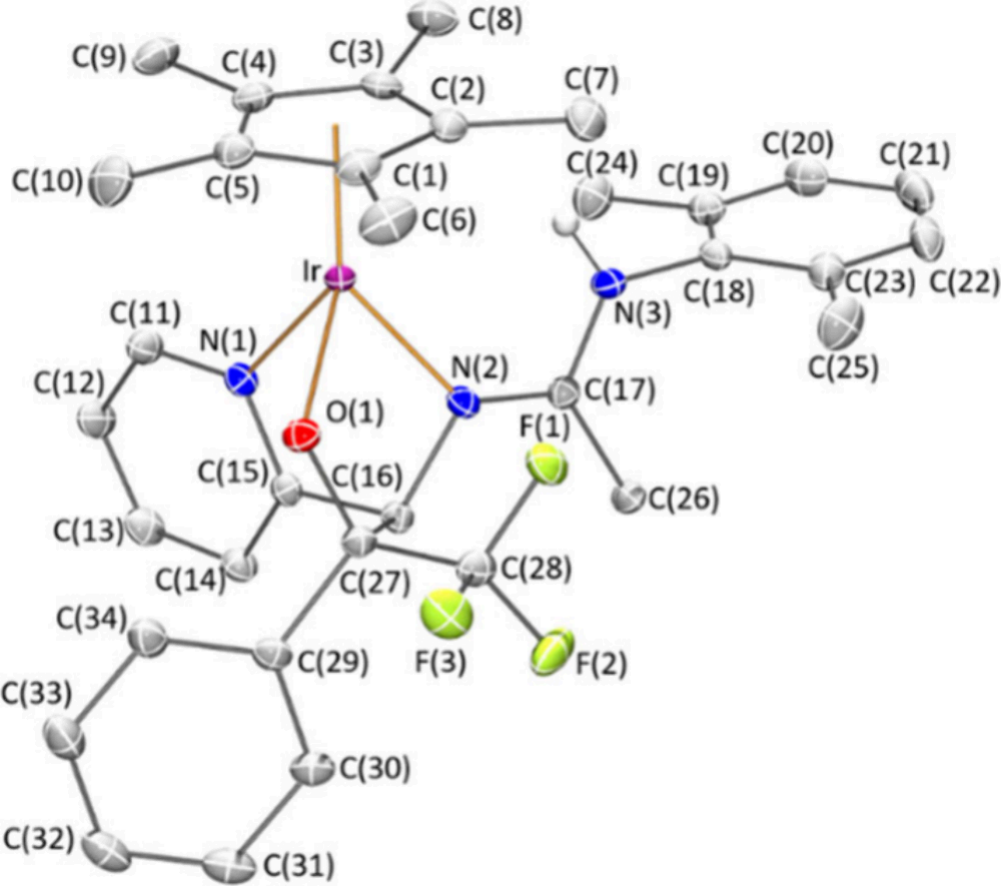
Molecular structure of the cation of the major isomer of complex **10** (with 50% probability ellipsoids). For clarity, hydrogen atoms (except the NH proton) have been omitted. Selected bond lengths (Å) and angles (deg): Ir–Ct 1.7907(1), Ir–O(1) 2.081(2), Ir–N(1) 2.124(2), Ir–N(2) 2.109(3), N(2)–C(17) 1.301(4), N(3)–C(17) 1.344(4); Ct–Ir–O(1) 121.92(7), Ct–Ir–N(1) 137.77(9), Ct–Ir–N(2) 140.56(8), O(1)–Ir–N(1) 79.77(9), O(1)–Ir(1)–N(2) 78.89(9), N(1)–Ir–N(2) 74.13(10). Ct represents the centroid of the Cp* ring.

Crystals suitable for an X-ray diffraction analysis were obtained by slow crystallization from concentrated CH_2_Cl_2_ solutions of the isomeric mixture obtained for complex **10**. In the cation (Figure 3), the pyridinyl-amidine ligand is linked to the incoming ketone via a newly formed C–C bond, thus giving a pyridine-alkoxide-imine-amine ligand which adopts a *fac* κ^3^*O*,*N*,*N′* arrangement involving the pyridine and imine nitrogen atoms. An η^5^-Cp* group completes the coordination sphere of the metal. The absolute configuration of the cation in the measured crystal is *S*_Ir_,*R*_C(16)_,*R*_C(27)_.^28^ Since the crystals belong to the chiral space group *P*1 of the triclinic system, a conglomerate^29^ of the *S*_Ir_,*R*_C(16)_,*R*_C(27)_ and *R*_Ir_,*S*_C(16)_,*S*_C(27)_ enantiomers of compound **10** was isolated. The structure determined by X-ray diffraction analysis corresponds to the major diastereomer detected in solution because its NMR spectra coincide with those of the isolated crystals.

**Scheme 3:**
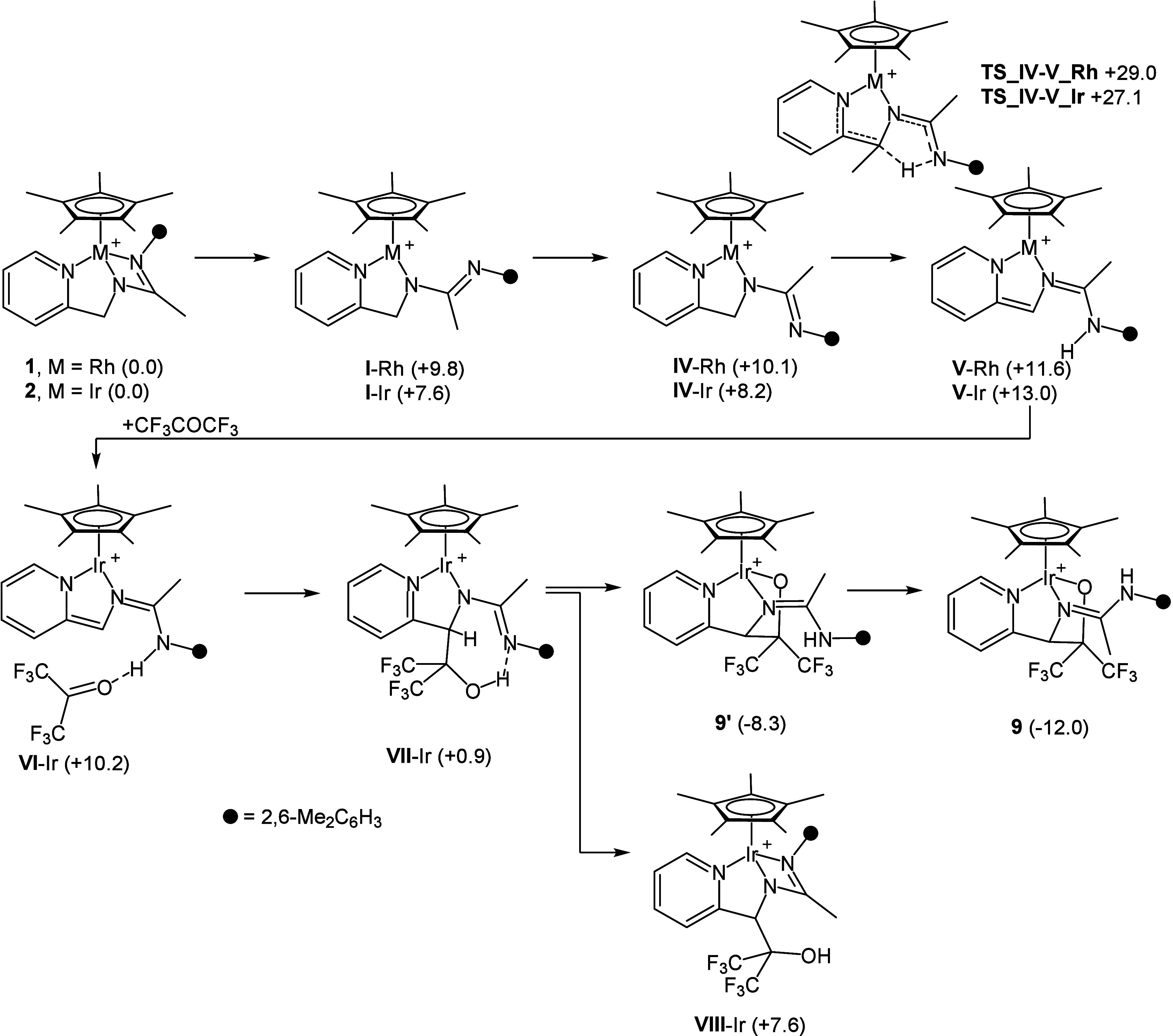
Reaction Sequence for **2** + CF_3_COCF_3_ → **9** Relative Gibbs free energy values are given in kcal·mol^–1^ (B97D3/def2svp).

The mechanism of the formation of **9** and **10** was elucidated by DFT calculations (Scheme 3). En route to **9**, the first step is the iridium–nitrogen bond dissociation leading to the FLP **I**-Ir, which subsequently gives place to the rotamer **IV**-Ir. The subsequent intramolecular 1,4 hydrogen shift in **IV**-Ir eventually renders the intermediate **V**-Ir via the transition state **TS_IV–V_Ir** (+27.1 kcal·mol^–1^). The reaction of **V**-Ir with CF_3_COCF_3_ affords the hydrogen bond adduct **VI**-Ir, which undergoes the carbon–carbon bond formation along with the hydrogen migration from nitrogen to oxygen affording **VII**-Ir, still exhibiting an intramolecular OH···N hydrogen bond. As for the transformation **VI**-Ir → **VII**-Ir, the transition state could not be located on the potential energy surface. Nonetheless, the scan of the carbon···carbon coordinate indicated that the process should be substantially barrierless (see Figure S30, left). Finally, the deprotonation of the OH group of **VII**-Ir by the imine group and the coordination of the oxygen atom to iridium affords **9***′*. In its turn, **9***′* isomerizes to the most stable isomer **9** by means of the rotation of the =CCH_3_NH(2,6-Me_2_C_6_H_3_) moiety around the N=C bond. The barrier for this rotation was estimated to be Δ*E*_act_ +16.5 kcal·mol^–1^ by scanning the C–N=C–N coordinate (see Figure S30, right), thus it should be easily accessible under the experimental conditions. Notably, the calculated relative stability of **9** (−12.0 kcal·mol^–1^) vs **9***′* (−8.3 kcal·mol^–1^) nicely fit in with the observed molar ratio between **9** and **9**′.

Finally, for the sake of comparison, the reaction **VII**-Ir →**VIII**-Ir was calculated to be highly endergonic, thus ruling out the formation of the κ^3^*N*,*N*′,*N″* complex structurally related to the starting compound **2**.

As far as the reaction of the rhodium complex **1** with CF_3_COCF_3_ is concerned, the transition state **TS_IV–V_Rh** was calculated to lie at +29.0 kcal·mol^–1^, that is, a higher energy barrier exists for **1** when compared to **2**, which is reasonably the cause why the formation of the putative rhodium counterpart of **9** and **9**′ was not observed.

**Scheme 4:**
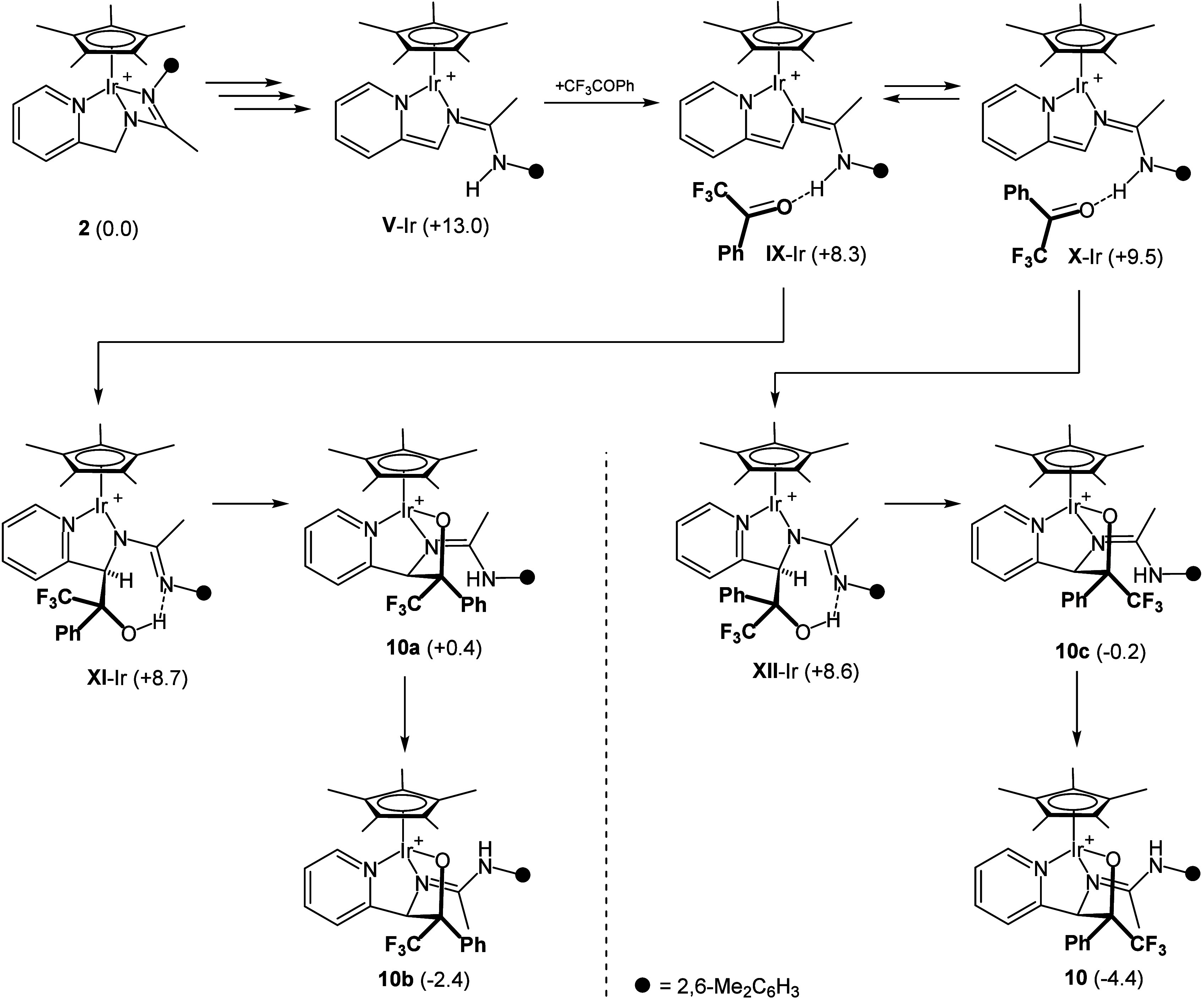
Reaction Sequence for **2** + CF_3_COPh → **10** Relative Gibbs free energy values are given in kcal·mol^–1^ (B97D3/def2svp).

As mentioned before, when it comes to the reaction of **2** with CF_3_COPh, the diastereoselectivity of the reaction is a relevant issue to be taken into account. Scheme 4 shows the reaction sequence leading to the four diastereomers **10**, **10a**, **10b**, and **10c**.

Similar to the reaction of **2** with CF_3_COCF_3_, **V**-Ir is the key intermediate which is able to form a hydrogen bond adduct with the incoming ketone CF_3_COPh. Actually, in this case, the two adducts **IX**-Ir and **X**-Ir of similar stability form depending on which enantioface of CF_3_COPh looks toward the complex. Thereafter, **IX**-Ir gives the diastereomer **XI**-Ir, whereas **X**-Ir gives place to the diastereomer **XII**-Ir. Notably, even though the transition states for neither **IX**-Ir → **XI**-Ir nor **X**-Ir → **XII**-Ir could be located on the potential energy surface, the activation barrier for each step was estimated by scanning the carbon–carbon coordinate (see Figure S31). Notably, the activation barriers were found to be similar (**IX**-Ir → **XI**-Ir Δ*E*_act_ = +7.2 vs **IX**-Ir; **X**-Ir → **XII**-Ir Δ*E*_act_ = +6.8 kcal·mol^–1^ vs **X**-Ir), which, along with the similar stability of **XI**-Ir and **XII**-Ir, reasonably rules out any sort of kinetic or thermodynamic control over the diasereoselectivity of the carbon–carbon bond formation. Finally, similar to **VII**-Ir, the deprotonation of the OH group of **XI**-Ir or **XII**-Ir by the imine group and the coordination of the oxygen atom to iridium afford **10a** and **10c**, respectively, which in their turn isomerize to the relatively more stable isomer **10b** and **10** by means of the rotation of the =CCH_3_NH(2,6-Me_2_C_6_H_3_) moiety around the N=C bond. Finally, it is worth a mention that the calculated relative Gibbs free energy values (kcal·mol^–1^) of **10a** (+0.4), **10b** (−2.4), **10c** (−0.2), and **10** (−4.4) nicely agree with the observed diastereomeric ratio given before.

#### Reactions with the Rhodium Complex **1**

When a solution of the rhodium complex [Cp*Rh(κ^3^*N,N*′*,N″*-**L**)][SbF_6_] (**1**) in dichloromethane was treated with 1.5 equiv of (CF_3_)_2_CO·3H_2_O and the resulting mixture was heated at 323 K for 48 h, in the presence of molecular sieves, a complete conversion to complex **11** was achieved.^27^ Alternatively, when the same rhodium complex, dissolved in CF_3_COPh, was heated at 333 K for 1 h, complex **12** was isolated in high yield (eq 3).

The most remarkable feature of the proton spectrum of the new compounds is the splitting of the 15-proton singlet of the Cp* group of the starting complex into five signals, four singlets, assigned to four inequivalent methyl groups, and one AB system adding up 14 hydrogen atoms. Likely, the missing Cp* hydrogen atom generates the NH functionality detected in the products. ^1^H, ^13^C, and ^19^F NMR and IR spectroscopic data together with microanalysis and mass spectrometry measurements (see Experimental Section for full details) support the schematic structure depicted in eq 3 for compounds **11** and **12**.

In particular, NOESY NMR analyses for these complexes show cross-peaks consistent with a *Z* configuration around the C=N double bond of the pyridinyl-amidine ligand in both cases (see Figures S26 and S27). Small amounts of other metal species were detected in the NMR spectra of complex **12**.
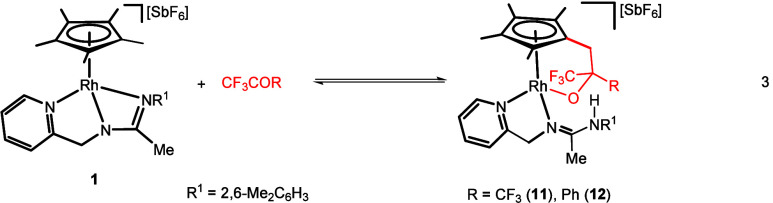
3

**Figure 4:**
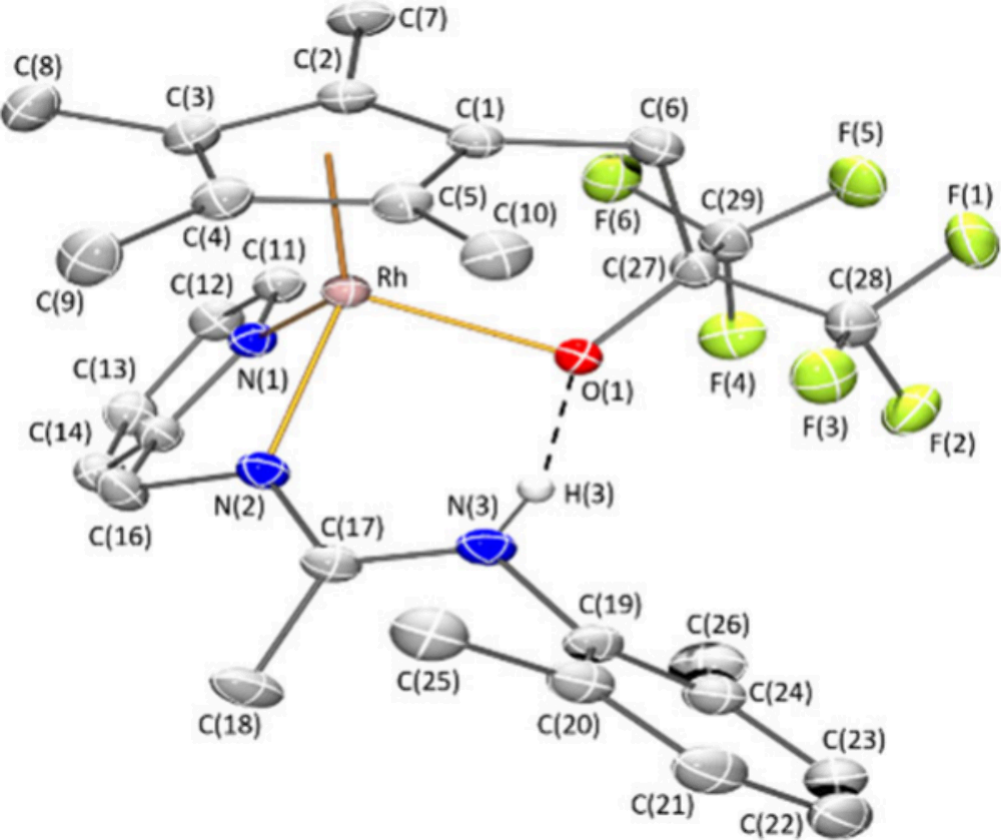
Molecular structure of the cation of complex **11** (with 50% probability ellipsoids). For clarity, hydrogen atoms have been omitted, except the NH proton. Selected bond lengths (Å) and angles Rh–Ct 1.7525(7), Rh–O(1) 2.0934(10), Rh–N(1) 2.1166(13), Rh–N(2) 2.1077(12), N(2)–C(17) 1.302(2), N(3)–C(17) 1.346(2); Ct–Rh–O(1) 117.19(4), Ct–Rh–N(1) 131.40(5), Ct–Rh–N(2) 132.87(5), O(1)–Rh–N(1) 96.61(5), O(1)–Rh–N(2) 89.98(5), N(1)–Rh–N(2) 76.32(5). Ct represents the centroid of the Cp ring.

To confirm the proposed structure, the crystal structure of compound **11** was determined by X-ray diffraction analysis (Figure 4). In sharp contrast to the behavior found for the iridium complex **2**, in the rhodium adduct **11** the Cp* ligand is C–C coupled with the ketone carbonyl carbon to give an alkoxide-substituted Cp ligand that tethers the metal through its alkoxide oxygen atom. The two remaining coordination sites are occupied by the pyridinyl-amidine ligand adopting a κ^2^*N*,*N*′ coordination mode.

The *Z* configuration of the C=N double bond of the pyridinyl-amidine ligand allows for the establishment of a N(3)–H(3)···O(1) hydrogen bond interaction within the cation: N(3)–H(3) 0.82(2) Å, H(3)···O(1) 2.05(3) Å, N(3)···O(1) 2.820(2) Å, N(3)–H(3)···O(1) 156(2)°.

The formation of complexes **11** and **12** from **1** represents another instance of reactivity of the Cp* ligand. Although examples of protonation, deprotonation, migration, or hydride abstraction reactions affecting this ligand are currently known,^25a,30^ its extensive use in organometallic chemistry is primarily attributed to the steric and electronic stability it imparts to its compounds, as well as its robustness as a spectator ligand.^31^

The ^1^H and ^19^F NMR spectra of dichloromethane solutions of compound **11** remain unchanged for hours. However, when a more polar solvent like THF is employed, they show the immediate formation of a new compound, which exists in equilibrium with the starting complex **11** (see SI). As the most representative NMR features, we observed in the ^1^H NMR spectrum of the new compound, at 273 K, a singlet at 7.56 ppm, which we attributed to an OH group, along with two new AB systems [δ 4.73, 4.62 ppm, *J* = 17.6 Hz; δ 2.96, 2.89 ppm, *J* = 15.4 Hz, (see Figure S22)]. Concurrently, the ^19^F NMR spectrum shows two new overlapping quartets at approximately −77.3 ppm that we assign to two diastereotopic CF_3_ groups (see Figure S23). Taking into account all these spectroscopic data, we tentatively propose that the tethered alkoxide ligand in **11** has evolved to the dangling alcohol substituent in **11a** through protonation of the alkoxide oxygen by the NH proton and that the NR^1^ nitrogen has recoordinated the metal center (eq 4).
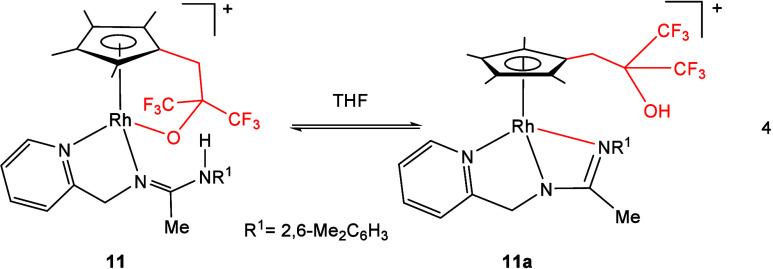
4

On the other hand, at 333 K, solutions of **12** in CF_3_COPh as the solvent evolve over time. NMR spectra recorded over 2–3 h show the formation of an ample variety of compounds and the mass spectra display peaks corresponding to molecular ions of *m*/*z* ratios of **1**·CF_3_COPh (**12**), **1**·2 CF_3_COPh (**14**), and **1**·3 CF_3_COPh (**16**). Changes over time of the relative intensity of these peaks indicate the successive and progressive formation of the new species with a higher number of added ketone molecules.

A similar behavior was observed in the reaction of **1** with hexafluoroacetone: after treating overnight complex **1** with 10 equiv of hexafluoroacetone trihydrate, in THF at 333 K, the mass spectrum shows peaks corresponding to **1**·(CF_3_)_2_CO (**11**), **1**·2(CF_3_)_2_CO, (**13**) **1**·3(CF_3_)_2_CO (**15**), and even **1**·4(CF_3_)_2_CO (**17**) (see Figures S28 and S29).

**Figure 5:**
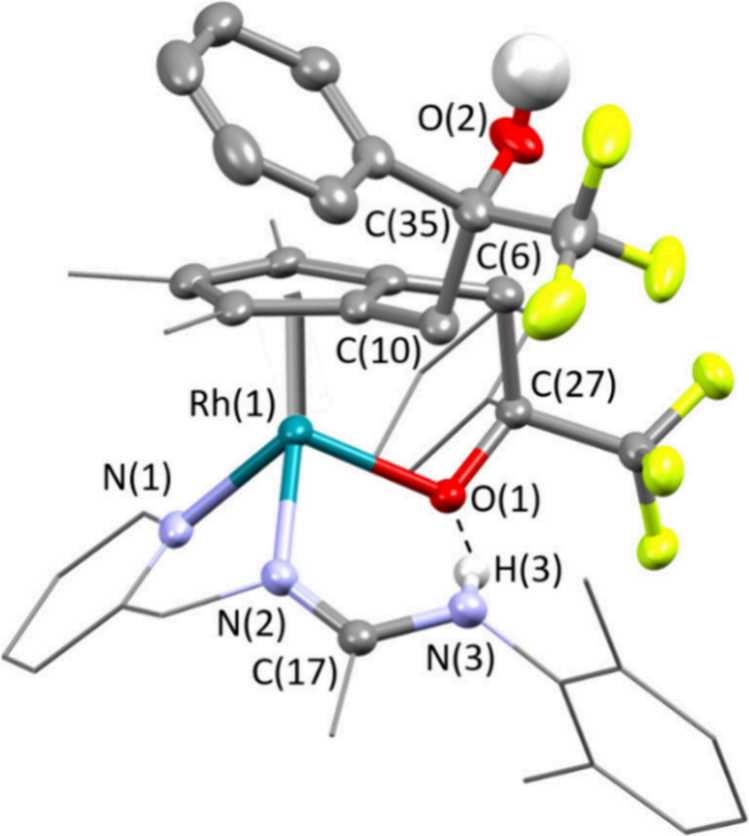
Schematic representation of cation of complex **14**. For simplicity, only selected atoms have been represented with 50% probability ellipsoids. Selected bond lengths (Å) and angles (deg): Rh(1)–Ct 1.7479(12), Rh(1)–O(1) 2.1041(19), Rh(1)–N(1) 2.105(2), Rh(1)–N(2) 2.094(3), N(2)–C(17) 1.299(4), N(3)–C(17) 1.353(4), C(27)–O(1) 1.397(3), C(6)–C(27) 1.573(4), C(35)–O(2) 1.421(5), C(10)–C(35) 1.559(4); Ct–Rh(1)–O(1) 117.84(7), Ct–Rh(1)–N(1) 129.07(8), Ct–Rh(1)–N(2) 132.03(8), O(1)–Rh(1)–N(1) 100.91(8), O(1)–Rh(1)–N(2) 88.85(8), N(1)–Rh(1)–N(2) 76.33(10). Ct represents the centroid of the Cp* ring.

Suitable crystals for X-ray diffraction analysis were obtained through the slow diffusion of a diethyl ether/*n*-hexane mixture into solutions of compound **1** containing an excess of CF_3_COPh that had been held at 333 K for 2 h. The crystal structure consists of SbF_6_ anions and cations resulting from the addition of two molecules of ketone to complex **1** (Figure 5). Within the cation, the metal coordination sphere is comparable to that found in complex **11**. As a difference, the Cp ring of **14** exhibits a −CH_2_–C(Ph)(CF_3_)(OH) substituent that can be considered the result of the protonation of the tethered alkoxide ligand present in the cation of the initial complex **12**. Formally, the substituted Cp ligand results from the addition of one C(sp^3^)–H bond of one of the CH_3_ groups of the original Cp* ligand to the C=O double bond of one ketone molecule. As in the molecular structure of compound **11**, the second ketone molecule gives place to an alkoxide link between the Cp ring and the rhodium atom. The two Cp* methyl groups added to the ketones are adjacent. Finally, the uncoordinated NR^1^ nitrogen of the pyridinyl-amidinato ligand is protonated.

Hydrogen bond interactions between N(3)–H(3) and O(1) of the cation [N(3)–H(3) 0.82(3) Å, H(3)···O(1) 1.88(4) Å, N(3)···O(1) 2.688(3) Å, N(3)–H(3)···O(1) 167(3)°] and between the OH group and a fluorine atom of the SbF_6_ anion [O(2)–H(2) 0.88(5) Å, H(2)···F(8) 1.93(5), N(3)···F(8) 2.804(4), O(2)–H(2)···F(8) 169(6)°] have been detected.

According to the priority rules,^28^ the absolute configuration of the cation is *R*_Rh_,*R*_C(27)_,*R*_C(35)_,*R*_Pl_, and as the compound crystallizes in the achiral monoclinic space group *P*2_1_/*c*, both the *R*_Rh_,*R*_C(27)_,*R*_C(35)_,*R*_Pl_ and *S*_Rh_,*S*_C(27)_,*S*_C(35)_,*S*_Pl_ enantiomers of the complex are present in the crystal.

**Scheme 5:**
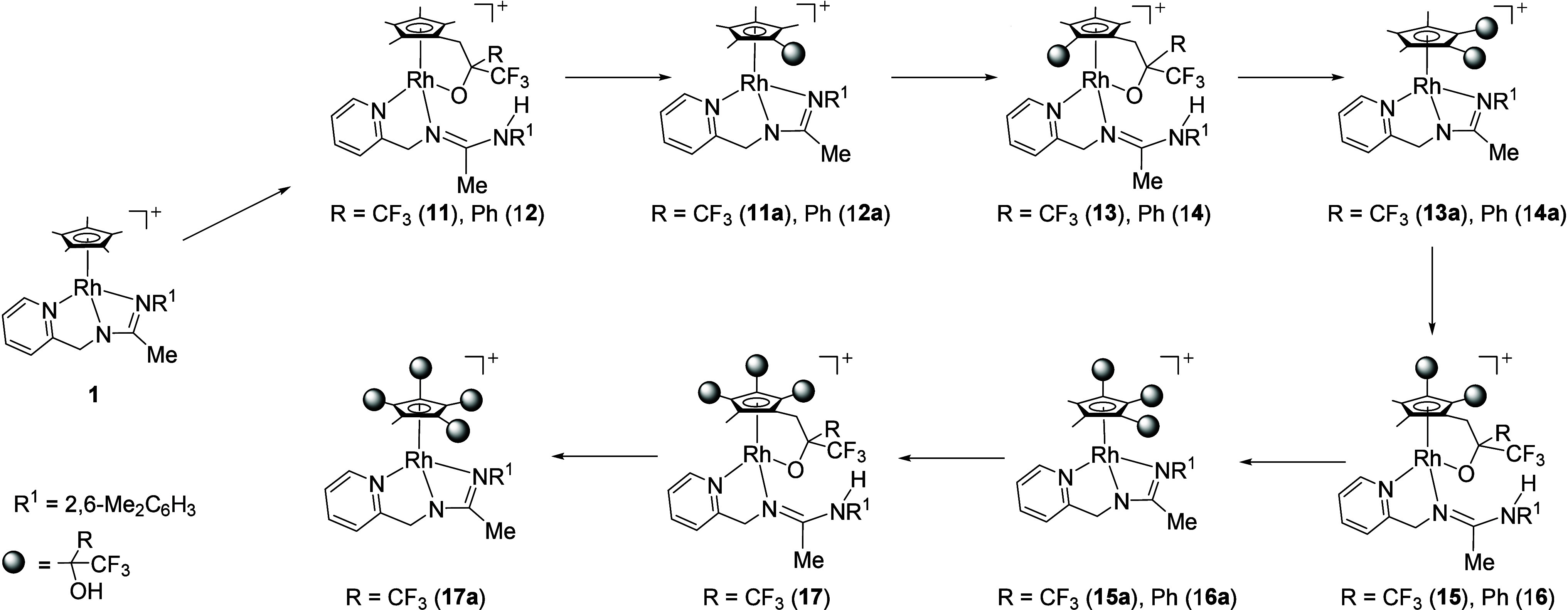
Proposed Evolution of the Reaction of Complex **1** with Hexafluoroacetone or Phenyl Trifluoromethyl Ketone For clarity, not all possible stereoisomers are shown.

NMR characterization of the new compounds in the reaction mixture is unattainable. The high complexity of the NMR spectra arises, on the one hand, from the high number of different species present in the solution, denoted by the number of molecular ions detected by mass spectrometry and, on the other, from the high number of possible stereoisomers for each new stoichiometry. It must be taken into account that, apart from *E*/*Z* isomerism around the C=N bond of the pyridinyl-amidine ligand, di-, tri-, and tetra-substitution at the Cp ring also generates distinct planar stereoisomers. Moreover, the rhodium atom, in all the reaction adducts, and the carbonyl carbon atom, in the trifluoromethyl phenyl ketone derivatives, are stereogenic centers thus enabling the formation of different diastereomers. In summary, NMR assignment is unaffordable. However, the structural characterization of compounds **11** and **14** by X-ray diffraction means, along with the spectroscopic determination of the equilibrium **11** ⇌ **11a** (tethered alkoxide ⇌ dangling alcohol, eq 4), tentatively allows us to suggest the evolution from **1** to **16** or **17** as illustrated in Scheme 5. The C–C coupling of a methyl group from Cp* with the carbonyl carbon of a ketone molecule results in an alkoxide which tethers the Cp ring to the rhodium atom and in the protonation of the resulting uncoordinated nitrogen atom (complexes **11** and **12**). Subsequent attack of the alkoxide oxygen to the generated NH proton gives an alcohol substituent on the Cp ring, and the deprotonated nitrogen subsequently recoordinates to the rhodium (complexes **11a** and **12a**). This sequence takes place iteratively up to three (R = Ph) or four (R = CF_3_) times, eventually resulting in the formation of **16** and **17**. DFT calculations (*vide infra*) provide support for this hypothesis.

**Scheme 6:**
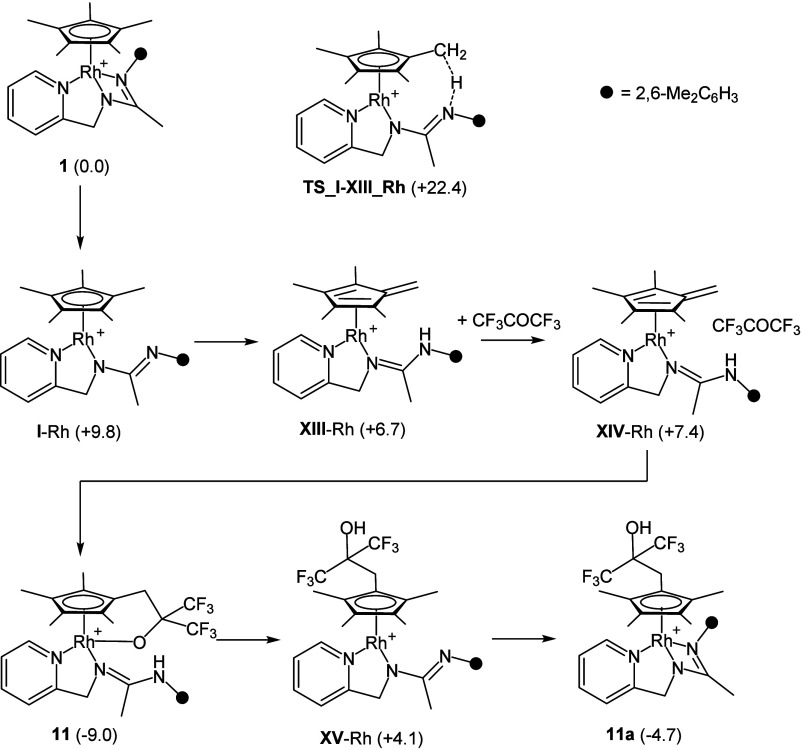
Reaction Sequence for **1** + CF_3_COCF_3_ → **11** Relative Gibbs free energy values are given in kcal·mol^–1^ (B97D3/def2svp).

The steps leading to the formation of **11** were elucidated by means of DFT calculations. Scheme 6 shows the reaction sequence along with the relative Gibbs free energy values. As mentioned before, the dissociation of the rhodium–nitrogen bond in **1** affords the FLP species **I**-Rh, which eventually undergoes an intramolecular hydrogen abstraction for the Cp* ligand rendering the fulvene derivative **XIII**-Rh via the transition state **TS_I-XIII_**Rh (+22.4 kcal·mol^–1^). Thereafter, **XIII**-Rh reacts with CF_3_COCF_3_ affording the adduct **XIV**-Rh which evolves to **11** through a barrierless process (see Figure S32). Notably, in agreement with observed composition of the equilibrium reaction mixture (*vide supra*), **11** could equilibrate with **11a** via the sequence **11** → **XV**-Rh → **11a**, **XV**-Rh being thermally accessible under the experimental conditions, even though it should be present in amounts well below the detection limits of NMR spectroscopy.

**Scheme 7:**
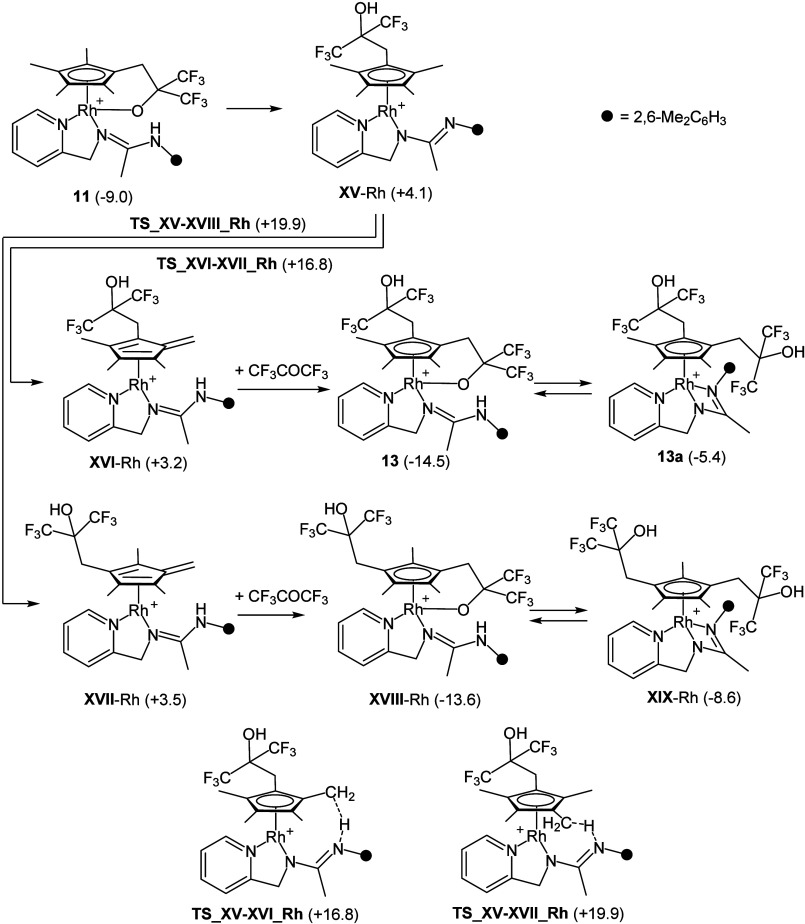
Reaction Sequence for **11** + CF_3_COCF_3_**→13** along with Relative Gibbs Free Energy Values (kcal·mol^–1^) of Intermediates and Transition States (B97D3/def2svp)

As far as the formation of disubstituted cyclopentadienyl rhodium derivatives is concerned, Scheme 7 shows the calculated routes leading to **13** and its isomer **13a**, **XVIII**-Rh, and **XIX**-Rh. Notably, the formation of **13** (1,2-disubstituted) is kinetically (ΔΔ*G*_act_ = −3.1 kcal·mol^–1^) and thermodynamically (ΔΔ*G*_r_ = −0.9 kcal·mol^–1^) favored over the formation of **XVIII**-Rh (1,3-disubstituted). On the other hand, similar to what observed for **11** and **11a**, the tethered isomer **13** is more stable than the dangling isomer **13a**.

Finally, for the sake of comparison, the formation of the tethered isomer **17** was also calculated to be exergonic (**1** + 4 CF_3_COCF_3_ → **17**, Δ*G*_r_ = −11.7 kcal·mol^–1^), the dangling isomer **17a** being 5.9 kcal·mol^–1^ less stable than **17**.

### On the Origin of Selectivity

**Scheme 8:**
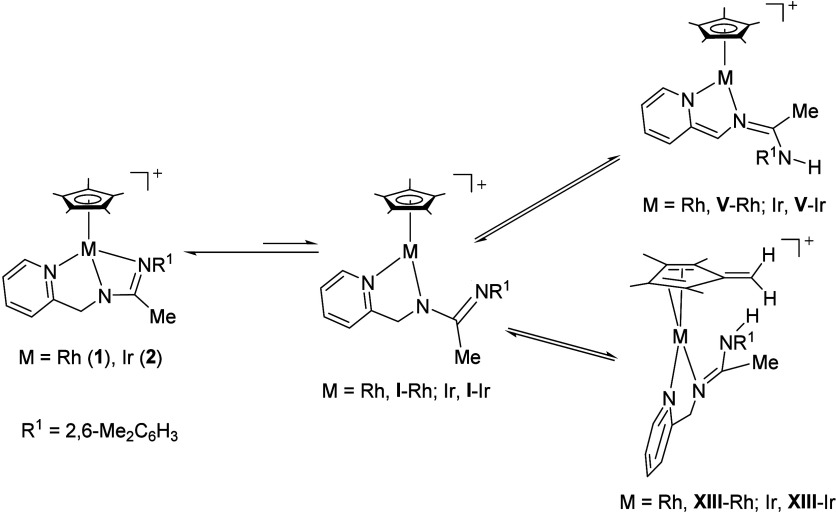
Formation of the Active Species **I**, **V**, and **XIII** from **1** and **2**

Compounds **1** and **2** are stable half-sandwich complexes whose spectroscopic features remain essentially unchanged for days, whether in the solid state or in usual organic solvents. They can be prepared in gram-scale quantities, can be stored for extended periods, and, therefore, are ideal starting materials for the examination of their reactivity. Remarkably, they exhibit the intriguing characteristic that, when in solution, several highly reactive species are easily accessible from them. Indeed, when the relief of strain associated with the opening of their M–N–C–N four-membered metalacycle, under the experimental conditions usually employed, is taken advantage of, compounds **1** and **2** are in equilibrium with **I**-Rh and **I**-Ir, thus behaving as frustrated Lewis pairs^26^ (Scheme 8). Furthermore, the resulting nucleophilic uncoordinated nitrogen atom in **I**-Rh and **I**-Ir is capable of promoting a prototropic tautomeric rearrangement involving the dearomatization of the pyridine ring and resulting in the formation of **V**-Rh and **V**-Ir, respectively. Alternatively, the uncoordinated nitrogen atom in **I**-Rh and **I**-Ir facilitates the abstraction of one proton from the Cp* ligand giving rise to the fulvene complexes **XIII**-Rh or the iridium counterpart **XIII**-Ir (*vide infra*).

Species **I**, **V**, and **XIII** have shown to be well-suited candidates for the development of metal–ligand cooperative reactivity patterns. Indeed, the C–H bond in methyl ketones is formally activated by type **I** intermediates of both metals, rhodium and iridium, leading to the formation of acetonyl compounds through collaborative FLP mechanisms (eq 1, Scheme 2). On the other hand, intermediates **V** and **XIII** can add to the C=O bond of nonenolizable ketones with electron-withdrawing substituents, such as (CF_3_)_2_CO or CF_3_COPh. The observed formation of rhodium derivatives **11** and eventually **13** seems to rely on the easily accessible intermediate **XIII**-Rh through **TS_I**-**XIII-Rh** (+22.4 kcal·mol^–1^) whereas **V**-Rh (+11.6 kcal·mol^–1^) should not form because of the comparatively high calculated activation barrier (**TS_IV**-**V_Rh**, +29.0 kcal·mol^–1^), thus preventing the formation of the rhodium counterpart of **9** and **9′**.

**Scheme 9:**
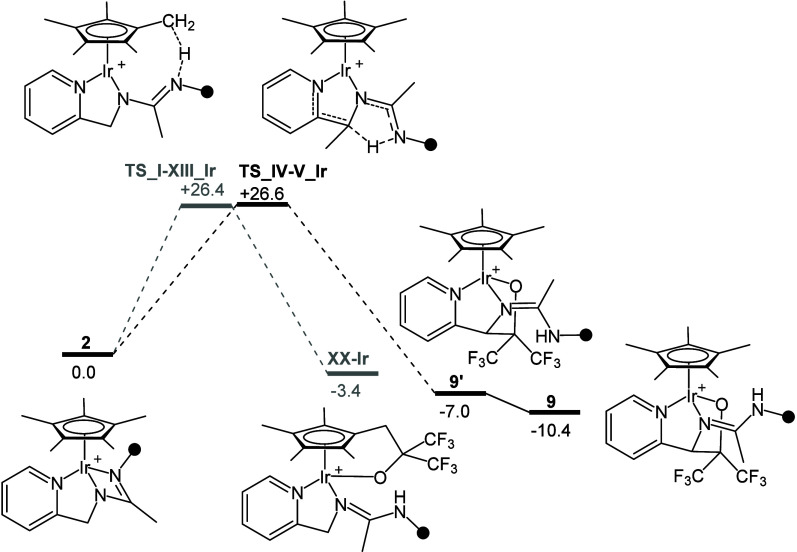
Simplified Gibbs Free Energy Profiles for the Putative Reaction **2** → **XX**-Ir and for **2** → **9′** → **9** (M06/deftzvp//B97D3/def2svp)

On the other hand, when it comes to the reactivity of **2** with nonenolizable ketone (CF_3_)_2_CO, Scheme 9 shows the comparison of the simplified Gibbs free energy profiles for the reactions **2** → **9′** → **9**, calculated at the level M06/deftzvp//B97D3/def2svp. The calculated activation barriers for both processes are similar (**TS_I-XIII_Ir**, +26.4; **TS_IV–V_Ir**, +26.6 kcal·mol^–1^), but the relative Gibbs free energy values indicate that **XX**-Ir (−3.4 kcal·mol^–1^) is less stable than **9**′ (−7.0 kcal·mol^–1^) and **9** (−10.4 kcal· mol^–1^), thus suggesting that under experimental conditions, only the formation of **9** and **9′** is observed as a consequence of their higher stability along with the reversibility of the formation of **XX**-Ir.

## Conclusions

The study of the reactivity of simple ketones with the semisandwich compounds [Cp*M(κ^3^*N*,*N*′,*N″*-**L**)][SbF_6_] (M = Rh, **1**; Ir, **2**) has revealed some peculiarities. Three modes of ketone activation have been found, all based on metal–ligand cooperation reactivity models. The C(sp^3^)–H bond of methyl ketones CH_3_COR (R = CH_3_, Ph, CF_3_) is activated by the two metal complexes following FLP-type reaction pathways. In this case, the ketonic C=O bond is not affected. The presence of electron-withdrawing substituents on nonenolizable ketones is necessary to observe addition reactions to this double bond. Thus, both the rhodium complex and the iridium complex add to the carbonyl group of (CF_3_)_2_CO and CF_3_COPh. However, while the addition of **1** occurs through the rhodium atom and the carbon of an exocyclic methylene of the Cp* ligand of a fulvene intermediate, the addition of **2** takes place through the metal and an exocyclic carbon of the dearomatized pyridinyl ring of the nitrogen ligand.

A remarkable feature of the study conducted is that a single organometallic structure, that is the dormant FLP, is able to develop the three metal–ligand cooperative patterns of activation, the ketone and the metal center being decisive in which one of them is exclusively operational. A plausible explanation for this behavior is obtained through experimental and theoretical studies, in which the intermediates **I**, **V**, and **XIII** derived from compounds **1** and **2** (*vide supra*) play a fundamental role.

It should be noted that all the reported activation processes take place with total atom economy, no external bases are required as preactivation reagents and, therefore, no residues form.

In summary, the stereoelectronic characteristics of intermediates **I**, **V**, and **XIII** enable them to develop a rich and sustainable metal–ligand cooperative chemistry, which in this work is applied to the activation of simple ketones. These results pave the way for studying the activation of other substrates as well as their extension to related catalytic processes. These studies are currently in progress in our laboratory.

## Experimental Section

### General Information

All preparations have been carried out under argon, unless otherwise stated. All solvents were treated in a PS-400-6 Innovative Technologies Solvent Purification System (SPS). Infrared spectra were recorded on a PerkinElmer Spectrum-100 FT-IR spectrometer (ATR mode). Carbon, hydrogen, and nitrogen analyses were performed using a PerkinElmer 240 B microanalyzer. ^1^H, ^19^F, and ^13^C NMR spectra were recorded on a Bruker AV-300 (300.13 MHz), a Bruker AV-400 (400.16 MHz), or a Bruker AV-500 (500.13 MHz) spectrometers. Chemical shifts are expressed in parts per million upfield from SiMe_4_ (^1^H and ^13^C) and CFCl_3_ (^19^F). *J* values are given in Hz. COSY, NOESY, HSQC, HMQC, and HMBC ^1^H–X (X = ^1^H, ^13^C) correlation spectra were obtained using standard procedures. Mass spectra were obtained with a Micro Tof-Q Bruker Daltonics spectrometer.

### Reaction of the Complexes **1** and **2** with CH_3_COR

In an NMR tube, complexes [Cp*M(κ^3^*N,N*′*,N″*-**L**)][SbF_6_] (M = Rh, **1**, Ir, **2**; 0.012 mmol) react with the methyl ketones CH_3_COR (R = CH_3_, Ph, CF_3_), using the corresponding ketone (0.40 mL) or ketone/CD_2_Cl_2_ mixtures (0.40 mL, 50/50, v/v) as the solvent, affording *Z* and *E* isomers of the compounds [Cp*M(CH_2_COR)(κ^2^*N,N′*-**HL**)][SbF_6_] (R = CH_3_; M = Rh, **3**; Ir, **4**. R = Ph; M = Rh, **5**; Ir, **6**. R = CF_3_; M = Rh, **7**; Ir, **8**) in equilibrium with the corresponding starting compound. Table S1 (SI) shows the equilibrium composition of the solutions, indicating the reaction temperature and the time it took to reach it in each case.

The equilibrium solution was vacuum-concentrated until *ca*. 0.1 mL. The addition of diethyl ether afforded a solid which was filtered off, washed with the precipitant (3 × 5 mL), and vacuum-dried. Table S2 (SI) gathers the composition of the obtained solid determined by ^1^H NMR spectroscopy indicating the reaction temperature in each case.

### Preparation of [Cp*Ir(CH_2_COCF_3_)(κ^2^*N,N′*-HL)][SbF_6_] (**8**)

At 281 K, in a sealed NMR tube, [Cp*Ir(κ^3^*N,N′,N″*-**L**)][SbF_6_] (**2**; 70.0 mg, 0.086 mmol) was dissolved in CD_2_Cl_2_ (0.30 mL), and 0.30 mL of CH_3_COCF_3_ was added. The solution was kept at 281 K for 4 days. Solvents were evaporated under reduced pressure to *ca*. 0.1 mL. The addition of diethyl ether afforded **8** as a yellow solid, which was filtered off, washed with the precipitant (3 × 1 mL), and vacuum-dried. Yield: 72.3 mg, 91%.

### Characterization of Complexes **3**–**8**

#### Compound **3**

HRMS (μ-TOF), C_29_H_39_N_3_ORh [M-SbF_6_]^+^, calcd: 548.2143. Found : 548.2128. IR (cm^–1^): ν(NH) 2964, 2954 (w); ν(C=O) 1631; ν(C=N) 1625; ν(SbF_6_) 651 (s).

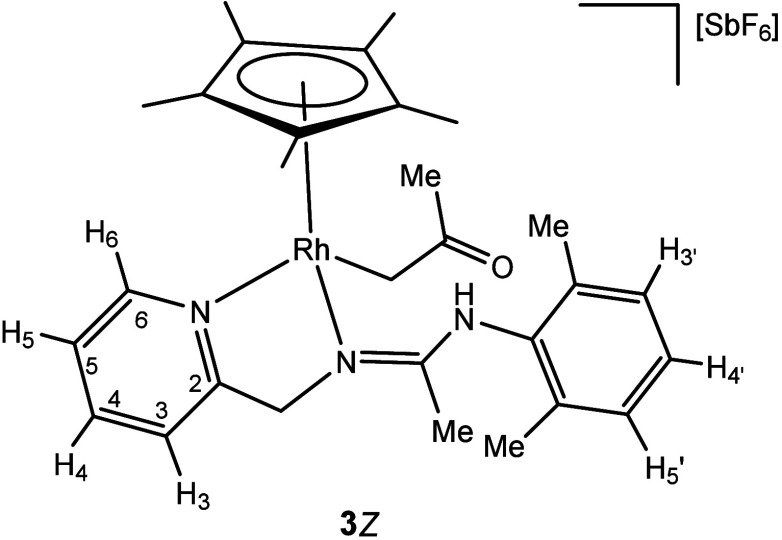



****3***Z*.**^1^H NMR (300.13 MHz, CD_2_Cl_2_, RT, ppm): δ 8.32 (s, NH); 8.24 (d, *J* = 5.6 Hz, 1H, H_6_); 7.24–6.98 (m, 3H, H_3′_, H_4′_, H_5′_); 5.11, 4.87 (AB system, *J* = 17.7 Hz, 2H, CH_2_); 2.74 (dd, *J* = 7.6, 3.6 Hz, 1H, CH_2_CO); 2.21 (m, 1H, CH_2_CO); 2.33, 2.18 (2 × s, 6H, C_6_H_3_*Me*_2_); 2.04 (s, 3H, Me); 1.58 (s, 15H, C_5_Me_5_); 1.20 (s, 3H, COMe). ^13^C{^1^H} NMR (75.48 MHz, CD_2_Cl_2_, RT, ppm): δ 217.81 (d, *J* = 1.5 Hz, C=O); 166.97 (C=N); 161.34 (C_2_); 151.10 (C_6_); 139.34 (C_4_); 126.17 (C_5_); 122.46 (C_3_); 95.79 (d, *J* = 6.4 Hz, *C*_5_Me_5_); 61.21 (CH_2_); 29.60 (CO*Me*); 28.41 (d, *J* = 23.9 Hz, *C*H_2_CO); 18.53, 18.30 (C_6_H_3_*Me*_2_); 16.24 (Me); 9.37 (C_5_*Me*_5_).

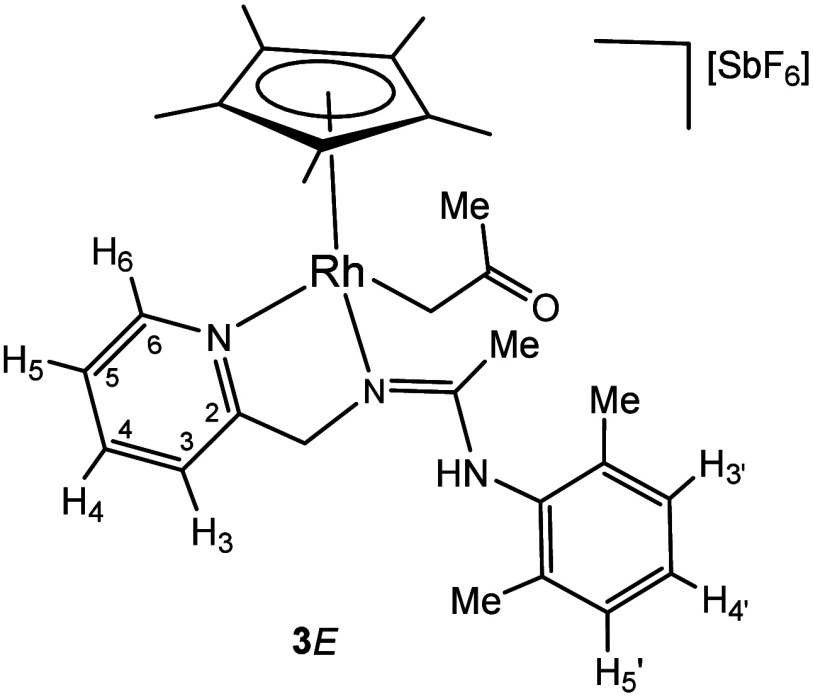



**3*E*.**^1^H NMR (300.13 MHz, CD_2_Cl_2_, RT, ppm): δ 8.18 (d, *J* = 5.6 Hz, 1H, H_6_); 7.93 (m, 1H, H_4_); 7.66 (d, *J* = 7.9 Hz, 1H, H_3_); 7.48 (m, H_5_); 7.27 (s, 1H, NH); 7.24–6.98 (m, 3H, H_3′_, H_4′_, H_5′_); 5.09, 4.97 (AB system, *J* = 19.0 Hz, 2H, CH_2_); 2.83 (pt, *J* = 4.4 Hz, 1H, CH_2_CO); 2.66 (pt, *J* = 4.4 Hz, 1H, CH_2_CO); 2.36, 2.01 (2 × s, 6H, C_6_H_3_*Me*_2_); 1.87 (s, 3H, Me); 1.53 (s, 15H, C_5_Me_5_); 1.05 (s, 3H, COMe). ^13^C{^1^H} NMR (75.48 MHz, CD_2_Cl_2_, RT, ppm): δ 213.28 (d, *J* = 1.5 Hz, C=O); 164.54 (d, *J* = 2.3 Hz, C=N); 161.28 (C_2_); 151.65 (C_6_); 139.54 (C_4_); 126.02 (C_5_); 122.46 (C_3_); 95.88 (d, *J* = 6.4 Hz, *C*_5_Me_5_); 58.45 (CH_2_); 31.26 (d, *J* = 24.1 Hz, *C*H_2_CO); 30.66 (CO*Me*); 23.61 (Me); 19.50, 18.99 (C_6_H_3_*Me*_2_); 9.29 (C_5_*Me*_5_).

#### Compound **4**

HRMS (μ-TOF), C_29_H_39_IrN_3_O [M-SbF_6_]^+^, calcd: 638.2717. Found: 638.2693. IR (cm^–1^): ν(NH) 2930, 2922 (w); ν(C=O) 1645; ν(C=N) 1614; ν(SbF_6_) 653 (s).

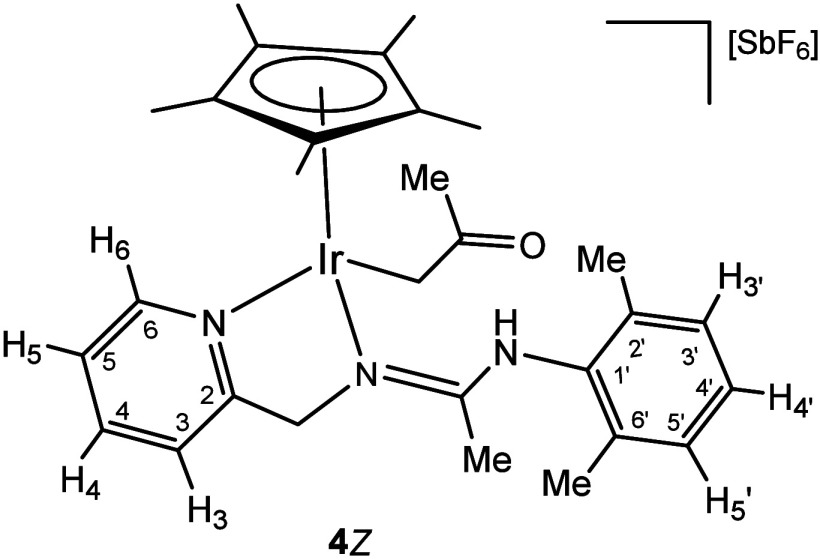



****4***Z*.**^1^H NMR (300.13 MHz, THF-*d*_8_, RT, ppm): δ 8.71 (s, 1H, NH); 8.48 (d, *J* = 6.5 Hz, 1H, H_6_), 7.97 (pt, H_4_); 7.72 (d, *J* = 6.9 Hz, 1H, H_3_); 7.48 (pt, 1H, H_5_), 7.24–6.96 (m, 3H, H_3′_, H_4′_, H_5′_); 5.50, 4.87 (AB system, *J*(AB) = 17.9 Hz, 2H, CH_2_N); 3.12, 2.41 (AB system, *J*(AB) = 9.8 Hz, 2H, CH_2_CO); 2.40, 2.00 (2 × s, 6H, C_6_H_3_*Me*_2_); 2.14 (s, 3H, Me); 1.65 (s, 15H, C_5_Me_5_); 1.17 (s, 3H, COMe). ^13^C{^1^H} NMR (75.48 MHz, THF-*d*_8_, RT, ppm): δ 218.42 (C=O); 166.09 (C=N); 163.27 (C_2_); 151.39 (C_6_); 139.56 (C_4_); 137.50, 137.12, 136.99 (C_1′_, C_2′_, C_6′_); 129.57, 129.53, 126.39 (C_3′_, C_4′_, C_5′_); 126.56 (C_5_); 122.08 (C_3_); 88.50 (*C*_5_Me_5_); 63.36 (CH_2_); 29.77 (CO*Me*); 21.08 (*C*H_2_CO); 19.34, 18.50 (C_6_H_3_*Me*_2_); 15.53 (Me); 8.67 (C_5_*Me*_5_).

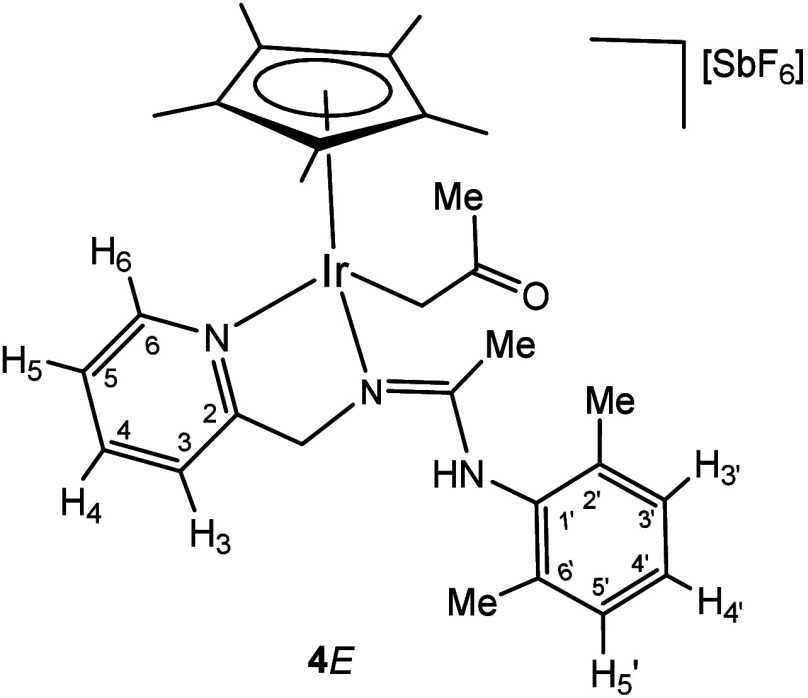



****4***E*.**^1^H NMR (300.13 MHz, THF-*d*_8_, RT, ppm): δ 8.44 (d, *J* = 5.6 Hz, 1H, H_6_); 8.16 (s, 1H, NH); 5.27, 4.94 (AB system, *J*(AB) = 18.5 Hz, 2H, CH_2_N); 2.97, 2.83 (AB system, *J*(AB) = 6.6 Hz, 1H, CH_2_CO); 2.34, 2.25 (2 × s, 6H, C_6_H_3_*Me*_2_); 2.00 (s, 3H, Me); 1.58 (s, 15H, C_5_Me_5_); 1.05 (s, 3H, COMe). ^13^C{^1^H} NMR (75.48 MHz, THF-*d*_8_, RT, ppm): δ 212.38 (C=O); 163.99 (C=N); 162.98 (C_2_); 152.00 (C_6_); 139.39 (C_4_); 137.95, 137.80, 137.04 (C_1′_, C_2′_, C_6′_); 129.36, 129.24, 125.99 (C_3′_, C_4′_, C_5′_); 126.38 (C_5_); 122.26 (C_3_); 88.55 (*C*_5_Me_5_); 60.54 (CH_2_); 31.15 (CO*Me*); 23.74 (Me); 21.72 (*C*H_2_CO); 18.19, 17.87 (C_6_H_3_*Me*_2_); 8.51 (C_5_*Me*_5_).

#### Compound **5**

HRMS (μ-TOF), C_34_H_41_N_3_ORh [M-SbF_6_]^+^, calcd: 610.2313. Found: 610.2299. IR (cm^–1^): ν(NH) 2919 (w); ν(C=O) 1624; ν(C=N) 1624; ν(SbF_6_) 653 (s).

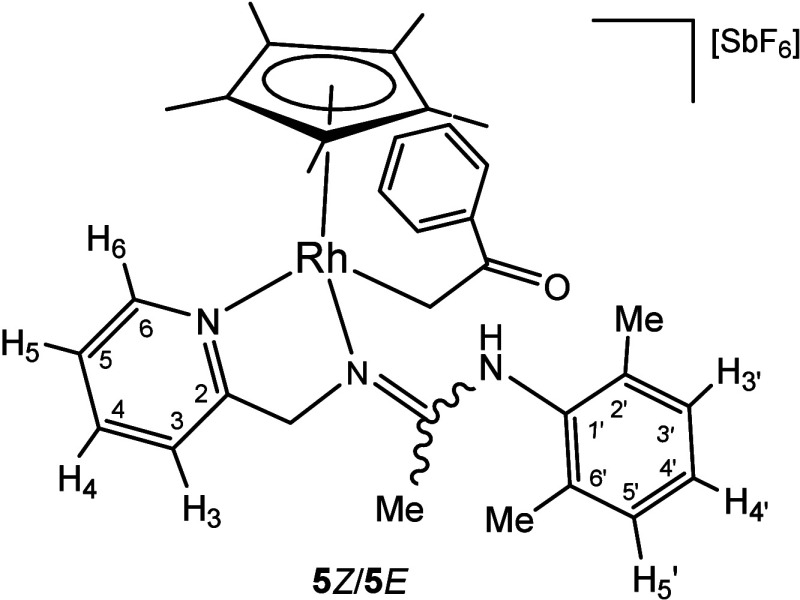



****5***Z*/**5***E* mixture.**^1^H NMR (400.16 MHz, CD_2_Cl_2_, RT, ppm): δ 8.51 (brs, NH); 8.16 (d, *J* = 5.5 Hz, H_6_); 7.78 (d, *J* = 5.5 Hz, H_3_); 7.40–6.90 (brm); 5.11, 4.90 (AB system, *J*(AB) = 17.8 Hz, CH_2_); 4.87, 4.80 (AB system, *J*(AB) = 18.8 Hz, CH_2_); 3.43, 3.19 (2 × m, CH_2_CO); 3.06, 3.00 (2 × m, CH_2_CO); 2.40, 2.34, 2.10, 1.98, 1.69 (5 × s, C_6_H_3_*Me*_2_; Me); 1.55 (s, C_5_Me_5_); 1.55, 1.48 (2 × s, C_5_Me_5_). ^13^C{^1^H} NMR (100.62 MHz, CD_2_Cl_2_, RT, ppm): δ 207.29, 204.82 (C=O); 167.25, 164.48 (C=N); 161.18, 160.77 (C_2_); 151.28, 151.25 (C_6_); 139.43, 138.64 (C_4_); 138.04, 137.14, 136.91, 136.65, 136.55, 136.21 (C_1′_, C_2′_, C_6′_); 132.75, 131.39 (Ph); 129.80, 129.75, 129.10, 129.01, 128.95, 128.50 (C_3′_, C_4′_, C_5′_, Ph); 126.03, 125.34 (C_5_); 122.06, 121.77 (C_3_); 96.02 (d, *J* = 6.5 Hz), 95.96 (d, *J* = 6.3 Hz) (*C*_5_Me_5_); 61.28, 58.40 (CH_2_); 25.62 (d, *J* = 23.6 Hz), 22.55 (d, *J* = 23.2 Hz) (*C*H_2_CO); 23.59, 16.43 (Me); 19.60, 19.23, 18.57, 18.51 (C_6_H_3_*Me*_2_); 9.36 (C_5_*Me*_5_).

#### Compound **6**

HRMS (μ-TOF), C_34_H_41_IrN_3_O [M-SbF_6_]^+^, calcd: 700.2856. Found: 700.2873. IR (cm^–1^): ν(NH) 2927 (w); ν(C=O) 1683; ν(C=N) 1631; ν(SbF_6_) 655 (s).

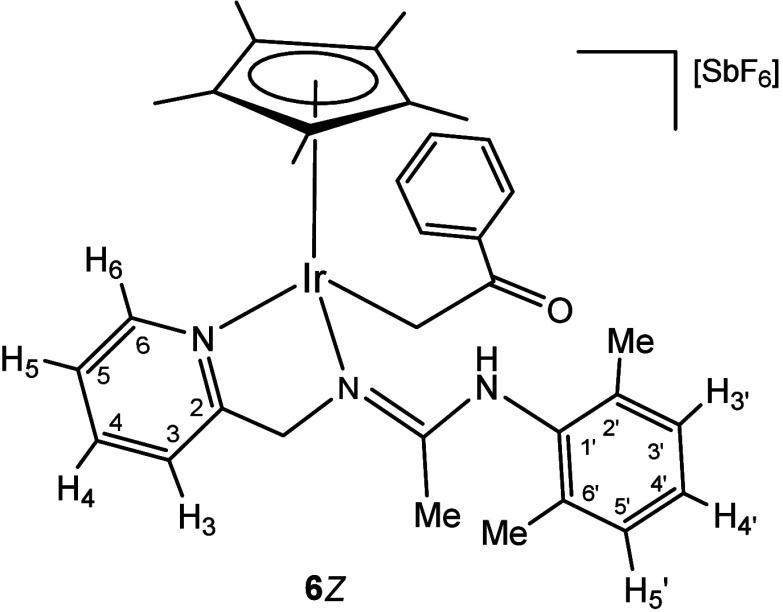



****6***Z*.**^1^H NMR (400.16 MHz, CD_2_Cl_2_, RT, ppm): δ 8.56 (s, 1H, NH); 8.20 (d, *J* = 5.8 Hz, 1H, H_6_); 7.87–7.67 (m, 3H, H_4_, H_2_Ph, H_6_Ph); 7.55 (d, *J* = 7.9 Hz, 1H, H_3_); 7.47 (t, *J* = 7.6 Hz, 1H, H_4_Ph); 7.35 (pt, *J* = 7.6 Hz, 2H, H_3_Ph, H_5_Ph); 7.28–6.92 (m, 4H, H_5_, H_3′_, H_4′_, H_5′_); 5.34–4.79 (AB system, *J*(AB) = 17.7 Hz, 2H, CH_2_); 3.41, 3.23 (AB system, *J*(AB) = 9.0 Hz, 2H, CH_2_CO); 2.40, 2.10 (2 × s, 6H, C_6_H_3_*Me*_2_); 2.19 (s, 3H, Me);1.53 (s, 15H, C_5_Me_5_). ^13^C{^1^H} NMR (100.62 MHz, CD_2_Cl_2_, RT, ppm): δ 208.34 (C=O); 165.86 (C=N); 162.19 (C_2_); 150.88 (C_6_); 140.37 (C_1_Ph); 139.28 (C_4_); 137.97, 136.84, 136.05 (C_1′_, C_2′_, C_6′_); 129.80, 129.75, 129.10 (C_3′_, C_4′_, C_5′_); 129.01, 128.95, 128.50 (C_2_Ph, C_3_Ph, C_4_Ph, C_5_Ph, C_6_Ph); 126.33 (C_5_); 121.65 (C_3_); 88.68 (*C*_5_Me_5_); 63.15 (CH_2_); 19.62, 19.25 (C_6_H_3_*Me*_2_); 16.17 (Me); 14.22 (*C*H_2_CO); 9.22 (C_5_*Me*_5_).

#### Compound **7**

HRMS (μ-TOF), C_29_H_36_F_3_N_3_ORh [M-SbF_6_]^+^, calcd: 602.1860. Found: 602.1849. IR (cm^–1^): ν(NH) 3383, 2924 (w); ν(C=O) 1663; ν(C=N) 1614; ν(SbF_6_) 652 (s).

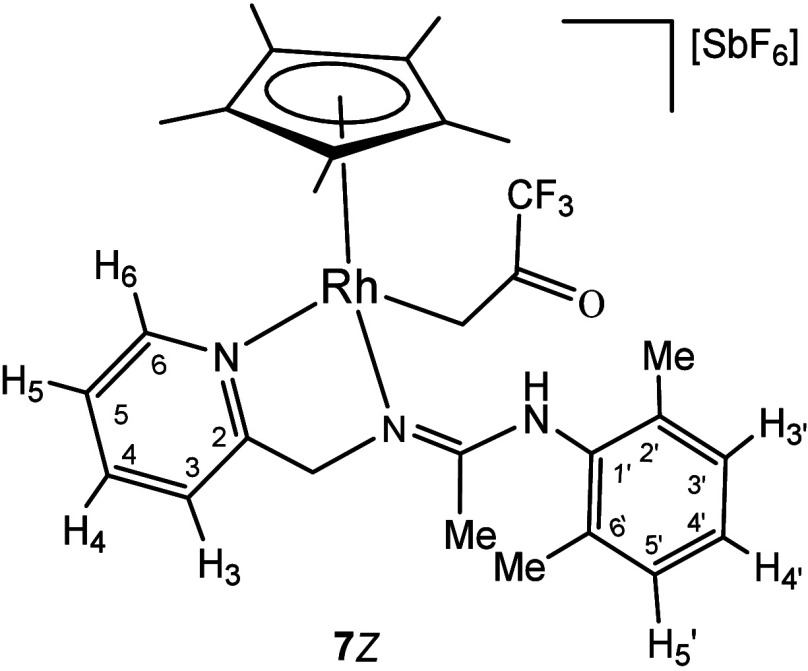



****7***Z*.**^1^H NMR (300.13 MHz, CD_2_Cl_2_, RT, ppm): δ 8.15 (d, *J* = 5.6 Hz, 1H, H_6_); 6.62 (s, 1H, NH); 5.11, 4.96 (AB system, *J*(AB) = 18.6 Hz, 2H, CH_2_); 3.10, 2.61 (2 × m, 2H, CH_2_CO); 2.38, 2.19 (2 × s, 6H, C_6_H_3_*Me*_2_); 1.88 (s, 3H, Me); 1.60 (s, 15H, C_5_Me_5_). ^13^C{^1^H} NMR (100.62 MHz, CD_2_Cl_2_, 233 K, ppm): δ 166.14 (C=N); 160.95 (C_2_); 150.58 (C_6_); 139.55 (C_4_); 137.23, 136.51, 134.56 (C_1′_, C_2′_, C_6′_); 129.55, 129.35, 128.91 (C_3′_, C_4′_, C_5′_); 125.76 (C_5_); 121.89 (C_3_); 116.17 (q, *J* = 293.8 Hz, CF_3_); 96.42 (d, *J* = 6.3 Hz, *C*_5_Me_5_); 60.46 (CH_2_); 19.46 (d, *J* = 22.1 Hz, *C*H_2_CO); 19.43, 19.27 (C_6_H_3_*Me*_2_); 15.74 (Me); 9.25 (C_5_*Me*_5_). ^19^F NMR (376.46 MHz, CD_2_Cl_2_, 233 K): δ −79.48 (s).

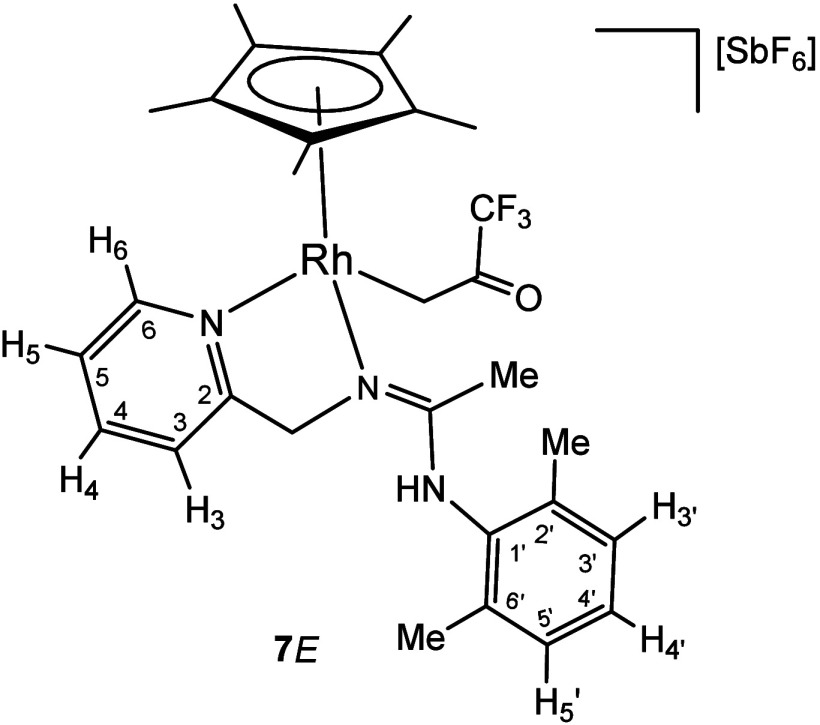



****7***E*.**^1^H NMR (300.13 MHz, CD_2_Cl_2_, RT, ppm): δ 8.13 (d, *J* = 5.5 Hz, 1H, H_6_); 7.91 (td, *J* = 7.7, 1.5 Hz, 1H, H_4_); 7.62 (d, 1H, H_3_); 7.48 (m, 1H, H_5_); 7.30–7.00 (m, 4H, NH, H_3′_, H_4′_, H_5′_); 5.04 (bs, 2H, CH_2_); 3.10 (m, 1H, CH_2_CO); 2.83 (ddq, *J* = 5.2, 3.4, 1.6 Hz, 1H, CH_2_CO); 2.34, 2.17 (2 × s, 6H, C_6_H_3_*Me*_2_); 1.92 (s, 3H, Me); 1.54 (s, 15H, C_5_Me_5_). ^13^C{^1^H} NMR (100.62 MHz, CD_2_Cl_2_, 233 K, ppm): δ 196.10 (q, *J* = 31.2 Hz, C=O); 163.71 (C=N); 161.14 (C_2_); 151.31 (C_6_); 139.36 (C_4_); 136.87, 136.15, 135.82 (C_1′_, C_2′_, C_6′_); 129.02, 128.80, 128.72 (C_3′_, C_4′_, C_5′_); 125.60 (C_5_); 121.71 (C_3_); 115.92 (q, *J* = 294.1 Hz, CF_3_); 95.89 (d, *J* = 6.3 Hz, *C*_5_Me_5_); 57.97 (CH_2_); 23.46 (Me); 22.91 (d, *J* = 22.1 Hz, *C*H_2_CO); 18.34, 17.78 (C_6_H_3_*Me*_2_); 9.11 (C_5_*Me*_5_). ^19^F NMR (376.46 MHz, CD_2_Cl_2_, 233 K): δ −78.68 (s).

#### Compound **8**

Anal. Calcd for C_29_H_36_F_9_N_3_OIrSb: C, 37.55; H, 3.91; N, 4.53. Found: C, 37.69; H, 3.84; N, 4.69. HRMS (μ-TOF), C_29_H_36_F_3_N_3_OIr [M-SbF_6_]^+^, calcd: 692.2440. Found: 692.2436. IR (cm^–1^): ν(NH) 3384, 2926 (w); ν(C=O) 1682; ν(C=N) 1634; ν(SbF_6_) 655 (s).

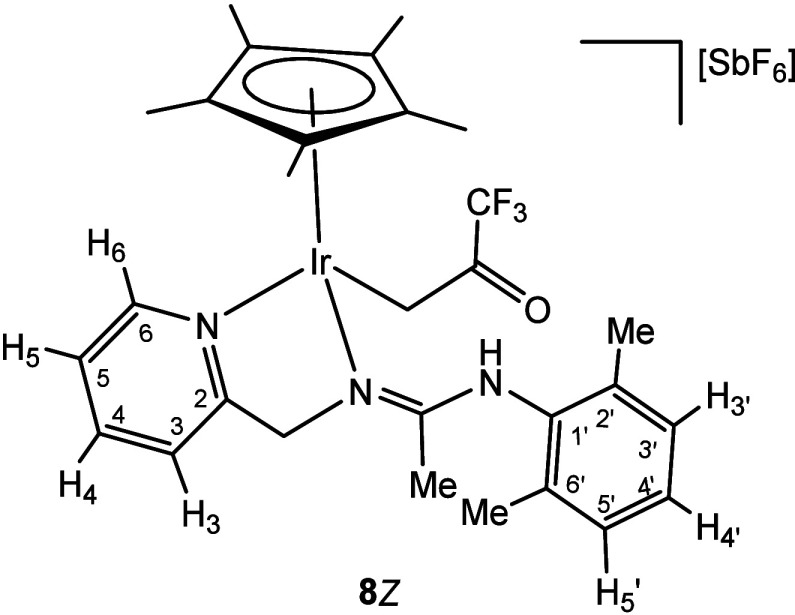



****8***Z*.**^1^H NMR (400.16 MHz, CD_2_Cl_2_, RT, ppm): δ 8.23 (d, *J* = 5.8 Hz, 1H, H_6_); 7.93 (pt, *J* = 7.8 Hz, 1H, H_4_); 7.64 (d, 1H, H_3_); 7.43 (m, 1H, H_5_); 7.31–7.09 (m, 3H, H_3′_, H_4′_, H_5′_); 6.92 (s, 1H, NH); 5.34, 4.87 (AB system, *J*(AB) = 18.0 Hz, 2H, CH_2_N); 3.51 (d, *J* = 8.0 Hz, 1H, CH_2_CO); 2.76 (d, 1H, CH_2_CO); 2.37, 2.13 (2 × s, 6H, C_6_H_3_*Me*_2_); 2.18 (s, 3H, Me); 1.63 (s, 15H, C_5_Me_5_). ^13^C{^1^H} NMR (100.62 MHz, CD_2_Cl_2_, RT, ppm): δ 198.58 (q, *J* = 31.6 Hz, C=O); 165.65 (C=N); 162.62 (C_2_); 150.78 (C_6_); 139.84 (C_4_); 137.54, 136.72, 134.99 (C_1′_, C_2′_, C_6′_); 130.10, 129.93, 129.38 (C_3′_, C_4′_, C_5′_); 126.42 (C_5_); 122.05 (C_3_); 116.73 (q, *J* = 294.8 Hz, CF_3_); 89.63 (*C*_5_Me_5_); 62.72 (CH_2_); 19.51, 19.47 (C_6_H_3_*Me*_2_); 15.76 (Me); 10.83 (*C*H_2_CO); 9.33 (C_5_*Me*_5_). ^19^F NMR (376.70 MHz, CD_2_Cl_2_): δ −79.67 (s).

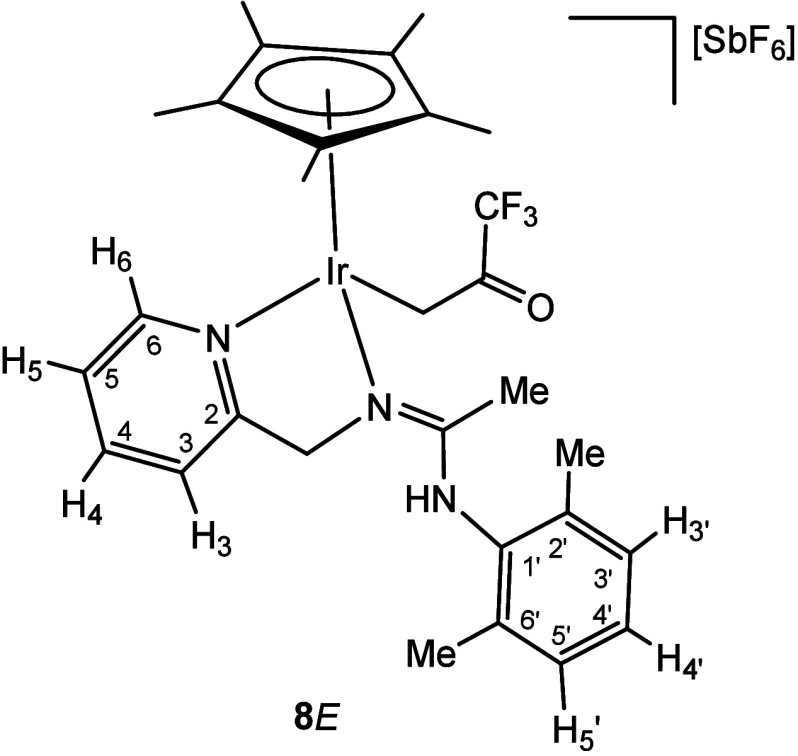



****8***E*.**^1^H NMR (400.16 MHz, CD_2_Cl_2_, RT, ppm): δ 8.16 (d, *J* = 5.8 Hz, 1H, H_6_); 7.89 (m, 1H, H_4_); 7.69 (d, *J* = 8.0 Hz, 1H, H_3_); 7.39 (pt, 1H, H_5_); 7.31–7.09 (m, 4H, H_3′_, H_4′_, H_5′_, NH); 5.21, 4.91 (AB system, *J*(AB) = 18.4 Hz, 2H, CH_2_); 3.45 (d, *J* = 6.8 Hz, 1H, CH_2_CO); 2.99 (d, 1H, CH_2_CO); 2.33, 2.19 (2 × s, 6H, C_6_H_3_*Me*_2_); 2.01 (s, 3H, Me); 1.54 (s, 15H, C_5_*Me*_5_). ^13^C{^1^H} NMR (100.62 MHz, CD_2_Cl_2_, RT, ppm): δ 197.84 (q, *J* = 31.1 Hz, C=O); 163.94 (C=N); 162.92 (C_2_); 151.40 (C_6_); 139.59 (C_4_); 137.31, 136.58, 136.16 (C_1′_, C_2′_, C_6′_); 129.52, 129.35, 129.25 (C_3′_, C_4′_, C_5′_); 126.11 (C_5_); 122.05 (C_3_); 116.60 (q, *J* = 294.8 Hz, CF_3_); 89.01 (*C*_5_Me_5_); 60.20 (CH_2_N); 23.66 (Me); 18.44, 18.07 (C_6_H_3_*Me*_2_); 13.89 (*C*H_2_CO); 9.16 (C_5_*Me*_5_). ^19^F NMR (376.70 MHz, CD_2_Cl_2_): δ −79.30 (s).

### Preparation and Characterization of the Iridium Complexes **9** and **10**

#### Preparation of Complex **9**

Under argon, at room temperature, to a sealed NMR tube containing a solution of [Cp*Ir(κ^3^*N*,*N′,N″-***L**)][SbF_6_] (**2**; 40.0 mg, 0.05 mmol) in THF-*d*_8_ (0.40 mL), hexafluoroacetone trihydrate (0.25 mmol) and 4 Å molecular sieves (15.0 mg) were added. The suspension was heated to 353 K overnight and then it was filtered. The resulting filtrate was vacuum-concentrated until ca. 0.1 mL and addition of diethyl ether afforded a yellow solid which was washed with the precipitant (3 × 5 mL) and vacuum-dried. Yield: 39.4 mg, 82%. The solid consists of a mixture of isomers **9***Z* and **9***E* in 99/1 molar ratio, respectively.

##### Compound **9**

Anal. Calcd for C_29_H_33_F_12_IrN_3_OSb·H_2_O: C, 34.85; H, 3.53; N, 4.20. Found: C, 34.62; H, 3.71; N, 4.11. HRMS (μ-TOF), C_29_H_33_F_6_IrN_3_O: [M-SbF_6_]^+^, calcd: 746.2157. Found: 746.2145. IR (cm^–1^): ν(NH) 2950 (w); ν(C=N) 1612; ν(C–O) 1201; ν(SbF_6_) 654 (s).

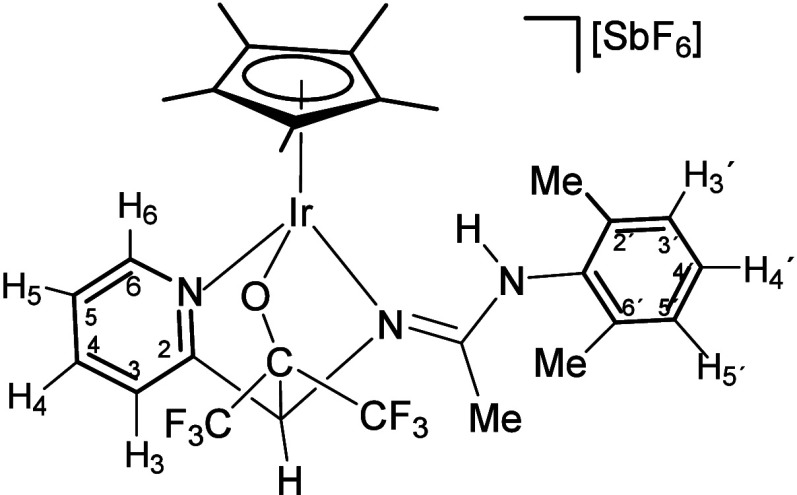



****9***Z*.**^1^H NMR (400.16 MHz, CD_2_Cl_2_, RT, ppm): δ 8.77 (d, *J* = 6.7 Hz, 1H, H_6_); 8.08 (pt, 1H, H_4_); 7.83 (d, 1H, *J* = 7.7 Hz, H_3_); 7.58 (pt, 1H, H_5_); 7.36–7.14 (m, 3H, H_3′_, H_4′_, H_5′_); 6.92 (s, 1H, NH); 5.85 (s, 1H, CH); 2.29, 2.03 (2 × s, 6H, C_6_H_3_*Me*_2_); 1.92 (s, 3H, Me), 1.79 (s, 15H, C_5_Me_5_). ^13^C{^1^H} NMR (100.62 MHz, CD_2_Cl_2_, RT, ppm): δ 164.54 (C=N); 157.90 (C_2_); 149.82 (C_6_); 140.75 (C_4_): 137.17, 137.04, 133.30 (C_1′_, C_2′_, C_6′_); 130.16, 130.07, 129.96 (C_3′_, C_4′_, C_5′_); 126.30 (C_5_); 124.55 (q, *J* = 295.8 Hz, CF_3_); 124.32 (C_3_); 122.98 (q, *J* = 290.1 Hz, CF_3_); 87.56 (*C*_5_Me_5_); 83.13 (m, CO); 75.14 (CH); 18.55, 18.38 (C_6_H_3_*Me*_2_); 14.34 (Me); 9.79 (C_5_*Me*_5_). ^19^F{^1^H} NMR (282.38 MHz, CD_2_Cl_2_, RT, ppm): δ −71.70 (q, *J* = 11.2 Hz); −73.79 (q).

#### Preparation of Complex **10**

Under argon, a solution of [Cp*Ir(κ^3^*N*,*N′*,*N″-***L**)][SbF_6_] (**2**; 40.0 mg, 0.05 mmol) in CF_3_COPh (0.4 mL) was heated to 423 K in an NMR tube. The reaction was monitored by ^1^H NMR and complete disappearance of the signals of the starting product was verified after 5 h. The resulting solution was vacuum-concentrated until ca. 0.1 mL and addition of diethyl ether afforded a yellow solid which was filtered off, washed with the precipitant (3 × 1 mL) and vacuum-dried. The solid was recrystallized from CH_2_Cl_2_/diethyl ether. Yield: 33.5 mg, 69%. The obtained product was a mixture of four isomers in about 92/4/3/1 molar ratio. The major component was characterized as the (*S*_Ir_,*R*_C(16)_,*R*_C(27)_)-**10** isomer by X-ray diffraction means. Its NMR spectroscopic data are collected below. In the recorded spectra, signals corresponding to the remaining isomers are also observed.

##### Compound **10** (Major Isomer)

Anal. Calcd for C_34_H_38_F_9_IrN_3_OSb: C, 41.27; H, 3.87; N, 4.24. Found: C, 41.21; H, 3.84; N, 4.29. HRMS (μ-TOF), C_34_H_38_F_3_IrN_3_O [M-SbF_6_]^+^, calcd: 754.2596. Found: 754.2600. IR (cm^–1^): ν(NH) 2969 (w); ν(C=N) 1617 (m); ν(C–O) 1157 (m); ν(SbF_6_) 654 (s).

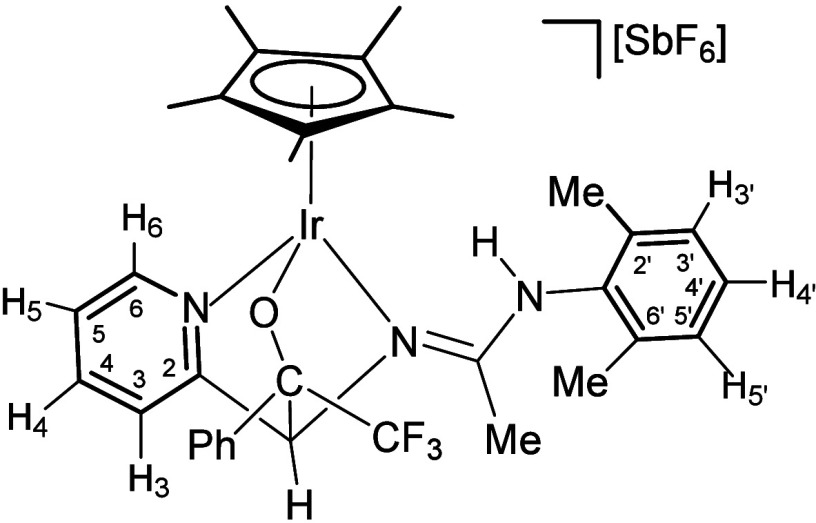



^1^H NMR (300.13 MHz, CD_2_Cl_2_, RT, ppm): δ 8.63 (d, *J* = 5.7 Hz, 1H, H_6_); 7.54 (pt, *J* = 7.7, 1H, H_4_); 7.37–6.89 (m, 10H, H_3_, H_5_, H_3′_, H_4′_, H_5′_, 5 × H_Ar_); 6.95 (s, 1H, NH); 5.94 (s, 1H, CH); 2.35, 2.08 (2 × s, 6H, C_6_H_3_*Me*_2_); 2.01 (s, 3H, Me); 1.84 (s, 15H, C_5_Me_5_). ^13^C{^1^H} NMR (75.48 MHz, CD_2_Cl_2_, RT, ppm): 163.59 (C=N); 159.11 (C_2_); 148.72 (C_6_); 139.81 (C_ipsoAr_); 139.02 (C_4_); 137.47, 137.13, 133.69 (C_1′_, C_2′_, C_6′_); 130.05, 129.94, 129.09 (C_3′_, C_4′_, C_5′_); 128.99, 128.67, 128.38, 127.64, 126.14 (5 × C_Ar_); 127.15 (q, *J* = 293.1 Hz, CF_3_); 125.10 (C_5_); 124.13 (C_3_); 87.05 (*C*_5_Me_5_); 81.49 (q, *J* = 26.0 Hz, CO); 78.72 (CH); 18.66, 18.46 (C_6_H_3_*Me*_2_); 14.02 (Me); 9.83 (C_5_*Me*_5_). ^19^F{^1^H} NMR (282.38 MHz, CD_2_Cl_2_, RT, ppm): δ −72.96 (s).

### Preparation and Characterization of the Rhodium Complexes **11** and **12**

#### Preparation of Complex **11**

Under argon, at room temperature, to a Schlenk flask containing a solution of [Cp*Rh(κ^3^*N*,*N′*,*N″-***L**)][SbF_6_] (**1**; 72.6 mg, 0.10 mmol) in dichloromethane (5 mL), hexafluoroacetone trihydrate (0.15 mmol), and 4 Å molecular sieves (25.0 mg) were added. The suspension was heated to 323 K for 48 h and then was filtered to remove the solids. The resulting solution was vacuum-concentrated until ca. 0.5 mL, and the addition of diethyl ether afforded an orange solid, which was washed with the precipitant (3 × 5 mL) and vacuum-dried. Yield: 62.4 mg, 70%.

##### Compound **11**

Anal. Calcd for C_29_H_33_F_12_N_3_ORhSb: C, 39.04; H, 3.73; N, 4.71. Found: C, 38.93; H, 3.48; N, 5.09. HRMS (μ-TOF), C_29_H_33_F_6_N_3_ORh [M-SbF_6_]^+^, calcd: 656.1577. Found: 656.1576. IR (cm^–1^): ν(N–H) 2960 (w); ν(C=N) 1631 (m); ν(C–O) 1190 (m); ν(SbF_6_) 654 (s).

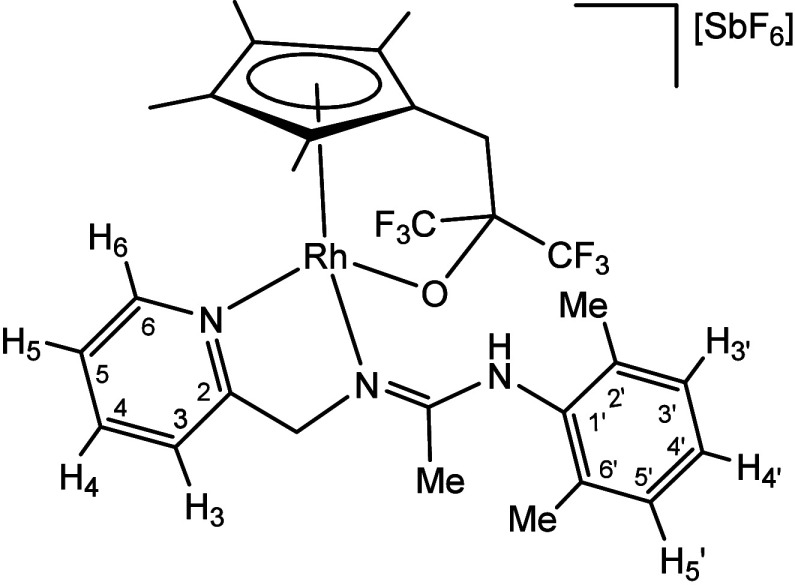



^1^H NMR (300.13 MHz, CD_2_Cl_2_, RT, ppm): δ 9.42 (s, 1H, NH); 8.93 (d, *J* = 5.6 Hz, 1H, H_6_); 8.02 (pd, *J* = 7.7 Hz, 1H, H_4_); 7.69–7.59 (m, 2H, H_3_, H_5_); 7.27–6.96 (m, 3H, H_3′_, H_4′_, H_5′_); 5.09, 4.71 (AB system, *J*(AB) = 17.0 Hz, 2H, CH_2_N); 3.12, 3.01 (AB system, *J*(AB) = 15.8 Hz, 2H, CH_2_CO); 2.41, 2.13 (2 × s, 6H, C_6_H_3_*Me*_2_); 1.97 (Me); 1.97, 1.93, 1.72, 1.06 (4 × s, 12H, C_5_*Me*_4_(CH_2_CO(CF_3_)_2_). ^13^C{^1^H} NMR (100.62 MHz, CD_2_Cl_2_, RT, ppm): δ 169.57 (C=N); 161.91 (C_2_); 150.52 (q, *J* = 3.0 Hz, C_6_); 140.45 (C_4_); 137.43, 135.87, 135.51 (C_1′_, C_2′_, C_6′_); 129.34, 129.27, 128.52 (C_3′_, C_4′_, C_5′_); 126.32 (C_5_); 125.46 (q, *J* = 289.8 Hz, CF_3_); 124.60 (CF_3_); 122.97 (C_3_); 111.11 (d, *J* = 7.5 Hz, RhC); 103.73 (pspt, *J* = 27.1 Hz, *C*(CF_3_)_2_); 103.09 (d, *J* = 7.0 Hz, RhC); 91.83 (d, *J* = 7.6 Hz, RhC); 91.50 (d, *J* = 8.7 Hz, RhC); 83.84 (d, *J* = 9.6 Hz, RhC); 60.82 (CH_2_N); 31.14 (CH_2_CO); 18.88, 17.94 (C_6_H_3_*Me*_2_); 16.02 (Me); 9.84 (d, *J* = 3.0 Hz), 9.74, 8.86 (d, *J* = 4.0 Hz), 8.64 (C_5_*Me*_4_(CH_2_CO(CF_3_)_2_). ^19^F{^1^H} NMR (282.38 MHz, CD_2_Cl_2_, RT, ppm): δ −77.66 (q, *J* = 10.6 Hz); −78.19 (q).

#### Preparation of Complex **12**

Under argon, a solution of [Cp*Rh(κ^3^*N*,*N′*,*N″-***L**)][SbF_6_] (**1**; 72.6 mg, 0.10 mmol) in CF_3_COPh (2 mL) was heated to 333 K. The reaction was monitored by ^1^H NMR spectroscopy, and complete disappearance of the signals of the starting product was verified after 1 h of treatment. The resulting solution was vacuum-concentrated until ca. 0.5 mL, and addition of diethyl ether afforded an orange solid, which was filtered off, washed with the precipitant (3 × 5 mL), and vacuum-dried. Yield: 77.2 mg, 89%.

##### Compound **12**

Anal. Calcd for C_34_H_38_F_9_N_3_ORhSb: C, 45.36; H, 4.25; N, 4.67. Found: C, 44.99; H, 3.97; N, 4.80. HRMS (μ-TOF), C_34_H_38_F_3_N_3_ORh [M-SbF_6_]^+^, calcd: 664.2017. Found: 664.2023. IR (cm^–1^): ν(NH) 2872 (w); ν(C=N) 1634 (m); ν(C–O) 1143 (m); ν(SbF_6_) 653 (s).

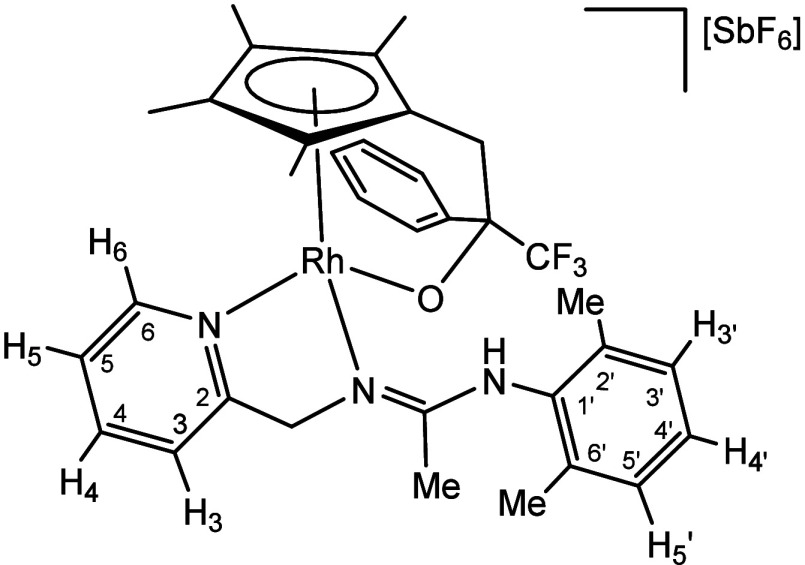



^1^H NMR (300.13 MHz, CD_2_Cl_2_, RT, ppm): δ 10.81 (s, 1H, NH); 8.28 (d, *J* = 5.3 Hz, 1H, H_6_); 7.99 (pt, *J* = 7.8 Hz, 1H, H_4_); 7.68 (d, *J* = 7.0 Hz, 2H, H_Ar_); 7.62 (d, 1H, H_3_); 7.53–7.37 (m, 4H, H_5_, 3 × H_Ar_); 7.23–6.99 (m, 3H, H_3′_, H_4′_, H_5′_); 5.12, 4.73 (AB system, *J*(AB) = 17.0 Hz, 2H, CH_2_N); 3.48, 3.06 (AB system, *J*(AB) = 14.2 Hz, 2H, CH_2_CO); 2.43, 1.98 (2 × s, 6H, C_6_H_3_*Me*_2_); 2.20, 1.68, 1.17, 0.92 (4 × s, 12H, C_5_*Me*_4_(CH_2_CO(CF_3_)(Ph)); 2.00 (s, 3H, Me). ^13^C{^1^H} NMR (75.48 MHz, CD_2_Cl_2_, RT, ppm): δ 170.17 (C=N); 162.48 (C_2_); 151.27 (C_6_); 143.71 (C_ipsoAr_); 140.25 (C_4_); 137.60, 136.37, 135.96 (C_1′_, C_2′_, C_6′_); 129.54, 129.22, 129.09, 128.73, 128.18 (C_3′_, C_4′_, C_5′_, C_Ar_); 125.48 (C_5_); 125.47 (q, *J* = 288.3 Hz, CF_3_); 122.63 (C_3_); 114.52 (d, *J* = 7.4 Hz, RhC); 103.04 (q, *J* = 26.0 Hz, *C*CF_3_); 99.25 (d, *J* = 7.3 Hz, RhC); 96.96 (d, *J* = 6.6 Hz, RhC); 85.73 (d, *J* = 8.9 Hz, RhC); 84.93 (d, *J* = 9.3 Hz, RhC); 60.47 (CH_2_N); 35.14 (CH_2_CO); 19.05, 18.00 (C_6_H_3_*Me*_2_); 16.19 (Me); 10.47, 9.72, 8.54, 8.49 (C_5_Me_4_(CH_2_CO(CF_3_)(Ph)). ^19^F{^1^H} NMR (282.38 MHz, CD_2_Cl_2_, RT, ppm): δ −80.85 (s).

### Crystal Structure Determination of Complexes **8***Z*, **10**, **11**, and **14**

Suitable single crystals for the X-ray diffraction experiments were obtained for complexes **8***Z*, **10, 11,** and **14** by slow diffusion of *n*-hexane in concentrated CH_2_Cl_2_/diethyl ether solutions.

Diffracted intensity data were measured at low temperature 100(2) K on a D8 VENTURE diffractometer, with Mo Kα radiation (λ = 0.71073 Å) using ω and φ scans with narrow frames strategies. Data were integrated and corrected from absorption effects with SAINT^32^ and SADABS^33^ programs, included in the APEX4 package.^34^ Structures were solved by direct methods with SHELXS,^35^ completed by reiterative difference Fourier synthesis and refined by full-matrix least-squares on *F*^2^ with SHELXL program^36^ included in the Olex2 program.^37^ Most of the hydrogen atoms (except those involved in hydrogen bond interactions) were included in the models in calculated positions and refined with a riding model. Interacting hydrogen atoms of N–H and O–H fragments have been included in the model in observed positions and refined with a restrain in N–H bond length (compound **8***Z*) or freely refined (compounds **11** and **14**).

#### Crystal Data for Complex **8***Z*

C_29_H_36_F_9_IrN_3_OSb·CH_2_Cl_2_; *M*_r_ = 1012.51; yellow needle, 0.036 × 0.070 × 0.200 mm^3^; monoclinic *P*2_1_/*c*; *a* = 12.5738(6) Å, *b* = 12.7268(6) Å, *c* = 23.1329(11) Å, β = 101.231(2)°; *V* = 3630.9(3) Å^3^, *Z* = 4, *D*_c_ = 1.852 g/cm^3^; μ = 4.627 cm^–1^. Min and max absorption correction factors: 0.6018 and 0.7457. 2θ_max_ = 56.602°; 90969 reflections measured, 9007 unique. *R*_int_ = 0.0520; number of data/restraint/parameters: 9007/1/409. *R*_1_ = 0.0393 [8008 reflections, *I* > 2σ(*I*)], *wR*2 = 0.0993 (all data); largest difference peak 2.559 e·Å^–3^.

#### Crystal Data for Complex **10**

C_34_H_38_F_9_IrN_3_OSb·CH_2_Cl_2_; *M*_r_ = 1074.55; yellow plate, 0.050 × 0.120 × 0.210 mm^3^; triclinic *P*1; *a* = 8.6540(1) Å, *b* = 10.4725(1) Å, *c* = 10.8662(1) Å, α = 77.538(2)°, β = 76.671(2)°, γ = 86.501(2)°; *V* = 935.62(2) Å^3^, *Z* = 1, *D*_c_ = 1.907 g/cm^3^; μ = 4.495 cm^–1^. Min and max. absorption correction factors: 0.5879 and 0.7484. 2θ_max_ = 61.018°; 71457 reflections measured, 11238 unique. *R*_int_ = 0.0263; number of data/restraint/parameters 11238/3/477. *R*_1_ = 0.0144 [11238 reflections, I > 2σ(I)], *wR2* = 0.0342 (all data); largest difference peak 1.435 e·Å^–3^; Flack parameter:^38^ −0.0115(10).

#### Crystal Data for Complex **11**

C_29_H_33_F_12_N_3_ORhSb; *M*_r_ = 892.24; orange block, 0.070 × 0.100 × 0.170 mm^3^; monoclinic *P2*_1_*/c*; *a* = 11.7055(5) Å, *b* = 13.7874(5) Å, *c* = 20.5268(7) Å, β = 105.5850(10)°; *V* = 3191.0(2) Å^3^, *Z* = 4, *D*_c_ = 1.857 g/cm^3^; μ = 1.463 cm^–1^. Min and max. absorption correction factors: 0.6935 and 0.7461. 2θ_max_ = 61.070°; 162633 reflections measured, 9720 unique. *R*_int_ = 0.0398; number of data/restraint/parameters 9720/0/435. *R*_1_ = 0.0227 [9087 reflections, *I* > 2σ(*I*)], *wR2* = 0.0584 (all data); largest difference peak 1.307 e·Å^–3^.

#### Crystal Data for Complex **14**

C_42_H_43_F_12_N_3_O_2_RhSb; *M*_r_ = 1074.45; orange prism, 0.063 × 0.108 × 0.229 mm^3^; monoclinic *P2*_1_*/c*; *a* = 12.2008(4) Å, *b* = 13.2903(5) Å, *c* = 25.7539(8) Å, β = 95.6050(10)°; *V* = 4156.1(2) Å^3^, *Z* = 4, *D*_c_ = 1.717 g/cm^3^; μ = 1.142 cm^–1^. Min and max. absorption correction factors: 0.6828 and 0.7457. 2θ_max_ = 56.614°; 211811 reflections measured, 10327 unique. *R*_int_ = 0.0426; number of data/restraint/parameters 10327/0/564. *R*_1_ = 0.0394 [9649 reflections, *I* > 2σ(*I*)], *wR2* = 0.0959 (all data); largest difference peak 1.558 e·Å^–3^.

## Computational Section

### DFT Calculations

Molecular structure optimizations and frequencies calculations were carried out with Gaussian16 (revision C.01)^39^ using the method B97D3,^40^ including the D3 dispersion correction scheme by Grimme with Becke–Johnson damping.^41^ The def2svp^42^ basis and pseudo potential were used for all atoms, and the “ultrafine” grid was employed in all calculations. Stationary points were characterized by vibrational analysis. The structures were optimized in dichloromethane or THF (298 K, 1 atm) using the CPCM method.^43^ In selected cases, energy values were refined at the level M06^44^/def2tzvp^42^//B97D3/def2svp. Energy and Gibbs free energy values are given in Table S4.

## Supporting Information Available

Coordinates of calculated structures (XYZ)
Composition of equilibrium mixtures and isolated solids. NMR spectra; thermodynamic data for the equilibria **1** ⇌ **7***Z* and **1** ⇌ **7***E*; relevant NOE interactions; selected mass spectra; calculated energies (PDF)

## Supplementary Material

ic4c03214_si_001.xyz
