# Supplementary material for: Diverse and Selective Metal–Ligand Cooperative Routes for Activating Non-Functionalized Ketones
Source: Inorg Chem. 2025 Jan 31;64(5):2188–206. doi: 10.1021/acs.inorgchem.4c03214 (PMC11894599; doi:10.1021/acs.inorgchem.4c03214)

Supporting Information

Diverse and Selective Metal-Ligand Cooperative Routes for Activating Non-Functionalized Ketones

Carlos Ferrer-Bru, Joaquina Ferrer, Vincenzo Passarelli,* Fernando J. Lahoz, Pilar García-Orduña, and Daniel Carmona**

Instituto de Síntesis Química y Catálisis Homogénea (ISQCH), CSIC - Universidad de Zaragoza, Departamento de Química Inorgánica, Pedro Cerbuna 12, 50009 Zaragoza, Spain, E-mail: dcarmona@unizar.es (D. C.), jfecer@unizar.es (J. F.), passarel@unizar.es (V. P.)

Table of Contents

1. Composition of equilibrium mixtures and isolated solid (complexes 3-8) S2

2. Selected NMR spectra of complexes 4 and 8 S3

3. Relevant NOE interactions for the assignment of *Z* and *E* isomers S5

4. Thermodynamic data for the equilibria 1 ⇌ 7*Z* and 1 ⇌ 7*E* S6

5. NMR spectra of compounds 9*Z*, 10, 11, and 12 S8

6. ^{1}H and ^{19}F{^{1}H} NMR spectra for the equilibrium 11 ⇌ 11a S14

7. Selected NOE interactions for the major isomer of compounds 9 and 10 S15

8. Selected NOE interactions for compounds 11 and 12 S16

9. Selected mass spectra for the reaction of complex 1 with CF_3COR ketones S17

10. DFT calculations S19

1. Composition of equilibrium mixtures and isolated solid (complexes 3-8)

Table S1. Composition of the equilibrium mixtures of complexes **1**/**3**, **2**/**4**, **1**/**5**, **2**/**6**, **1**/**7** and **2**/**8** obtained at different temperatures

Entry	Metal	Ketone	Product	React. Temp. (K)	Time (h)	Conversion	*Z*/*E* molar ratio
1	Rh	CH_3COCH_3	**3**	303	48	45/55 (**1**/**3**)	
2				313	24	56/44	
3				323	24	68/32	
4				333	15	73/27	
5	Ir	CH_3COCH_3	**4**	333	48	14/86 (**2**/**4**)	69/31
6				363	2	35/65	71/29
7	Rh	CH_3COPh	**5**	313	48	46/54 (**1**/**5**)	
8				333	16	60/40	
9				363	2	68/32	
10	Ir	CH_3COPh	**6**	333	22	17/83 (**2**/**6**)	60/40
11				363	15	31/69	61/39
12				393	3	56/44	61/39
13	Rh	CH_3COCF_3	**7**	273	*	2/98 (**1**/**7**)	24.5/75.5
14				283	*	4/96	25/75
15				293	*	7/93	24/76
16				303	*	12/88	28/72
17				313	*	20/80	27.5/72.5
18	Ir	CH_3COCF_3	**8**	281	96	1/99 (**2**/**8**)	10/90

* Equilibrium was reached after 30 min of reaction

Table S2. Composition of the isolated solid

Entry	Metal	Ketone	Product	Reac. Temp. (K)	Solid molar ratio
1	Rh	CH_3COCH_3	**3**	303	48/20/32 (**1**/**3***Z*/**3***E*)
2	Ir	CH_3COCH_3	**4**	333	35/45/20 (**2**/**4***Z*/**4***E*)
3	Rh	CH_3COPh	**5**	313	44/34/22 (**1**/**5***Z*/**5***E*)
4	Ir	CH_3COPh	**6**	333	18/82 (**2**/**6***Z*)
5	Rh	CH_3COCF_3	**7**	273	3/24/73 (**1**/**7***Z*/**7***E*)
6	Ir	CH_3COCF_3	**8**	281	10/90 (**8***Z*/**8***E*)

2. Selected NMR spectra of complexes 4 and 8

Figure S1. ^{1}H NMR spectrum (THF-d_8, RT) of a **2**/**4***Z*/**4***E* mixture in 35/45/20 molar ratio, respectively. The peaks assigned to complex **2** are marked with a filled circle

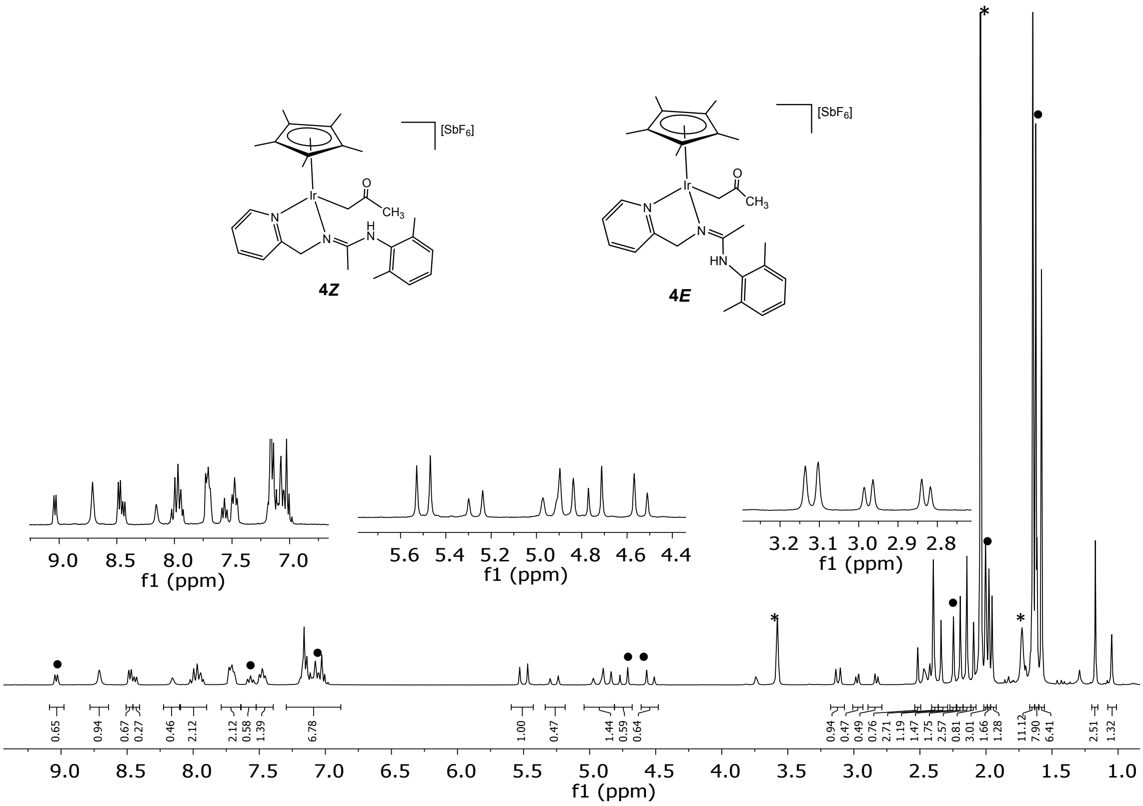

Figure S2. ^{13}C{^{1}H} APT spectrum (THF-d_8, RT) of a **2**/**4***Z*/**4***E* mixture in 35/45/20 molar ratio, respectively. The peaks assigned to complex **2** are marked with a filled circle

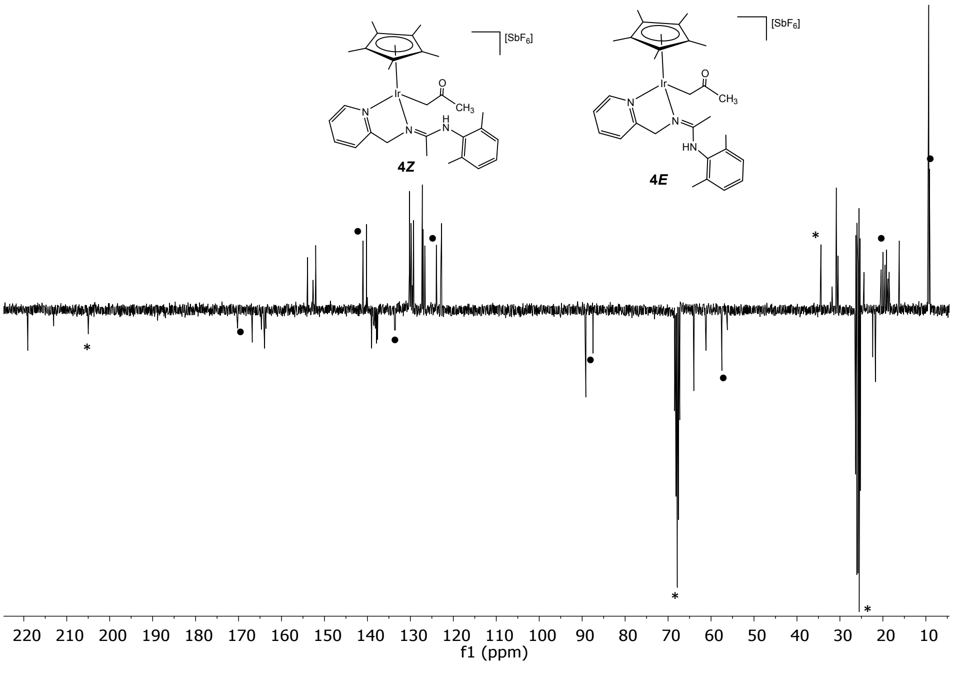

Figure S3. ^{1}H NMR spectrum (CD_2Cl_2, RT) of an **8***Z*/**8***E* mixture in 10/90 molar ratio, respectively. The peaks assigned to complex **8***Z* are marked with a filled circle

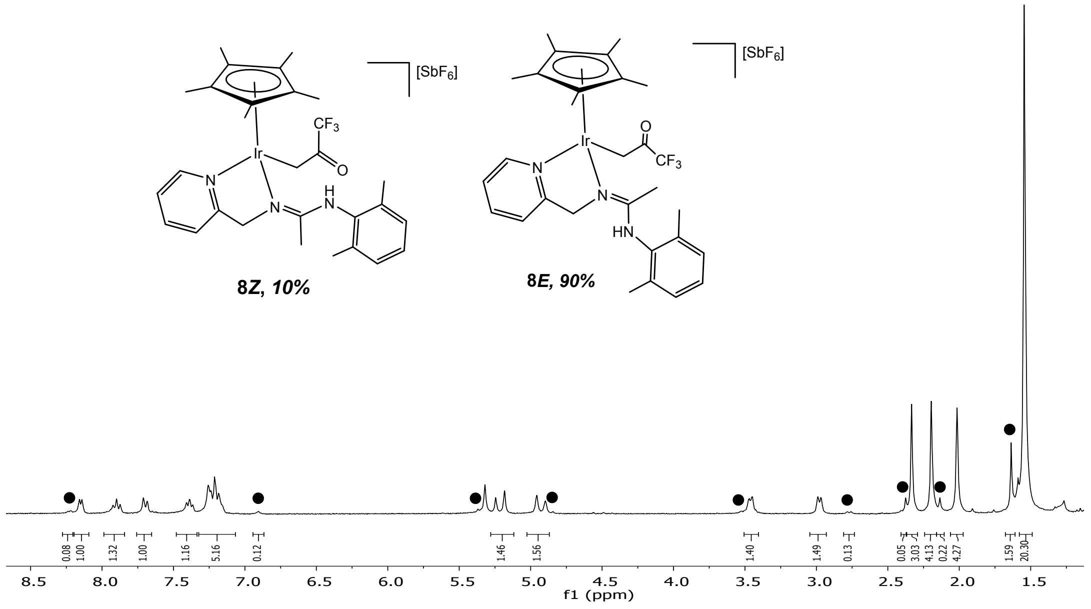

Figure S4. $^{19}F\{^{1}H\}$ NMR spectrum (CD_2Cl_2, RT) of an **8***Z*/**8***E* mixture in 10/90 molar ratio, respectively

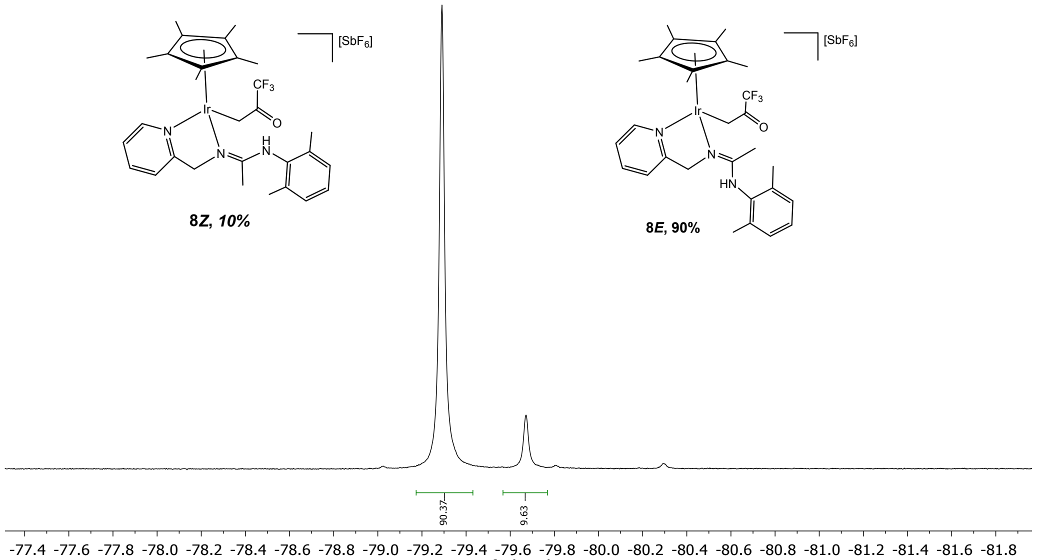

Figure S5. $^{13}C\{^{1}H\}$ APT spectrum (CD_2Cl_2, RT) of an **8***Z*/**8***E* mixture in 10/90 molar ratio, respectively

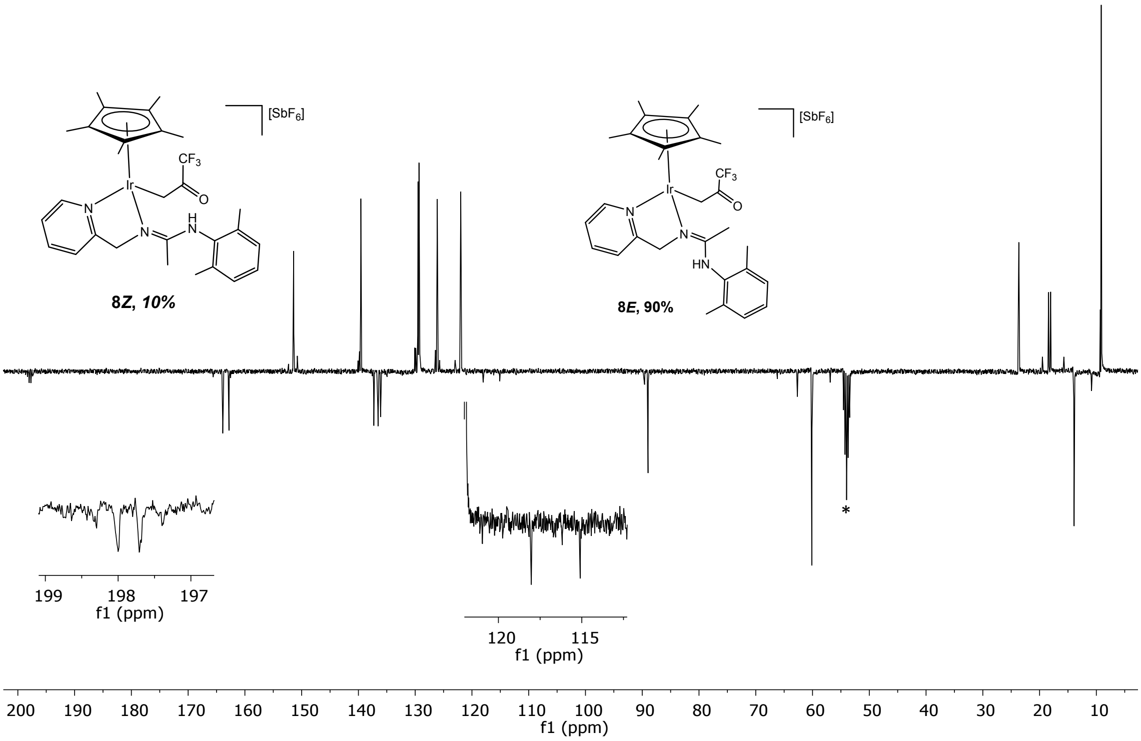

3. Relevant NOE interactions for the assignment of *Z* and *E* isomers

Figure S6. NOESY fragments (CD_2Cl_2, RT) of a mixture of **4***Z* and **4***E*, showing the NH (left) and MeCN (right) contacts

Compound 4

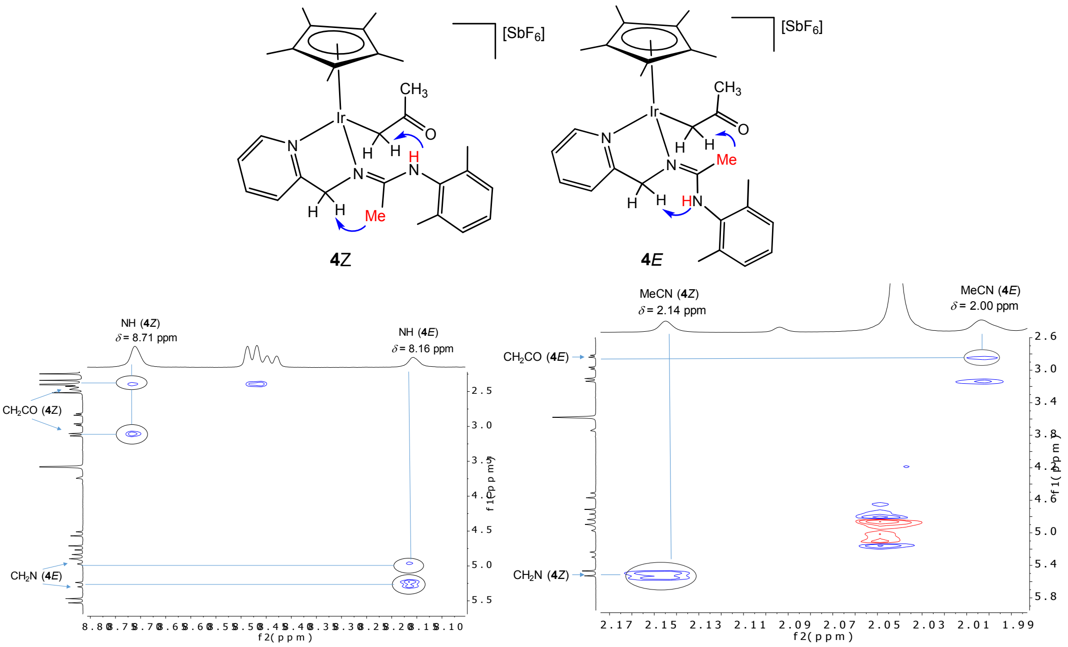

Figure S7. NOESY fragments (CD_2Cl_2, RT) of a mixture of **8***Z* and **8***E*, showing the NH (left) and MeCN (right) contacts

*Compound **8***

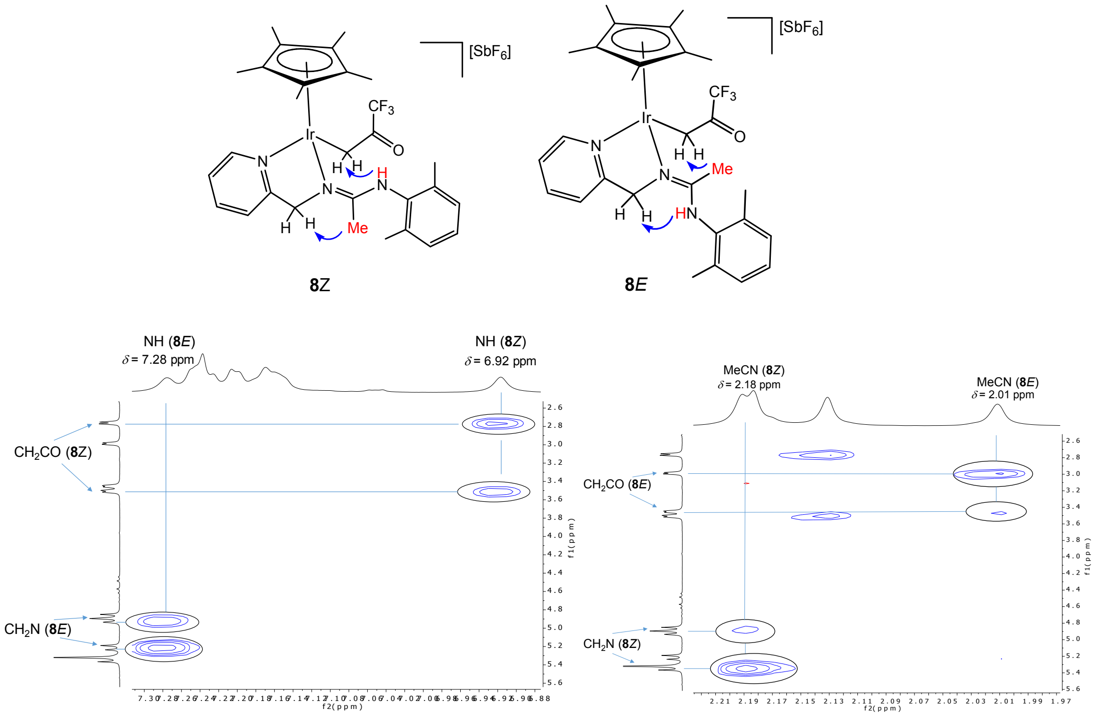

4. Thermodynamic data for the equilibria 1 ⇆ 7*Z* and 1 ⇆ 7*E*

Table S3. Values of K_{obs} for the equilibria **1** ⇆ **7***Z* and **1** ⇆ **7***E* at different temperatures. Complex **1**, 0.014 mmol; CH_3COCF_3, 250.8 mmol in 0.20 mL/0.20 mL of CH_3COCF_3/CD_2Cl_2.

T (K)	% 1	% 7Z	% 7*E*	K_{obs} (7*Z*)	ln K_{obs} (7*Z*)	K_{obs} (7*E*)	ln K_{obs} (7*E*)	1/T (1/K)
273	2.0	24.5	73.5	12.3	2.51	36.8	3.60	$3.60 \cdot 10^{-3}$
283	3.9	24.0	72.1	6.15	1.82	18.5	2.92	$3.53 \cdot 10^{-3}$
293	7.5	22.2	70.3	2.96	1.09	9.37	2.24	$3.41 \cdot 10^{-3}$
303	12.8	24.9	62.3	1.95	0.67	4.87	1.58	$3.30 \cdot 10^{-3}$
313	19.8	22.5	57.7	1.14	0.13	2.91	1.07	$3.20 \cdot 10^{-3}$

Figure S8. Calculated values of ΔH°, ΔS° and ΔG° for the equilibria **1** ⇆ **7***Z*

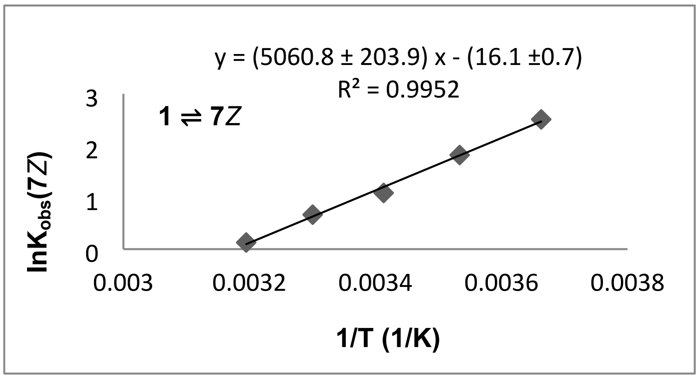

ΔH°	ΔS°	ΔG°
−10.06 ± 0.41 Kcal·mol^{-1}	−31.94 ± 1.39 cal·mol^{-1}	−0.54 ± 0.83 Kcal·mol^{-1}

Figure S9. Calculated values of ΔH°, ΔS° and ΔG° for the equilibria **1** ⇆ **7***E*

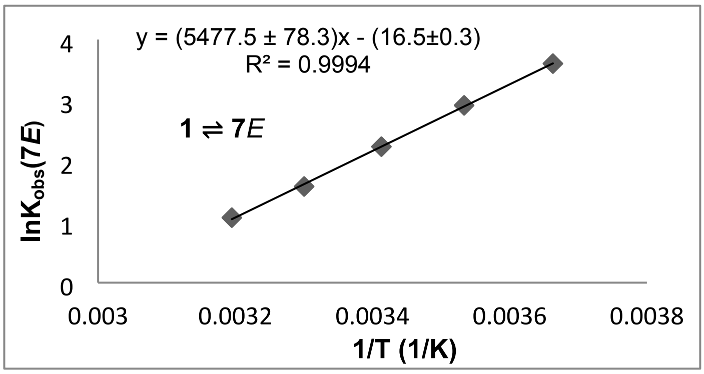

ΔH°	ΔS°	ΔG°
−10.88 ± 0.16 Kcal·mol^{-1}	−32.70 ± 0.60 cal·mol^{-1}	−1.14 ± 0.34 Kcal·mol^{-1}

5. NMR spectra of compounds 9*Z*, 10, 11, and 12

Compound 9Z

Figure S10. ^{1}H NMR (CD_2Cl_2, RT)

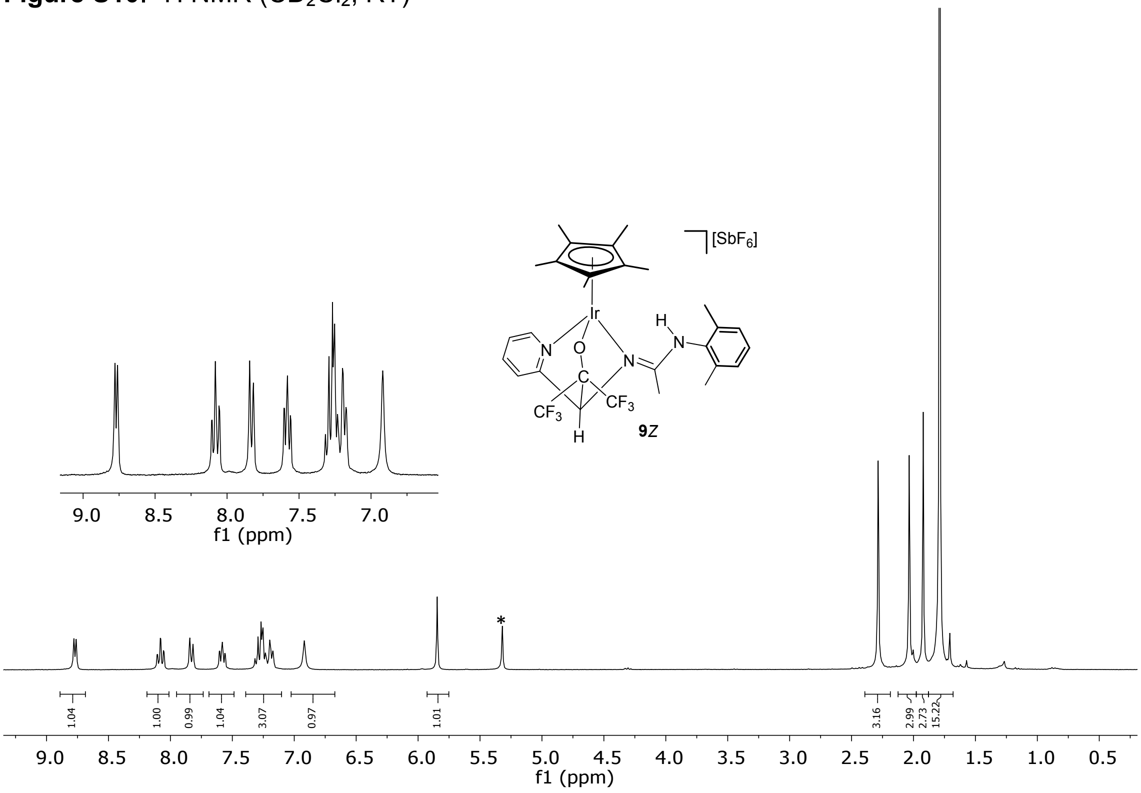

Figure S11. $^{19}F\{^{1}H\}$ NMR (CD_2Cl_2, RT)

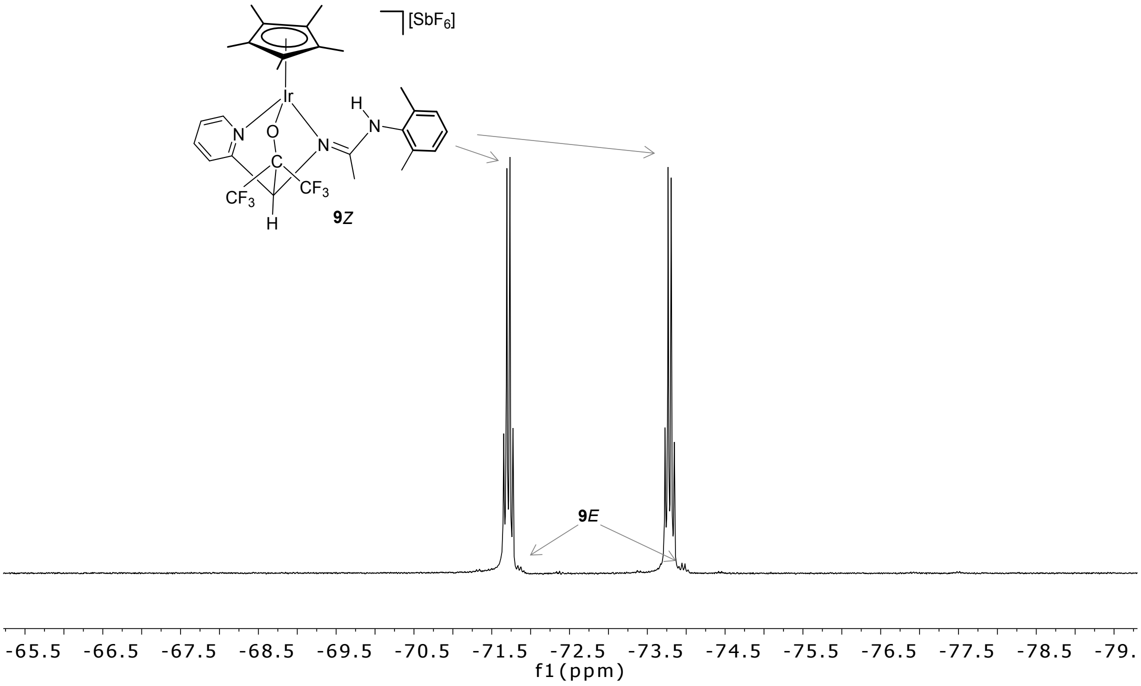

Figure S12. $^{13}C\{^{1}H\}$ APT (CD_2Cl_2, RT)

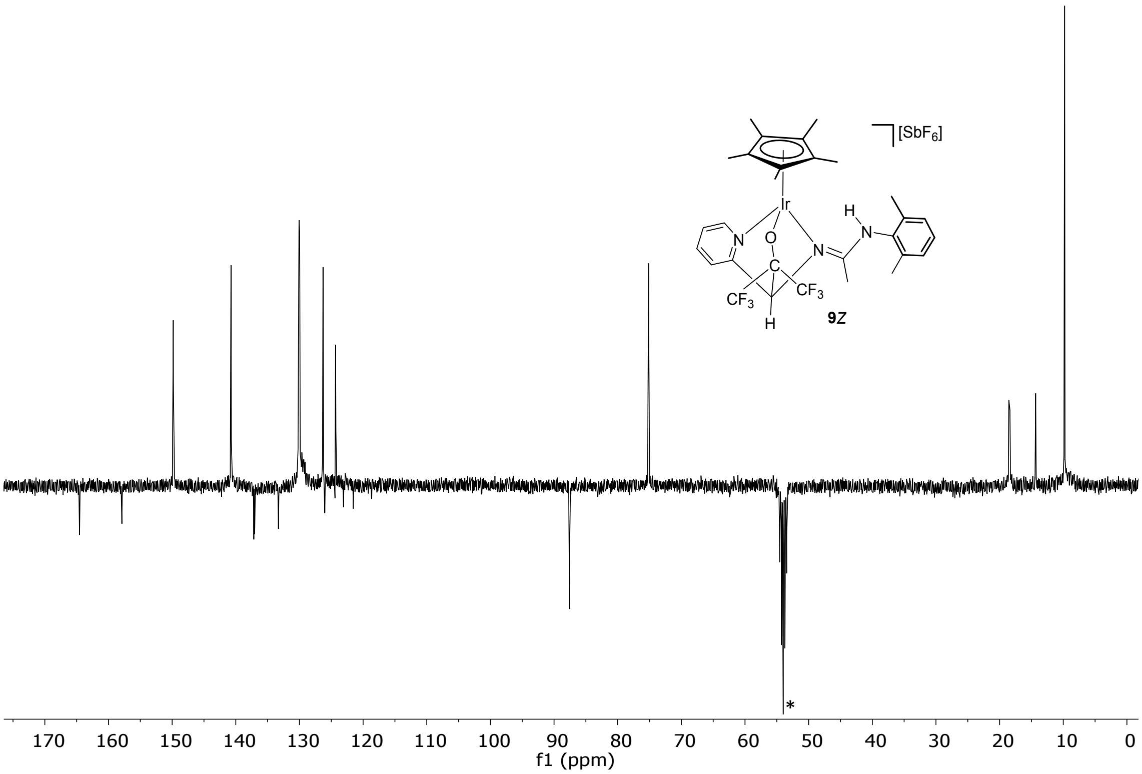

*Compound **10***

Figure S13. ^{1}H NMR (CD_2Cl_2, RT)

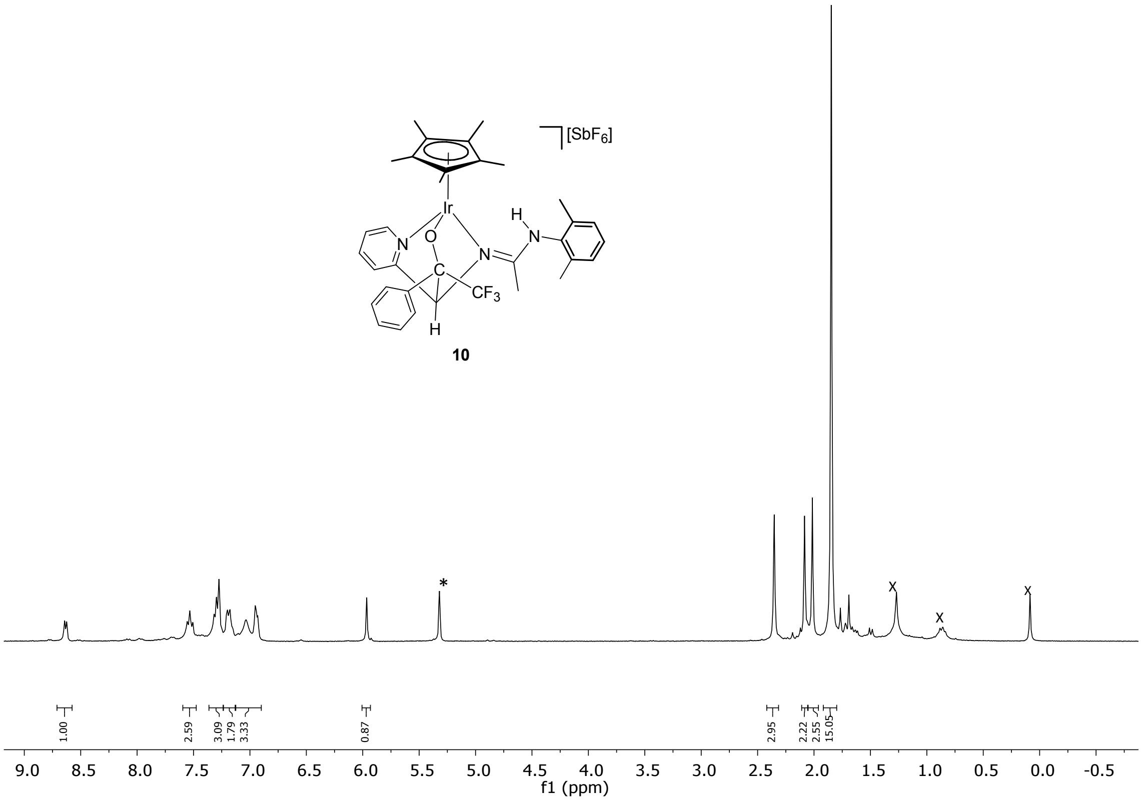

Figure S14. $^{19}F\{^1H\}$ NMR (CD_2Cl_2, RT)

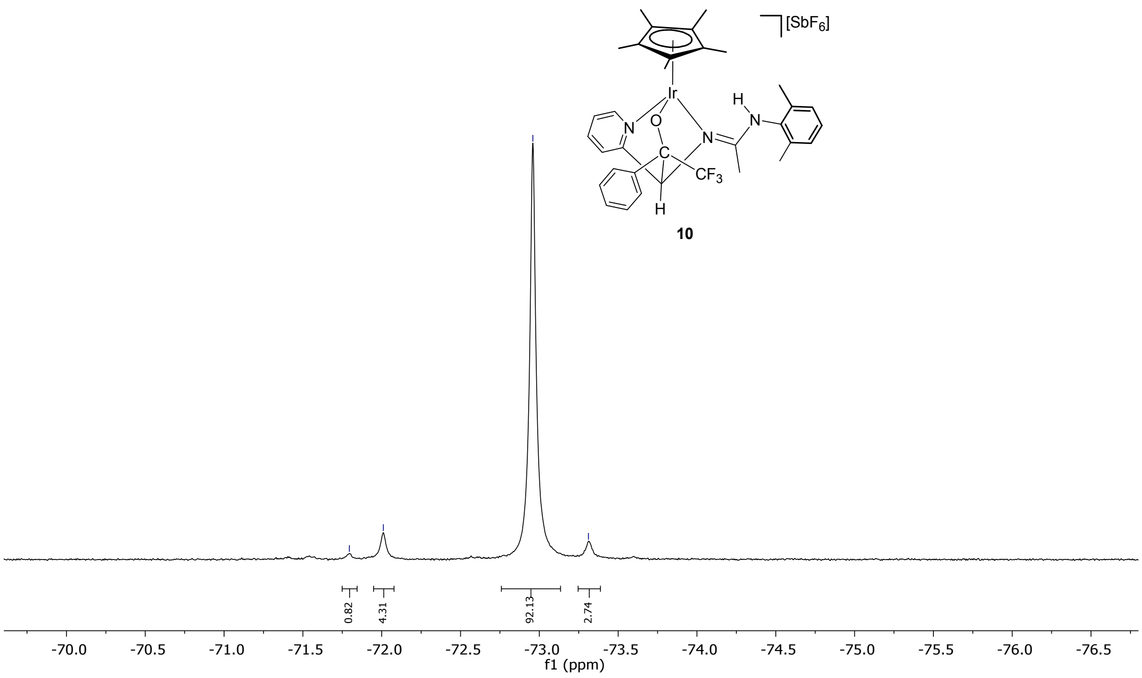

Figure S15. $^{13}C\{^1H\}$ APT (CD_2Cl_2, RT)

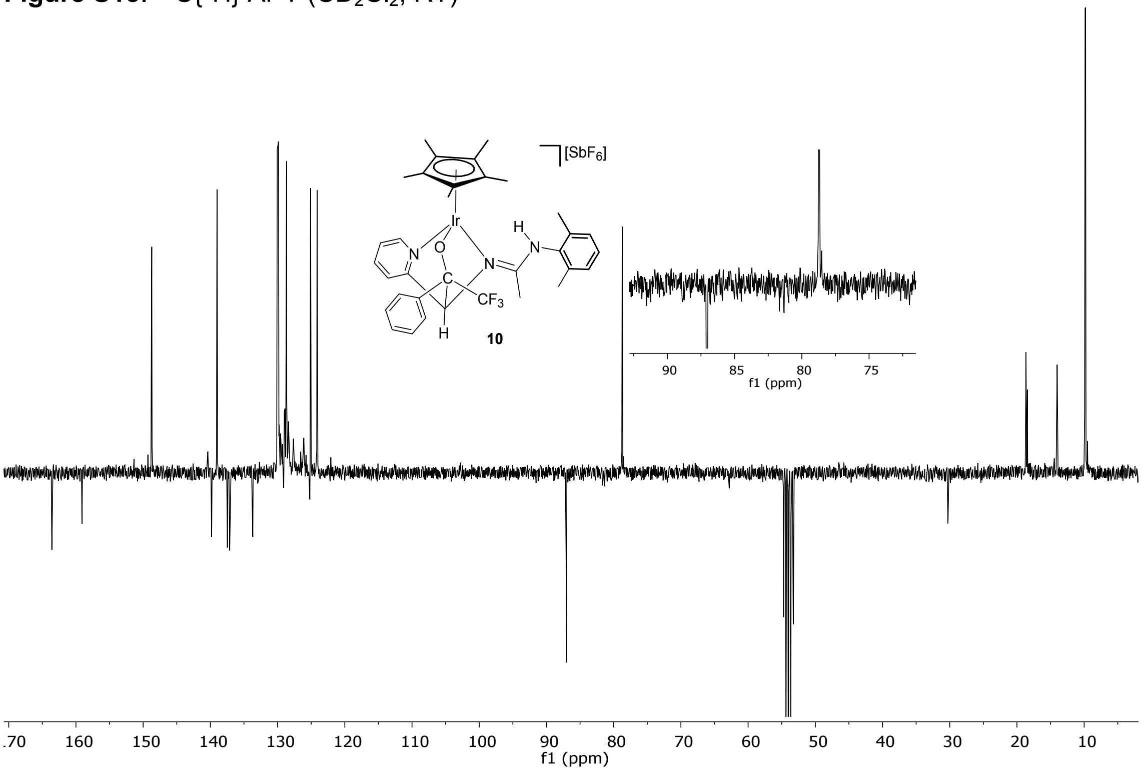

*Compound **11***

Figure S16. ^{1}H NMR (CD_2Cl_2, RT)

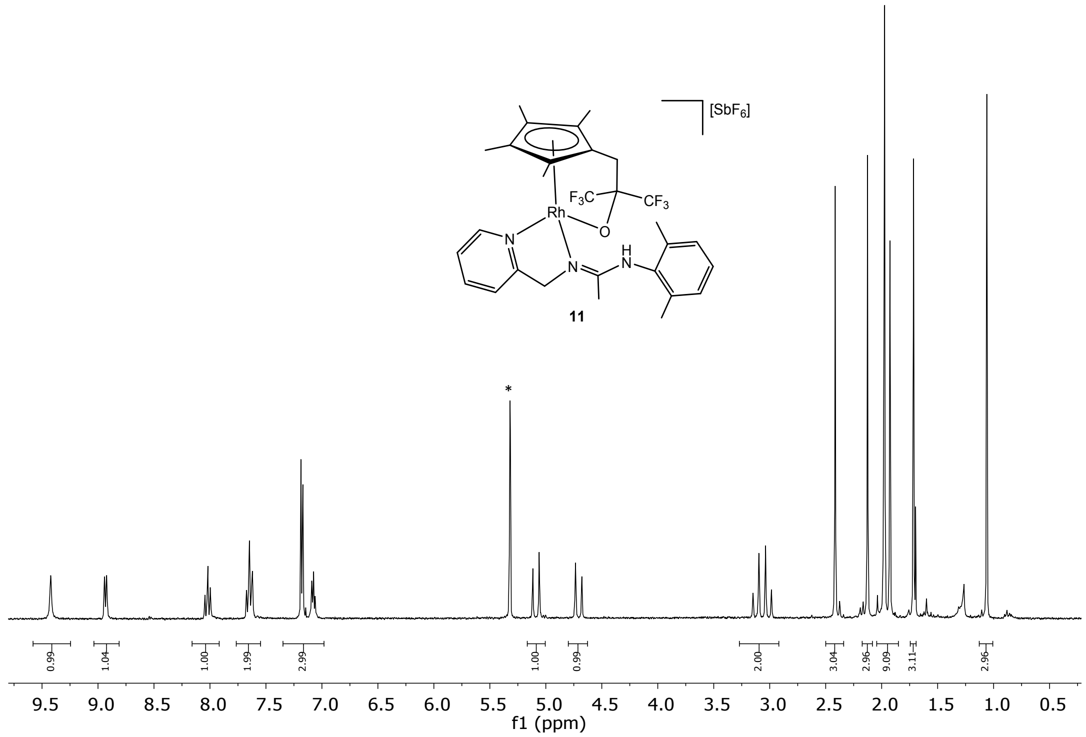

Figure S17. $^{19}F\{^{1}H\}$ NMR (CD_2Cl_2, RT)

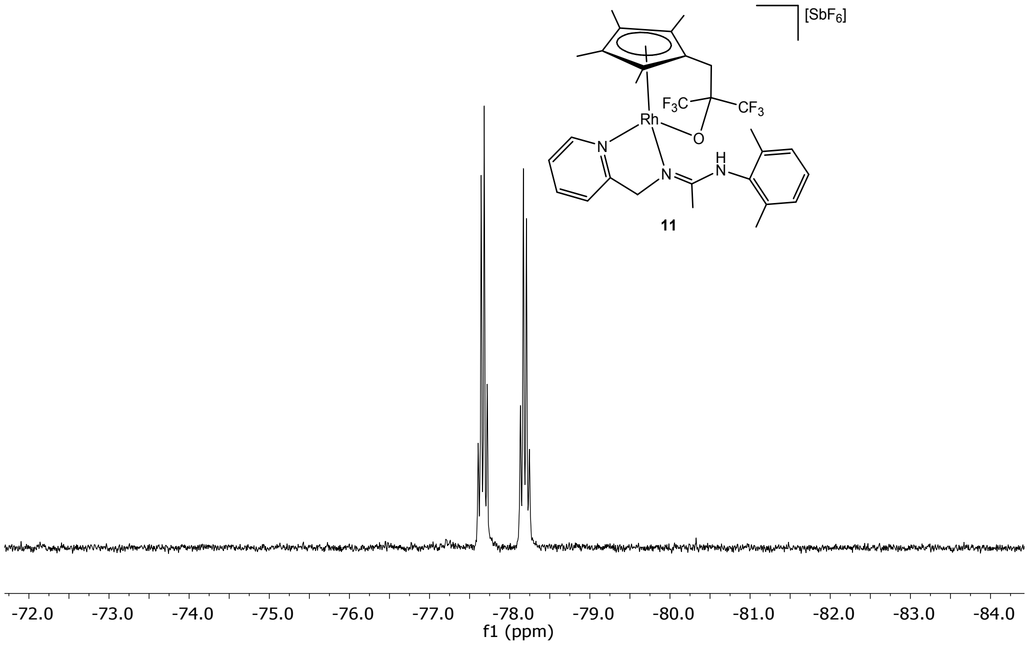

Figure S18. $^{13}C\{^1H\}$-APT (CD_2Cl_2, RT)

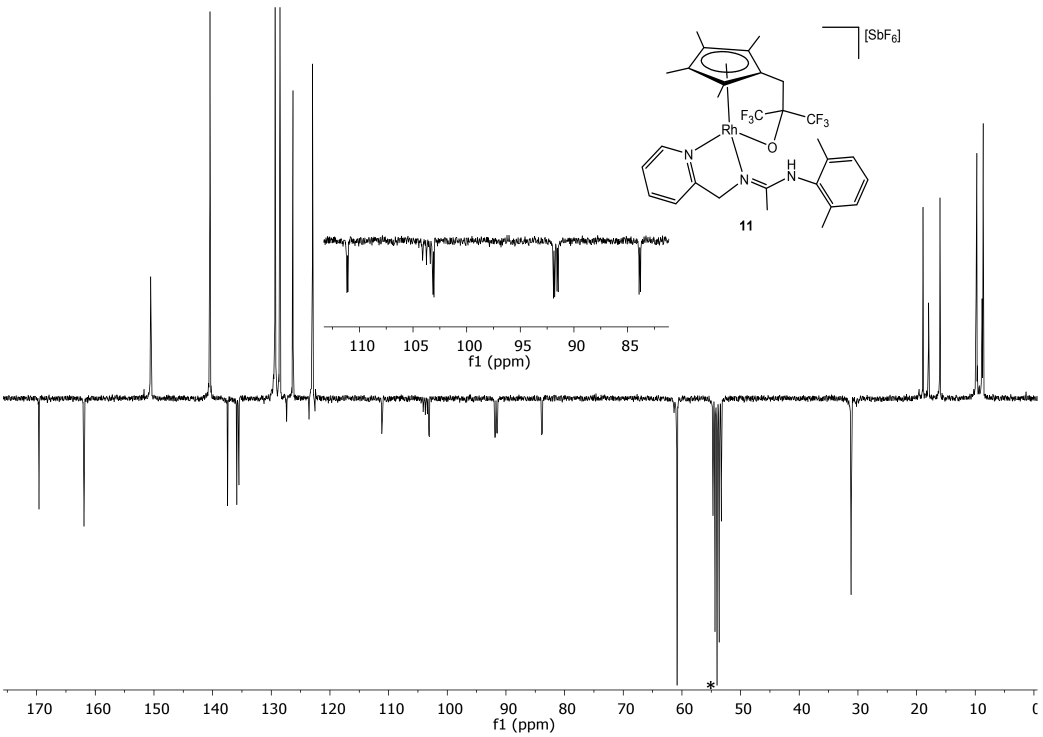

Compound ***12***

Figure S19. ^1H NMR (CD_2Cl_2, RT)

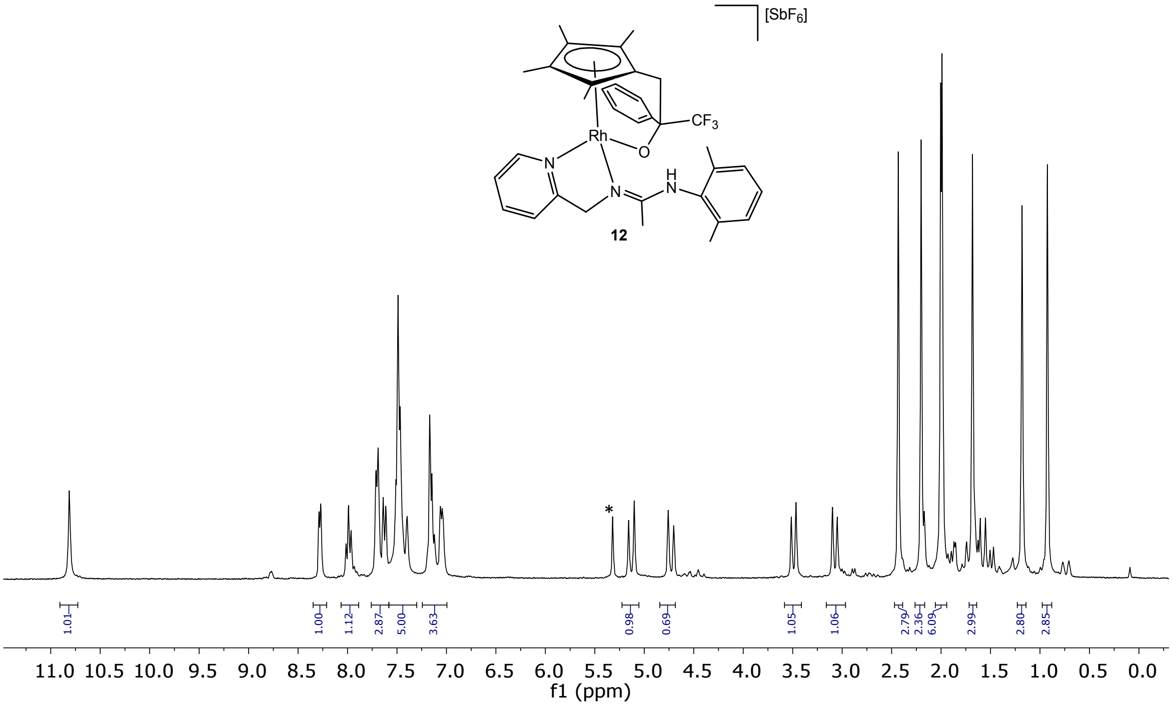

Figure S20. $^{19}F\{^{1}H\}$ NMR (CD_2Cl_2, RT)

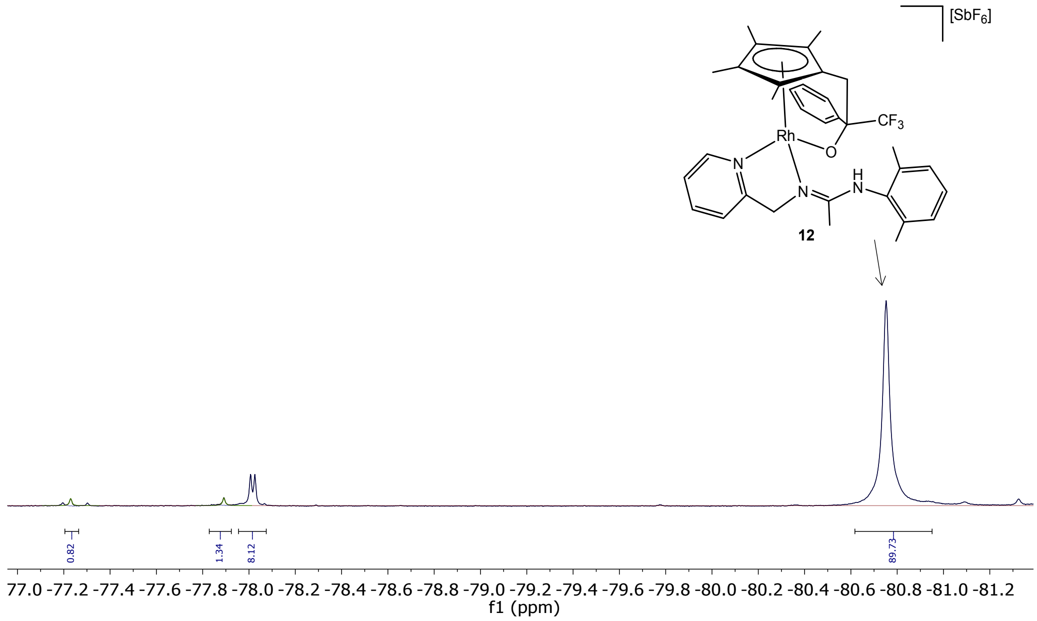

Figure S21. $^{13}C\{^{1}H\}$ APT (CD_2Cl_2, RT)

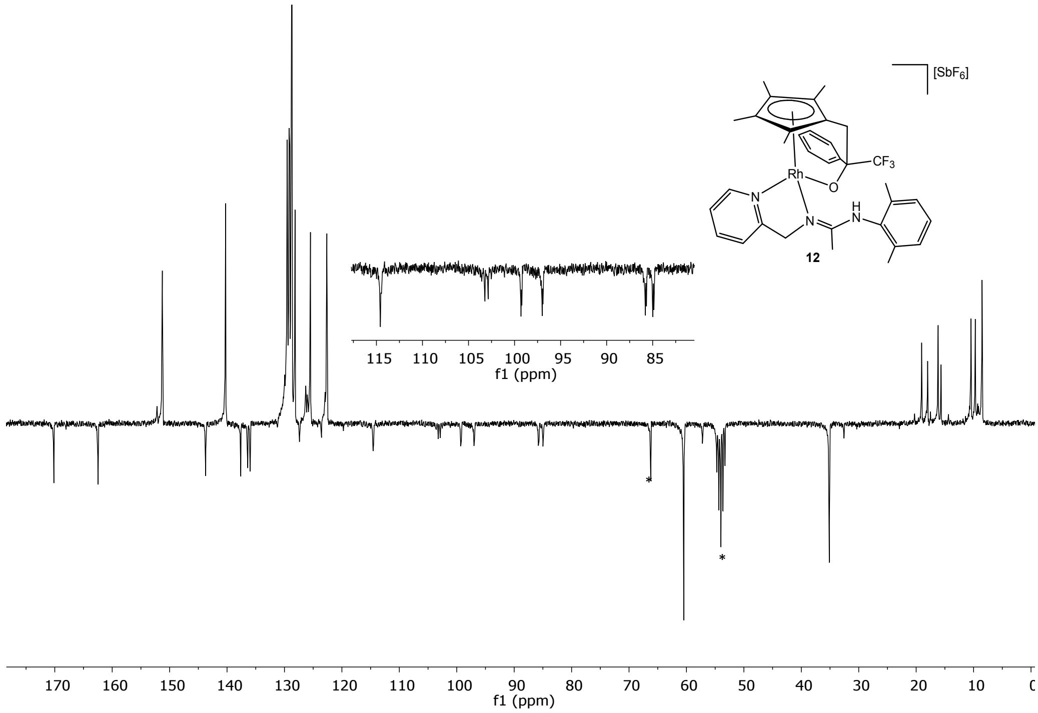

6. ^{1}H and $^{19}F\{^{1}H\}$ NMR spectra for the equilibrium 11 ⇌ 11a

Figure S22.^{1}H NMR spectrum (THF-d_8, 273 K) showing the equilibrium **11** ⇌ **11a**. The peaks assigned to complex **11** are marked with a filled circle

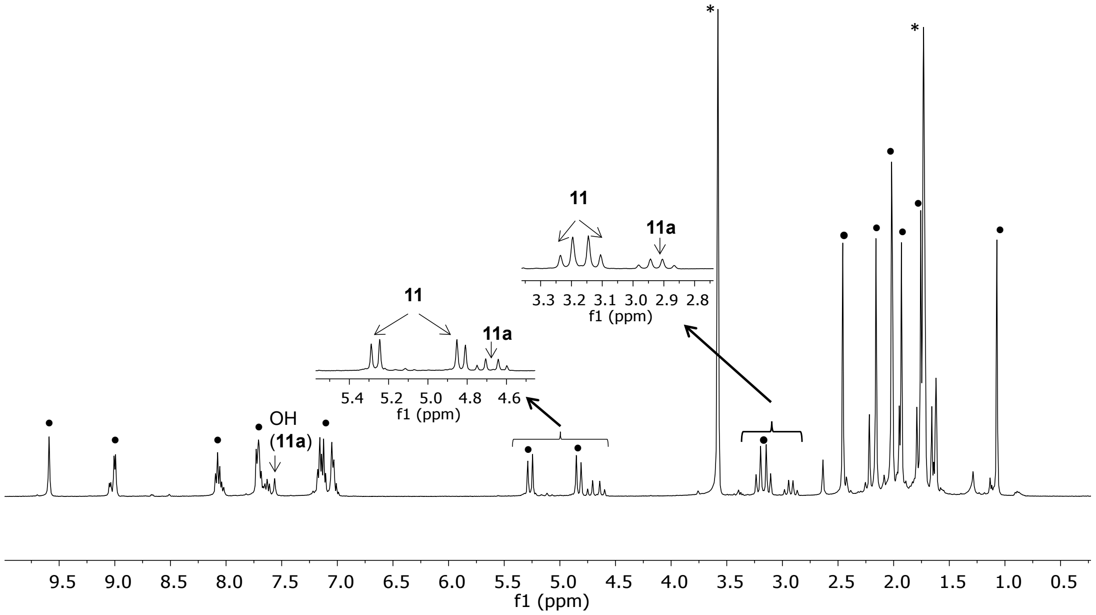

Figure S23. $^{19}F\{^{1}H\}$ NMR spectrum (THF-d_8, 273 K) showing the equilibrium **11** ⇌ **11a**

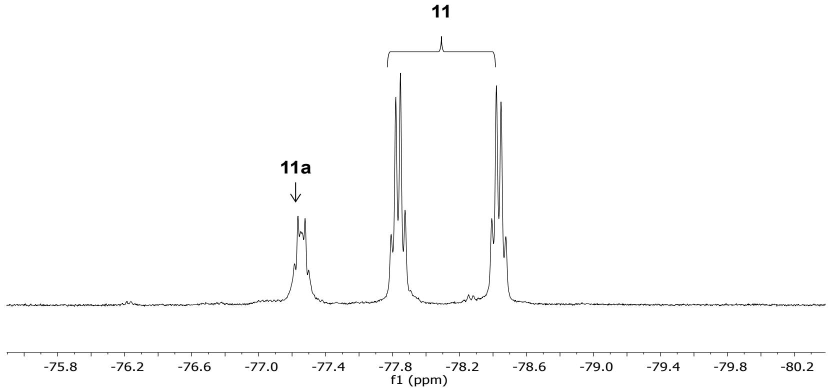

7. Selected NOE interactions for the major isomer of compounds 9 and 10

Figure S24. Selected fragments of the NOEDIFF spectra (CD_2Cl_2, RT) of **9***Z*: irradiation of NH (left) and MeCN protons (right)

Compound ***9Z***

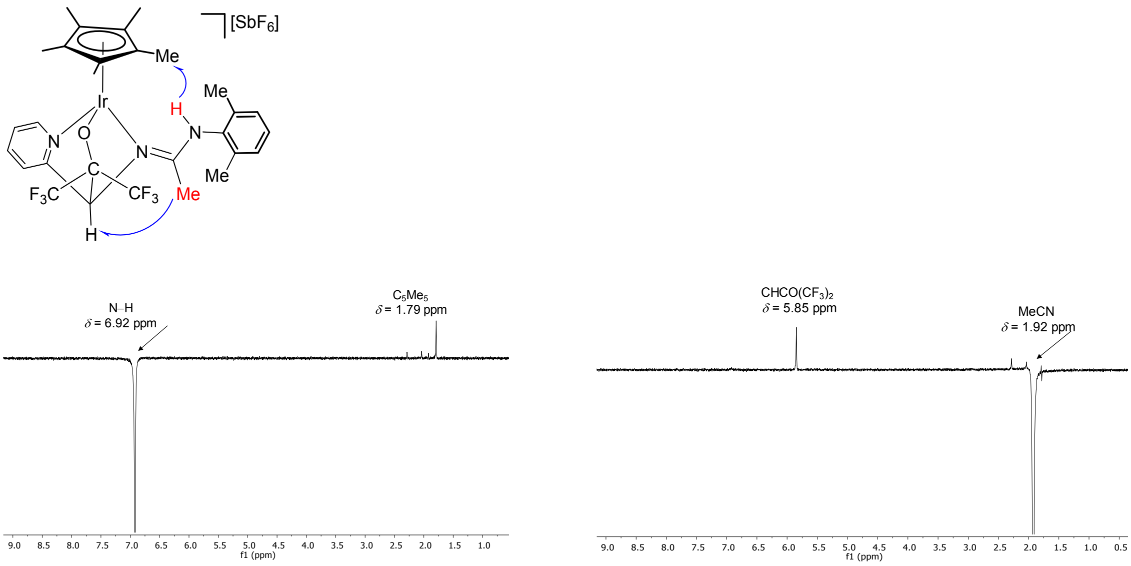

Figure S25. NOESY fragments (CD_2Cl_2, RT) of **10** showing NH (left) and MeCN (right) contacts

Compound ***10***

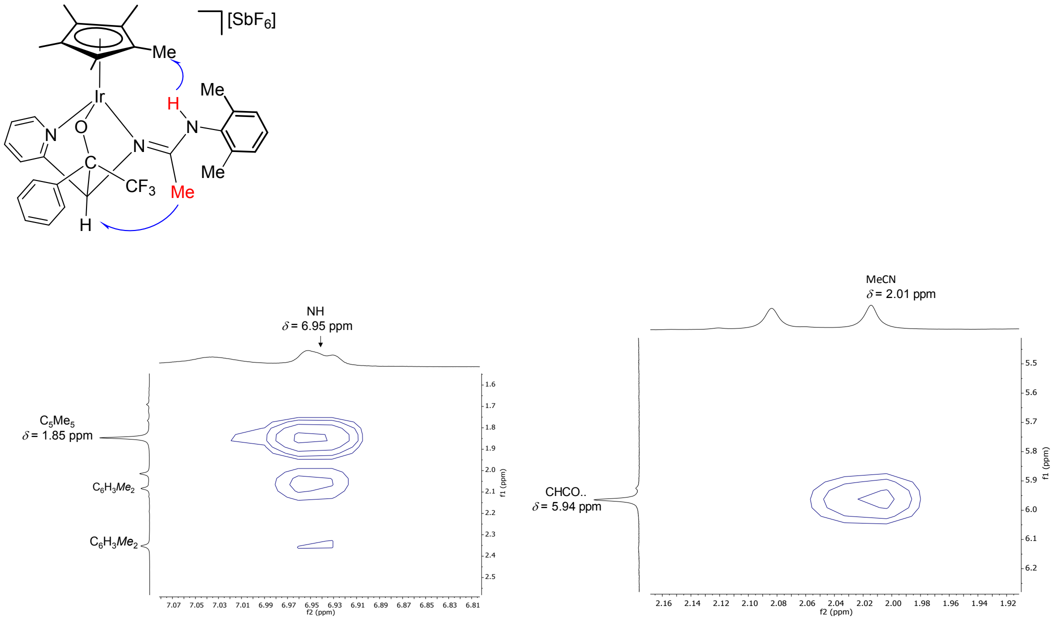

8. Selected NOE interactions for compounds 11 and 12

Figure S26. NOESY fragments (CD_2Cl_2, RT) of **11** showing NH (left) and MeCN (right) contacts

*Compound **11***

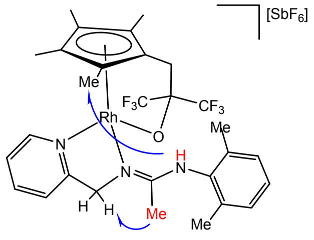

11

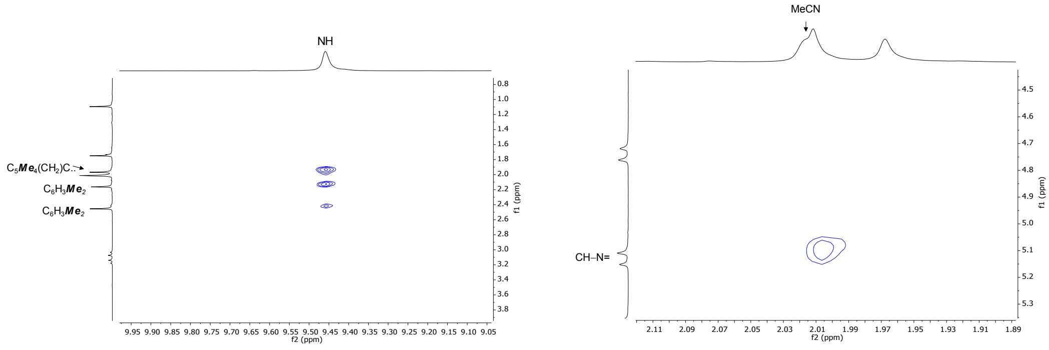

Figure S27. NOE fragments (CD_2Cl_2, RT) of **12**, showing NH (left) and MeCN (right) contacts

*Compound **12***

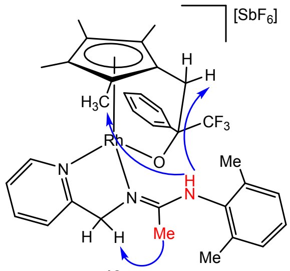

12

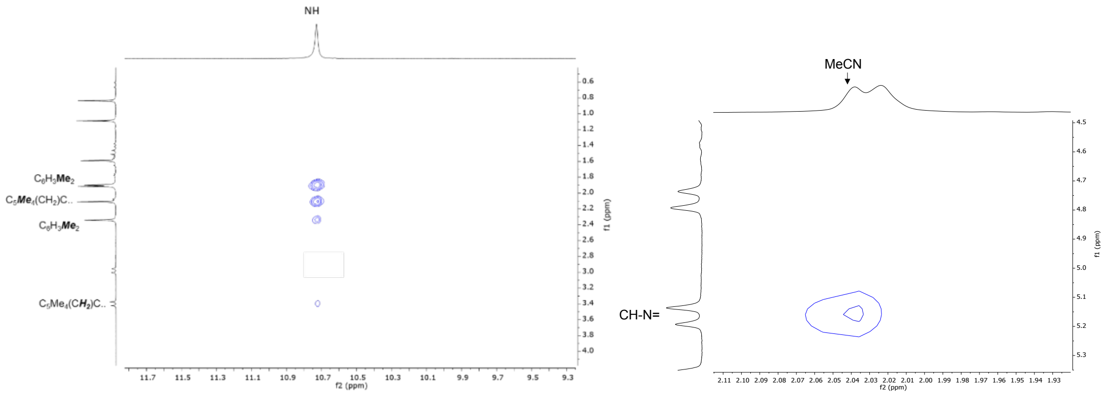

9. Selected mass spectra for the reaction of complex 1 with CF_3COR ketones

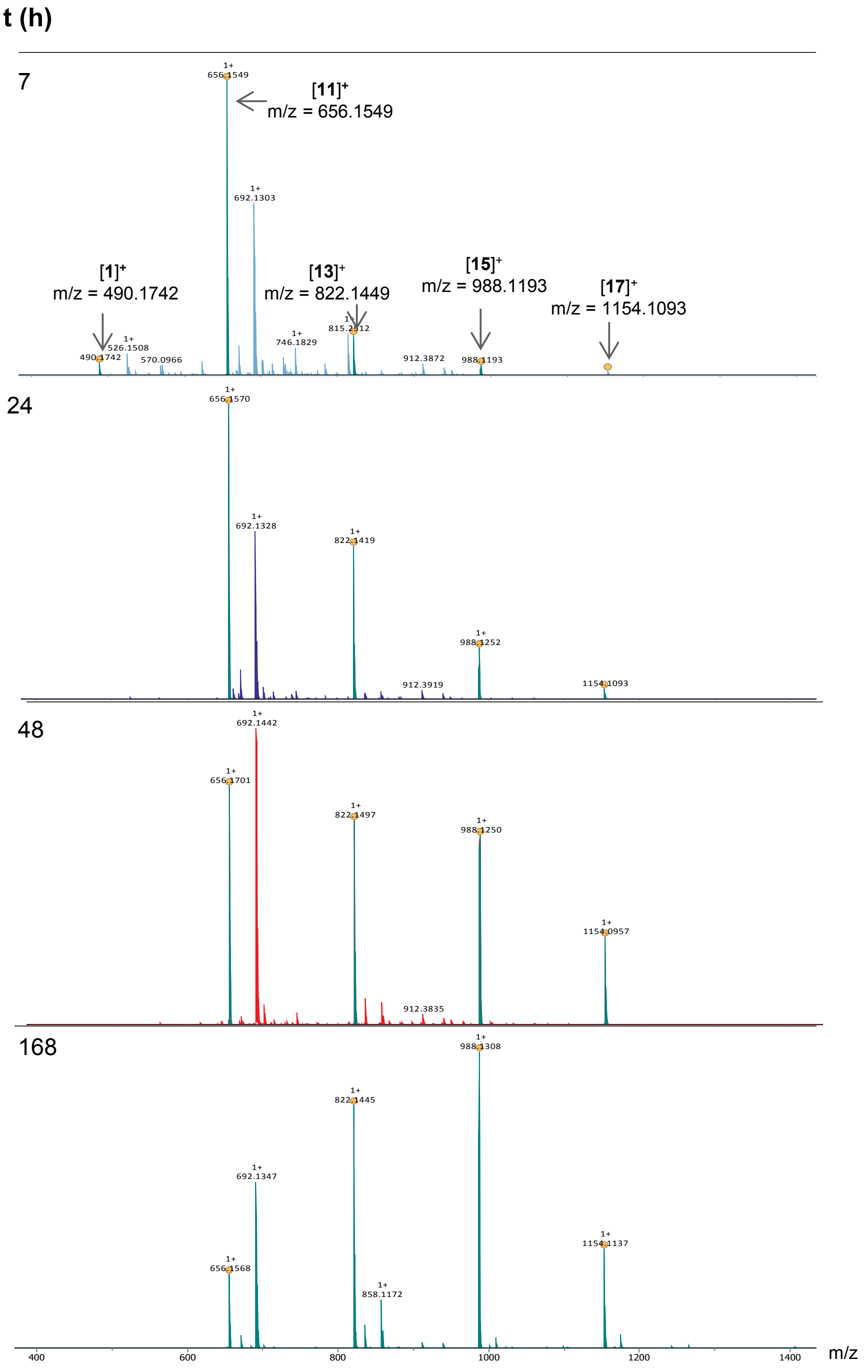

Figure S28. Evolution of the mass spectra of the reaction of complex **1** (0.010 mmol) with $(CF_3)_2CO$ (0.100 mmol) in THF at 333 K

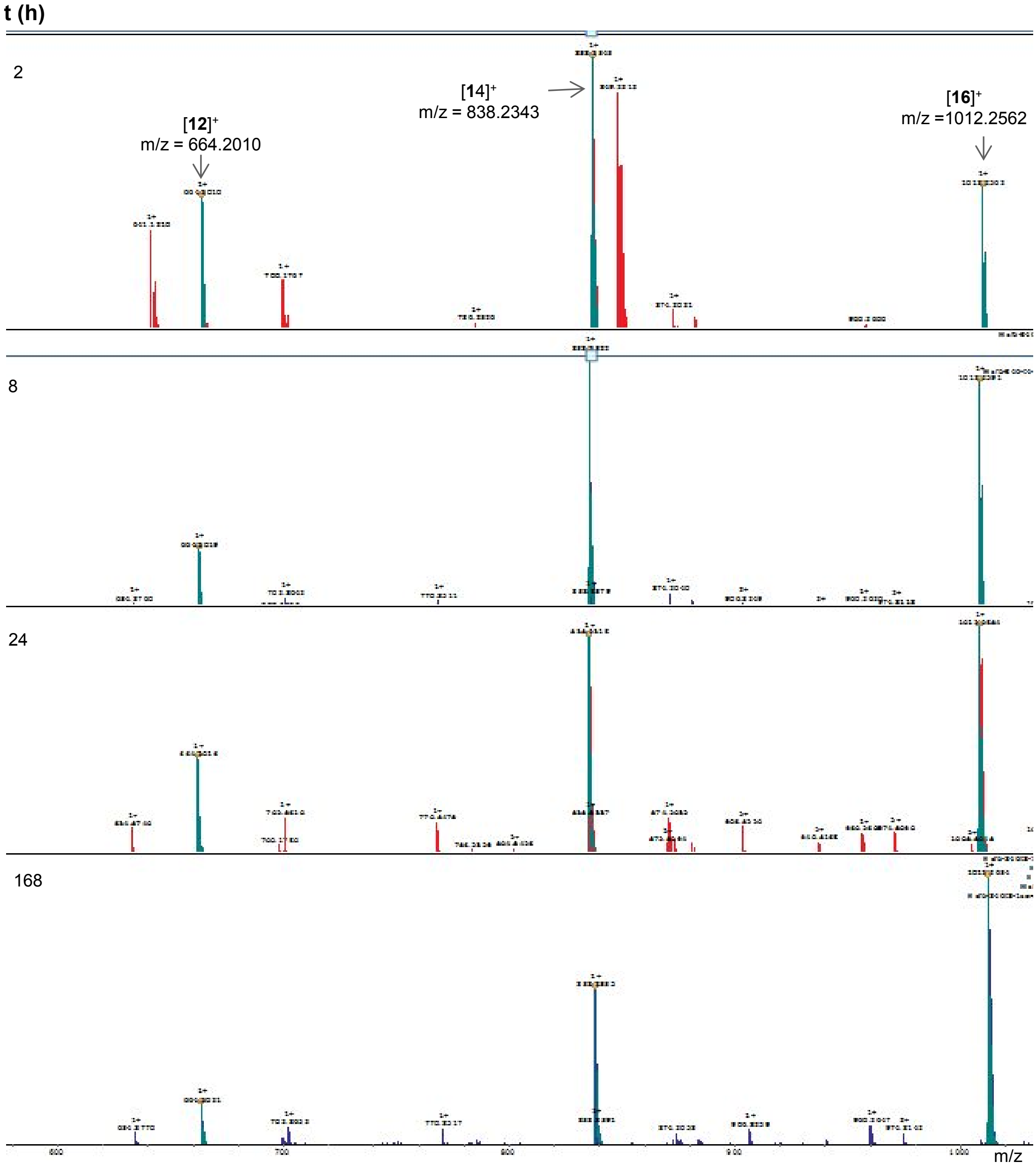

Figure S29. Evolution of the mass spectra of the reaction of complex **1** (0.010 mmol) with CF_3COPh (0.4 mL) at 333 K

10. DFT calculations

Table S4. Energy and Gibbs free energy values (Ha) of calculated structures

	E (B97D3/def2svp, Ha)	G (B97D3/def2svp, Ha)
1 (VPC215_a)	-1284.098302	-1283.637741
2 (VPC215_b)	-1277.906709	-1277.446136
	-1278.756905*	-1278.296332*
7E (VPC215_i4)	-1774.374971	-1773.859444
7Z (VPC215_i)	-1774.377423	-1773.862231
8E (VPC215_j4)	-1768.182966	-1767.66888
8Z (VPC215_j)	-1768.186014	-1767.671086
9' (VPC215_n2)	-2065.531546	-2065.042166
	-2067.426427*	-2066.937047*
9 (VPC215_n)	-2065.538265	-2065.048052
	-2067.432713*	-2066.942500*
10 (VPC215_n6)	-1959.667423	-1959.105262
10a (VPC215_n5c)	-1959.661815	-1959.097617
10b (VPC215_n5b)	-1959.669122	-1959.102131
10c (VPC215_n6b)	-1959.661663	-1959.098492
11 (VPC215_k)	-2071.730145	-2071.23488
11a (VPC220_g)	-2071.720454	-2071.22797
13 (VPC220_a)	-2859.350799	-2858.826468
13a (VPC220_a2)	-2859.337587	-2858.811966
CF3COCF3 (VPC215_o)	-787.584363	-787.582793
CF3COPh (VPC221_e)	-681.728157	-681.652103
CH2COHCF3 (VPC215_q)	-490.232673	-490.20331
CH3COCF3 (VPC215_p)	-490.249594	-490.222994
I-Ir (VPC215_d)	-1277.893331	-1277.434065
I-Rh (VPC215_c)	-1284.080532	-1283.622104
II-Ir (VPC217_b)	-1768.155256	-1767.640992
III-Ir (VPC217_b2)	-1768.179982	-1767.6651
III-Rh (VPC217_a)	-1774.374624	-1773.859171
IV-Ir (VPC215_d2)	-1277.891469	-1277.432989
IV-Rh (VPC215_c2)	-1284.07937	-1283.621679
IX-Ir (VPC219_c)	-1959.644087	-1959.084933
V-Ir (VPC215_r)	-1277.882987	-1277.425423
V-Rh (VPC215_r2)	-1284.077677	-1283.619261
VI-Ir (VPC219_a)	-2065.493228	-2065.012604
VII-Ir (VPC218_i2)	-2065.519267	-2065.027469
VIII (VPC218_g)	-2065.509581	-2065.016897
X-Ir (VPC219_c2)	-1959.64385	-1959.083141
XI-Ir (VPC219_d)	-1959.649512	-1959.084337
XII-Ir (VPC219_d2)	-1959.650157	-1959.084484
XIII-Rh (VPC215_e)	-1284.087707	-1283.626999
XIV-Rh (VPC216_a3)	-2071.693008	-2071.208676
XV-Rh (VPC220_c)	-2071.704051	-2071.214008
XVI-Rh (VPC220_e)	-2071.707891	-2071.215442
XVII-Rh (VPC220_e2)	-2071.70748	-2071.215002
XVIII-Rh (VPC220_b)	-2859.348235	-2858.824947
XIX-Rh (VPC220_b2)	-2859.340032	-2858.816977
XX-Ir (VPC215_l)	-2065.535427	-2065.041604
	-2067.425173*	-2066.931350*
TS_I-XIII_Ir (VPC215_h)	-1277.865918	-1277.40908
	-1278.711160*	-1278.254322*
TS_I-XIII_Rh (VPC215_g)	-1284.058584	-1283.602115
TS_IV-V_Ir (VPC215_s2)	-1277.857021	-1277.403002
	-1278.708011*	-1278.253992*
TS_IV-V_Rh (VPC215_s3)	-1284.0458	-1283.591517
TS_XV-XVI_Rh (VPC220_d)	-2071.681224	-2071.193756
TS_XV-XVII_Rh (VPC220_d2)	-2071.675472	-2071.188836

* M06/def2tzvp//B97D3/def2svp

Figure S30. Energy profile for (left) **VI**-Ir → **VII**-Ir and (right) **9'** → **9** (B97D3/def2svp; E, kcal·mol^{-1}; C···C, Å; C−N=C−C, º)

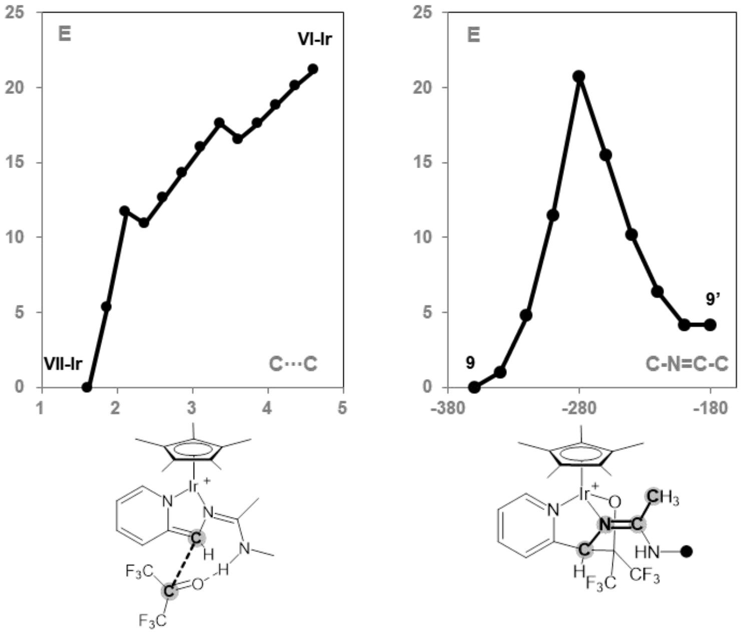

Figure S31. Energy profile for (left) **IX**-Ir → **XI**-Ir and (right) **X**-Ir → **XII**-Ir (scanning the carbon-carbon coordinate) (B97D3/def2svp; E, kcal·mol^{-1}; C···C, Å)

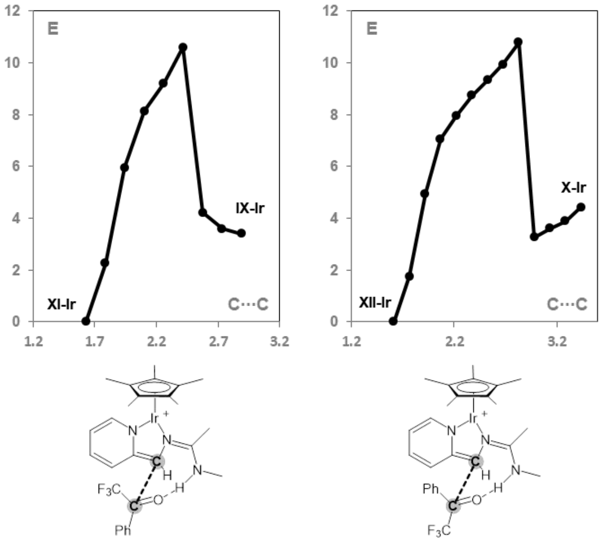

Figure S32. Energy profile for **XIV**-Rh → **11** scanning the carbon-carbon coordinate (B97D3/def2svp; E, kcal·mol^{-1}; C···C, Å)

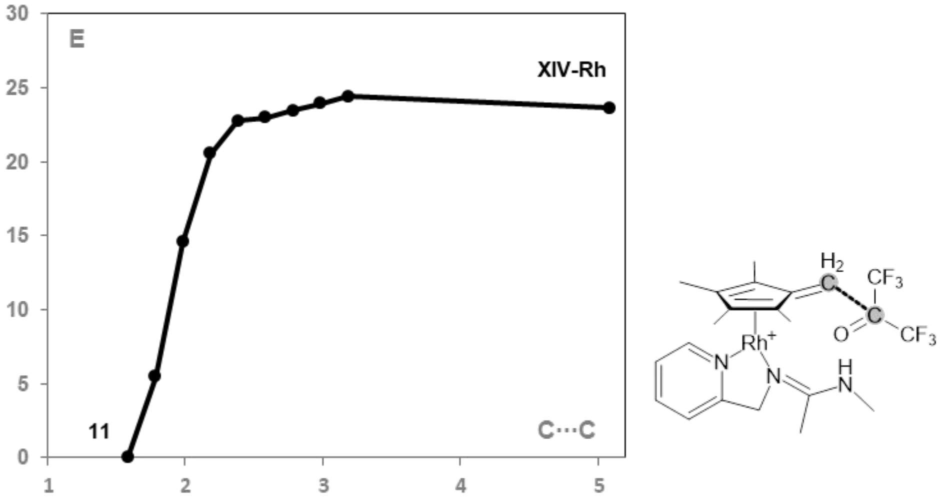

Supplement: Supplementary file 2 — ic4c03214_si_002.pdf [file ic4c03214_si_002.pdf]
